# Mapping the evidence on psychosocial interventions for migrant populations: Descriptive analysis of a living database of randomized studies

**DOI:** 10.1017/gmh.2024.33

**Published:** 2024-03-08

**Authors:** Camilla Cadorin, Marianna Purgato, Giulia Turrini, Eleonora Prina, Madalena Cabral Ferreira, Doriana Cristofalo, Monica B. Bartucz, Anke B. Witteveen, Marit Sijbrandij, Davide Papola, Corrado Barbui

**Affiliations:** 1 WHO Collaborating Centre for Research and Training in Mental Health and Service Evaluation, Department of Neuroscience, Biomedicine and Movement Sciences, Section of Psychiatry, University of Verona, Verona, Italy; 2 Public Health Unit of the Primary Care Cluster of Famalicão, Northern Region Health Administration, Famalicão, Portugal; 3 Department of Clinical Psychology and Psychotherapy, Babeș-Bolyai University, Cluj-Napoca, Romania; 4 Department of Clinical, Neuro- and Developmental Psychology, Amsterdam Public Health Institute and World Health Organization Collaborating Center for Research and Dissemination of Psychological Interventions, Vrije Universiteit Amsterdam, Amsterdam, The Netherlands; 5 Department of Global Health and Social Medicine, Harvard Medical School, Boston, MA, USA

**Keywords:** migrants, displacement, global mental health, mental distress, psychotherapy

## Abstract

Migrant mental health is a pressing public health issue with wide-ranging implications. Many randomized controlled trials (RCTs) have been conducted in this population to assess the effects of psychosocial interventions. However, the available evidence is characterized by controversy and fragmentation, with studies focusing on different migrant populations, interventions, outcomes, delivery modalities and settings. Aiming to promote systematic reviews of the effectiveness of psychosocial interventions in different migrant groups, we have developed a living database of existing RCTs. The development of the database provides an opportunity to map the existing RCT evidence in this population. A total of 135 studies involving 24,859 participants were included in the living database. The distribution of studies by year of publication aligns with the increasing global migrant population in recent years. Most studies focus primarily on adult participants, with a limited representation of children and adolescents, and a prevalence of female participants, which is consistent with epidemiological data, except for older adults, who are underrepresented in research. Studies predominantly focus on refugees and asylum seekers, likely due to their elevated risk of mental health issues, despite the substantial presence of economic migrants worldwide. While studies mainly involve migrants from the Middle East and East Asia, epidemiological data suggest a broader geographic representation, with migrants coming from Eastern Europe, Latin America and South Asia. The present descriptive analysis of RCTs on mental health and psychosocial interventions for migrant populations provides valuable insights into the existing research landscape. It should be used to inform future research efforts, ensuring that studies are more representative of the global migrant population and more responsive to the mental health needs of migrants in different contexts.

## Impact statement

The present map of randomized controlled trials on the effectiveness of psychosocial interventions in migrant populations suggests the following recommendations to guide future research: (1) future studies should include children, adolescents and older adults to better reflect current epidemiological trends; (2) research should be expanded beyond refugees and asylum seekers to include internally displaced persons, economic and other types of migrant populations; (3) more diverse migrant populations should be included, reflecting the real-world regions of origin and resettlement; (4) studies should be conducted that specifically target migrants with diagnosed mental disorders; (5) more research is needed on promotion and prevention; (6) future studies should explore the effectiveness of digital interventions and online approaches, especially for migrants who may face barriers to accessing face-to-face services; (7) studies should be conducted to better evaluate the role of nonspecialists in delivering psychosocial interventions to different migrant populations and (8) data quality and completeness in research reports should be improved to enhance the accuracy of evidence synthesis.

## Introduction

The term “migration” encompasses the process of relocating from one geographical area, whether it is a nation, region or locality, to another. This movement can transpire within the confines of a single country or entail crossing international borders, either temporarily or on a permanent basis, motivated by a myriad of factors. The impetus behind migration can be multifaceted, leading individuals to migrate either in organized groups, such as those departing their home country due to humanitarian crises or as lone individuals, as exemplified by people migrating for educational or economic pursuits. The European Psychiatric Association has categorized the reasons driving migration into two primary categories: “pull” and “push” factors, as outlined by Bhugra et al. (2014). Pull factors encompass motivations such as educational or economic opportunities and personal considerations, including family dynamics, personal economic prospects and employment prospects. Conversely, push factors include political instability, poverty, terrorism, displacement, conflict or religious influences, which serve as compelling reasons for migration. The term “migrant” is, therefore, a comprehensive umbrella concept that, although not formally codified under international legal frameworks, broadly encompasses various demographic groups. These groups include migrant workers, undocumented migrants, asylum seekers, refugees, internally displaced individuals and other populations that are in the process of moving, as elucidated in a report by the International Organization for Migration (IOM, 2019).

According to the most recent epidemiological data on migrant populations, out of the total population of international migrants worldwide of 281 million, economic migrants accounted for 60%, internally displaced persons for 20%, whereas refugees and asylum seekers accounted for 11% (IOM, 2021). The main reason for displacement was economic (60%), while humanitarian crises accounted for 14%, of which 3% was related to war and 11% to disasters. The majority of migrants were adults, accounting for 90% of the total population. Among them, 78% were between 14 and 65 years old, while 12% were over 65 years old; children and adolescents represented 15% of total international migrants. Gender was similarly distributed, with 52% males and 48% females (IOM, 2021).

Within the context of the migration process, several factors can render individuals more susceptible to a deterioration in their subjective well-being, quality of life and mental health. This heightened vulnerability, in turn, escalates the likelihood of developing mental disorders. Commonly encountered stressors and challenges during and following the migration journey encompass incongruities between expectations and achievements, limited support networks, difficulties in adaptation and acculturation processes and financial, administrative and legal complications (Sijbrandij, 2018; Mesa-Vieira et al., 2022; WHO, 2023). Forcibly displaced migrants, particularly refugees, may endure additional hardships, including the loss of homes, aspirations, possessions and disruptions in personal, familial and professional life trajectories. Preceding their migration, these individuals may have been exposed to traumatic events, such as bombings, threats, captivity, torture, injury and the witnessing of harm to loved ones (IASC, 2007; Van Ommeren and Wessells, 2007; WHO, 2022b). They are also likely to encounter significant stressors throughout the migration process. Upon arrival in host countries, a multitude of challenges persists, encompassing discrimination, financial hardships, language barriers, loss of familial and community support, limited access to social, educational and healthcare services and uncertain asylum application procedures (Sijbrandij, 2018; Jannesari et al., 2020).

Importantly, the Coronavirus Disease 2019 (COVID-19) pandemic has introduced an additional layer of stress for migrant populations, particularly impacting those in transit, who have been disproportionately affected due to weakened social support structures, diminished socioeconomic prospects, inequitable healthcare and social service access, precarious housing, precarious living and working conditions, the spread of misinformation and xenophobia, and an elevated risk of exploitation and abuse (UNDESA, 2020a; IOM, 2021; WHO, 2022a).

Epidemiological investigations have established that stressors occurring before, during and after migration play a significant role in the elevated occurrence of psychological distress and mental disorders in migrant populations (Bhugra et al., 2014; Miller and Rasmussen, 2017; Carroll et al., 2020, 2023). These occurrences differ in relation to the motives behind migration and the duration of time following resettlement. A recent comprehensive review of epidemiological studies aimed at assessing the prevalence of common mental disorders in international migrants, revealed an overall prevalence rate of 32% for post-traumatic stress disorder (PTSD), 29% for depression and 25% for anxiety disorder (Carroll et al., 2023). Notably, disparities were observed when considering voluntary and forced migrants separately. In voluntary migrants, the prevalence of common mental disorders was 11% for anxiety, 21% for depression and 6% for PTSD, while in forced migrants it was 34% for anxiety, 36% for depression and 34% for PTSD (Carroll et al., 2023). Within refugee and asylum seeker populations, a systematic review of prevalence studies disclosed a prevalence rate of 32% for depression, 31% for PTSD, 5% for bipolar disorders and 1% for psychotic disorders (Patanè et al., 2022). These findings were consistent with those reported by Blackmore et al. (2020), who additionally identified an 11% prevalence rate for anxiety disorders. Similar prevalence figures were reported for PTSD, depression and anxiety disorders among asylum seekers and refugees who resettled in high-income nations (Henkelmann et al., 2020). Migrants exposed to armed conflict also exhibited high prevalence rates, with a systematic review of 34 prevalence studies revealing a frequency of 31% for PTSD, 25% for major depression and 14% for generalized anxiety disorder (Mesa-Vieira et al., 2022). Studies involving unaccompanied refugee minors indicated a notable prevalence of mental disorders in children and adolescents. However, the prevalence rates varied substantially across studies, spanning from 4.6% to 43% for PTSD, 2.9% to 61.6% for depression, 32.6% to 38.2% for anxiety and 4% to 14.3% for behavioral problems (Hutchinson et al., 2022).

In recent years, an increasing number of randomized clinical trials (RCTs) have investigated the efficacy and/or effectiveness of psychosocial interventions aimed at improving mental health and functioning in migrant populations. Recent research has also explored innovative delivery approaches, including the involvement of nonspecialist facilitators, community workers and primary-level healthcare personnel to facilitate intervention delivery (WHO, 2008; Patel et al., 2018; Barbui et al., 2020; van Ginneken et al., 2021). This shift in delivery methods may hold particular significance in resource-constrained settings, especially within low- and middle-income countries (LMICs) (Singla et al., 2017; Patel et al., 2018; Barbui et al., 2020). Nevertheless, the available evidence is marked by controversy and fragmentation, with studies focusing on various migrant populations, interventions, outcomes, delivery modalities and settings. This diversity makes it challenging to comprehensively evaluate the entire body of evidence using a uniform metric and methodological framework. Additionally, concerns about the quality of the evidence, including issues related to data collection completeness and the accuracy of reporting, pose difficulties as they influence the reliability of effectiveness estimates.

Important questions that have not been properly addressed include the following: (1) Which psychosocial interventions have been shown to promote mental health and prevent the development of mental health conditions in different migrant populations such as migrant workers, undocumented migrants, asylum seekers, refugees and internally displaced persons? (2) Which psychosocial interventions are effective in treating mental disorders in different migrant populations such as migrant workers, undocumented migrants, asylum seekers, refugees and internally displaced persons? (3) Are some psychosocial interventions more effective than others in terms of promotion, prevention and treatment efficacy? (4) Which delivery formats are supported by the evidence (individual in person, individual online synchronous, group in person, group online synchronous, self-help in person, self-help online with or without facilitator)? (5) Are task-shifting delivery modalities supported by the evidence?

Aiming to answer these research questions, we developed a living database of RCTs of psychosocial interventions in different types of migrant populations. As this living database includes a wide range of migrant populations, different interventions and comparison groups and several outcome measures, it covers multiple participants, intervention, comparator, outcome (PICOs) simultaneously. The aggregation of multiple PICOs has recently been defined as “Meta-Analytic Research Domain” (MARD) by Cuijpers et al. (2022), who underlined the relevance of covering broad fields of interest with the aim of conducting living systematic reviews.

Against this background, the aim of this report is to map the evidence from RCTs on the efficacy of psychosocial interventions. This analysis is particularly pertinent in assessing whether the migrant populations included in the experimental research align with the global epidemiological figures on migrant populations and migration patterns.

## Methods

This mapping review (Bates et al., 2007; Cooper, 2016; Miake-Lye et al., 2016) is a component of a larger project aimed at developing an MARD focused on RCTs investigating psychosocial interventions for migrant populations (Supplement 1). The project’s protocol is registered on Open Science Framework (https://osf.io/jd3zn), and database searches are being updated on an annual basis. We followed the Cochrane Handbook for Systematic Reviews of Interventions (Higgins et al., 2022) and the Preferred Reporting Items for Systematic Reviews and Meta-analyses guidelines for data reporting (Page et al., 2021).

### Identification and selection of studies

We conducted searches in PubMed, PsycINFO, MEDLINE (Ovid), Web of Science, Cochrane Central Register of Controlled Trials (CENTRAL), Pilots PTSDpubs (ProQuest), CINAHL, Scopus and Embase, from database inception to January 1, 2023. Electronic database searches were supplemented by a manual search of reference lists from relevant systematic reviews and meta-analyses related to this topic. The full search strategy is reported in Supplement 2. No language or publication type restrictions were applied.

Studies meeting the following criteria were included: (1) implementing an RCT study design; (2) including migrants of any age, ethnicity and religion; (3) assessing the efficacy of any type of promotion, prevention and treatment interventions with a main psychosocial component; (4) comparing psychosocial interventions with interventions like treatment as usual, defined as any intervention that reflects the usual care in a given treatment setting, no treatment, waiting list or any other psychosocial interventions and (5) reporting as primary or secondary outcome at least one of the following mental health outcomes: anxiety, depression, PTSD and psychological distress.

The International Organization for Migration (IOM) definition of migrants was followed, including a variety of different population groups such as asylum seekers, refugees, internally displaced persons, unaccompanied minors, economic migrants, other populations on the move and any other type of forced or unforced migrants (Bhugra et al., 2011; Bhugra et al., 2014; Abubakar et al., 2018; IOM, 2019). Studies with second-generation migrants were excluded unless they constituted a minority of the randomized migrant participants (less than 20%). Migrants with or without any physical or mental health conditions were included. Psychosocial interventions were defined in accordance with Inter-Agency Standing Committee (IASC) guidelines as “mental health and psychosocial support” (MHPSS) (IASC, 2007; Miller et al., 2021), which is a composite term used to describe “any type of local or outside support that aims to protect or promote psychosocial well-being and/or prevent or treat mental disorders” (IASC, 2007, p. 822). Interventions are, thus, generally classified into promotion, prevention and treatment, based on their aim and target (Institute of Medicine, 1994; Tol et al., 2015; Eaton, 2019). Prevention is an approach aiming at reducing the likelihood of developing mental disorders; it is further divided into three subcategories, namely universal, selective and indicated prevention (IOM, 1994; Eaton, 2019; National Academies of Sciences, Engineering, and Sciences, 2019; WHO, 2022a). Universal prevention targets the whole population, whereas selective prevention addresses specific categories of people with heightened risk, as being vulnerable populations. On the other hand, indicated prevention specifically aims at high-risk people showing increased levels of distress and symptoms that could lead to a diagnosis. Prevention is, therefore, the intermediate step between promotion and treatment, with promotion aiming at strengthening psychological well-being and protective factors (self-esteem, resilience, prosocial behavior), and treatment aiming at reducing symptoms in case of a probable or confirmed diagnosis. The included psychosocial interventions were delivered by professionals and nonprofessionals, including primary-level health workers, community workers, trained facilitators or lay-helpers with and without a mental health background. We included psychosocial interventions delivered through any means of interaction, including face-to-face in-person sessions, face-to-face digital synchronous sessions and digital asynchronous sessions. Both individual and group interventions were eligible for inclusion, provided by any type of staff, or self-administered. We did not set a limit for the duration and the number of sessions. We included RCTs conducted in any country, irrespective of income level and in any setting (healthcare and clinical settings as well as community settings such as refugee camps, schools, social care settings and any other community setting).

### Data collection, extraction and presentation

Two review authors (C.C., D.C., E.P., M.C.F.) independently assessed titles, abstracts and full texts of potentially relevant articles, and extracted data following the recommendations of the Cochrane Handbook for Systematic Reviews of Interventions (Higgins et al., 2022). Any disagreement was resolved by consensus or by arbitration by a senior researcher (G.T., M.P.). Following a PICO framework, we extracted data about the sociodemographic characteristics of participants (e.g., age, gender, migration status, country of origin and resettlement, years since resettlement, diagnosis and/or common mental health symptoms, namely anxiety, depressive and post-traumatic stress symptoms), intervention and control (e.g., type, classification, duration, format, sessions) and outcomes: anxiety symptoms, depression symptoms, level of psychological distress, PTSD symptoms, psychological functioning and/or impairment, psychological well-being, quality of life and rates of all-cause trial discontinuation (acceptability outcome). Descriptive analyses were conducted to summarize the study- and participant-level characteristics.

Two review authors independently assessed the risk of bias applying Cochrane Risk of Bias 2 tool and using the criteria outlined in the Cochrane Handbook for Systematic Reviews of Interventions, Chapter 8 (Higgins et al., 2022). We resolved disagreements by discussion or by consultation with a third review author.

## Results

### Study selection and participant characteristics

**Figure 1.**
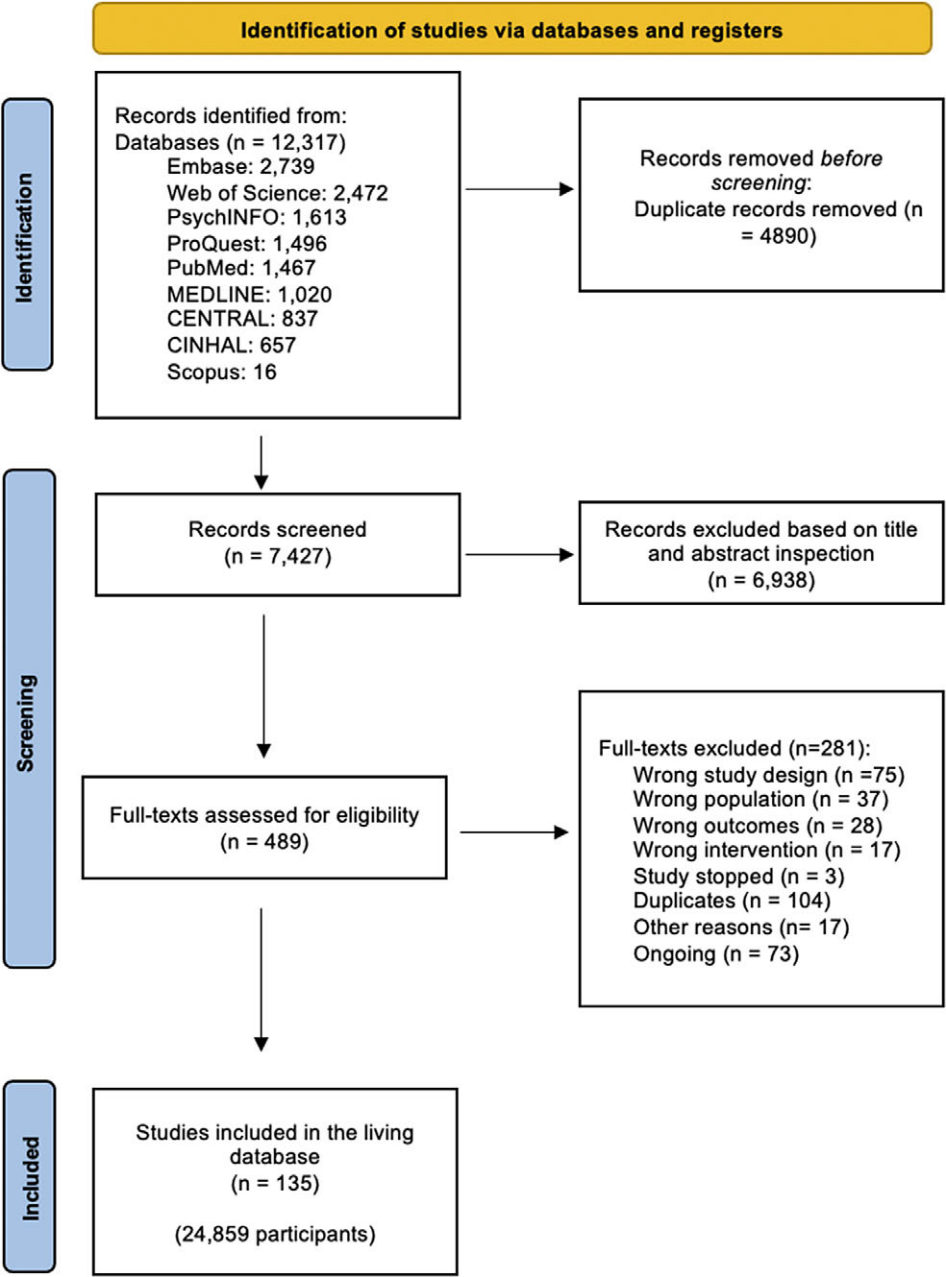
Preferred Reporting Items for Systematic Reviews and Meta-analyses (PRISMA) flowchart.

The search identified 12,317 records. After removing duplicates and examining titles and abstracts, we selected 489 records for full-text assessment. Of these, 354 were excluded for the reasons reported in Figure 1, and 135 studies, including 24,859 participants, were eligible for inclusion in the MARD database (Figure 1; Supplement 1; Supplementary Table S1-S2). Of 135 included studies, 47 (35%) were published before 2015, 49 (36%) between 2015 and 2020 and 39 (29%) from 2021 until January 2023, with an average of four studies per year (Supplementary Table S3). In terms of quality, 70 studies were assessed for anxiety, 107 for depression, 85 for PTSD and 28 for distress (Supplementary Table S5). Ten studies were at high risk of bias for anxiety, 16 for depression, nine for PTSD and five for psychological distress; 48 studies were evaluated as having “some concerns” for anxiety, 75 for depression, 60 for PTSD and 22 for distress and 12 studies were considered at low risk of bias for anxiety, 16 for depression, 16 for PTSD and one for distress.

Most studies included adults (*k* = 104, 77%), with only few studies conducted on children and adolescents (*k* = 21, 16%), and a minority (*k* = 10, 7%) on mixed populations of children and adults, usually their parents or caregivers (Table 1). A total of 78 studies (58%) included a larger percentage of women, whereas in 47 studies (35%) the majority of participants were men. Participants were mostly refugees and asylum seekers (*k* = 91, 67%), with refugees alone accounting for 47% of studies. On the other hand, 36 studies (27%) included economic and other types of migrants and the remaining included internally displaced persons (*k* = 8, 6%). Most studies reported humanitarian crises (*k* = 103, 76%) as the reason for displacement, with only 5% (*k* = 6) reporting economic reasons; for 19% (*k* = 26) reasons for displacement were not specified. Humanitarian crises included war (39%), general humanitarian crises (21%), conflicts (7%) and genocides (2%). A high proportion of studies did not specify the time since resettlement (*k* = 57, 42%), but in the studies in which it was clearly stated, most participants had been in the host country for more than 5 years at the time of recruitment (*k* = 31, 23%).

**Table 1.** Characteristics of included studies

Population							
Age group	*N* studies (%)	Target group	*N* studies (%)	Reason for displacement	*N* studies (%)	Time since resettlement	*N* studies (%)
Adults (≥18) Children/adolescents (<18) Mixed (children and adults)	104 (77%) 21 (16%) 10 (7%)	Refugees/asylum seekers Internally displaced persons Other types of migrants	91 (67%) 8 (6%) 36 (27%)	Humanitarian crisis Work/family Not specified	103 (76%) 6 (5%) 26 (19%)	<1 year 1–5 years >5 years Not specified Mixed	5 (4%) 17 (13%) 31 (23%) 57 (42%) 25 (19%)
Intervention							
Goal	*N* studies (%)	Type of intervention	*N* studies (%)	Delivery level and format	*N* studies (%)	Delivered by	*N* studies (%)
Promotion Universal prevention Selective prevention Indicated prevention Treatment	0 (0%) 0 (0%) 31 (23%) 37 (27%) 67 (50%)	Psychotherapy Counseling/psychosocial support Creative–expressive activity Family/parenting support	49 (36%) 58 (43%) 12 (9%) 16 (12%)	*Level* Group Individual Mixed *Format* Face–to–face Online Mixed	66 (49%) 63 (47%) 6 (4%) 116 (86%) 16 (12%) 3 (2%)	Professional Nonprofessional Not specified Mixed Other (online)	50 (37%) 61 (45%) 8 (6%) 12 (9%) 4 (3%)
Control				Setting and publication year			
Number of study arms	*N* studies (%)	Control condition	*N* studies (%)	Setting	*N* studies (%)	Publication year	*N* studies (%)
Two Three Four	114 (84%) 16 (12%) 5 (4%)	Psychosocial intervention Attentional placebo (Enhanced) treatment as usual No treatment Psychological placebo Waiting list	35 (26%) 7 (5%) 37 (27%) 11 (8%) 6 (4%) 65 (48%)	Clinical Community Home/online Refugee camp School Not specified Mixed/other	46 (34%) 22 (16%) 19 (14%) 15 (11%) 11 (8%) 14 (11%) 8 (6%)	2021 – today 2015–2020 Before 2015	39 (29%) 49 (36%) 47 (35%)
Outcomes							
Common mental disorders – symptoms	*N* studies (%)	Positive mental health	*N* studies (%)	Functioning	*N* studies (%)	Follow–up	*N* studies (%)
Post–traumatic stress Anxiety Depression Psychological distress	84 (62%) 69 (51%) 107 (79%) 26 (19%)	Well–being Quality of life	22 (16%) 19 (14%)	General Relational Functional impairment Psychosocial	2 (1%) 9 (7%) 21 (16%) 6 (4%)	0–1 months 1–6 months 7–24 months	120 (89%) 14 (10%) 1 (1%)

### Study participants’ mental health problems

At baseline, the majority of studies did not select the migrant population based on the presence of mental disorders or on the presence of scoring above a threshold of mental health symptoms. No studies included participants based on a diagnosis of anxiety disorder, and only two studies (1%) included participants with anxiety symptoms. Then, 8% of studies (*k* = 11) included participants with a diagnosis of depression at baseline and 8% (*k* = 11) with depressive symptoms (Table 1). A total of 45 studies (33%) included participants with a diagnosis of PTSD and seven (5%) with PTSD symptoms at baseline. Distress symptoms were present in 11 studies (8%) and 15 studies (11%) included participants without symptoms at baseline. In terms of primary and secondary outcomes, 107 studies (79%) assessed depressive symptoms, 84 studies (62%) PTSD, 70 (52%) anxiety symptoms and 26 (19%) psychological distress in general. Regarding positive mental health outcomes, 26 studies (19%) and 19 (14%) assessed well-being and quality of life, respectively (Table 1). Functioning was measured by 36 studies (26%), with functional impairment as the most represented subtype (*k* = 20, 15%), followed by relational functioning (*k* = 11, 8%). Almost all studies (*k* = 120, 89%) assessed outcomes within 1 month after the end of the intervention, 14 studies (10%) in the timeframe from 1 to 6 months, and only one study (1%) from 7 to 24 months post-intervention (Table 1).

### Type of interventions

Half of the studies (*k* = 67, 50%) were classified as treatment, 37 (27%) as indicated prevention and 31 (23%) as selective prevention (Table 1). The most represented type of intervention was counseling and psychosocial support (*k* = 58, 43%), followed by psychotherapy (*k* = 49, 36%). Family/parenting support interventions accounted for 12% (*k* = 16), and 12 studies (9%) focused on creative/expressive interventions (see Table 1 and Supplementary Table S4 for the full list of interventions). Psychosocial support alone accounted for 36% (*k* = 48), whereas counseling for 7% (*k* = 10). Within this category, World Health Organization (WHO) psychosocial interventions (Problem Management Plus, Self Help Plus, Doing What Matters in time of stress, and Early Adolescent Skills for Emotions) represented 11% of interventions. Deepening into the specific type of psychotherapies, cognitive behavioral therapy (CBT)-based/inspired interventions were implemented in 21 studies (16%), of which traditional CBT represented 9%, and trauma-focused psychotherapies were represented in 20 studies (15%). Among trauma-focused interventions, narrative exposure therapy emerged as the most frequently examined (9% of studies).

Regarding the delivery of interventions, group and individual levels were similarly represented (*k* = 63 studies, 47%, and *k* = 66 studies, 49%, respectively); most interventions were delivered face-to-face (*k* = 116, 86%), with only a minority delivered online. People who delivered interventions were both nonspecialists (*k* = 61, 45%) and mental health professionals (*k* = 50, 37%). Nonspecialists included trained volunteers/facilitators (30%), community workers (5%), paraprofessionals (5%), primary-level healthcare workers (1%) and trained facilitators with mental health backgrounds (3%). Interventions were delivered mostly in clinical settings (*k* = 46, 34%), whereas other settings were similarly represented: community (*k* = 22, 16%), home/online (*k* = 19, 14%), refugee camp (*k* = 15, 11%), school (*k* = 11, 8%) and not specified (*k* = 14, 11%). The duration of interventions ranged from one single session to 60 weeks, with an average of 11 weeks and a median of 8 weeks. The number of sessions ranged from 1 to 46, with an average of 10 sessions and a median of 8.

### Study participants’ country of origin and resettlement

**Figure 2.**
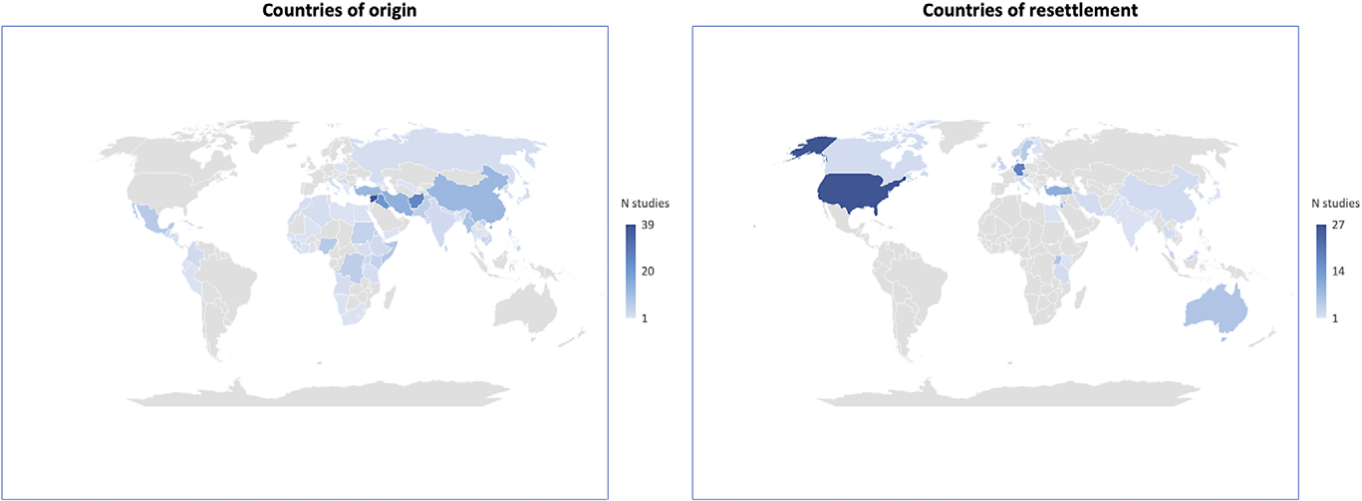
Country of origin and country of resettlement.

Participants in the MARD database mostly originated from the Middle East (*k* = 29, 22%), followed by East Asia (*k* = 23, 17%). Many studies (*k* = 34, 25%) included migrants from mixed countries (Figure 2). The remaining studies had all percentages under 10%, specifically, Sub-Saharan Africa (10%), Latin America (7%), South Asia (7%), Europe (4%) and Central Asia (3%). At the country level, Syria alone accounted for 19% of studies, followed by China (6%), Myanmar and Afghanistan (4%). The vast portion of the participants’ countries of origin (*k* = 49, 37%) falls within the low-income category based on the World Bank classification for countries’ income (WB, 2022). Middle-income countries accounted for 30% (*k* = 40 studies; specifically, *k* = 16, 12% lower-middle, *k* = 18, 13% upper-middle and *k* = 6, 4% mixed), whereas only 1% of studies (*k* = 2) included participants from high-income countries (Figure 2). However, in a relevant proportion of studies, the country of origin was mixed (*k* = 37, 27%). As for asylum seekers and refugees, Syria (19%) was the most represented country of origin, followed by Afghanistan, Myanmar and Cambodia.

According to the studies included in the living database, the main destination of international migrants was Europe (*k* = 49, 36%), followed by North America (*k* = 28, 21%) and the Middle East (*k* = 15, 11%). At the country level, the most involved countries were the United States (19%), Germany (12%), Turkey (7%) and Jordan (6%). Most studies were conducted in high-income countries (*k* = 89, 66%), followed by upper-middle-income countries (*k* = 30, 22%), while seven studies (5%) were conducted in lower-middle-income countries and nine (7%) in low-income countries. For the group of refugees and asylum seekers, most studies were conducted in high-income countries, with the United States (10%) and Germany (9%) being the most represented countries (Figure 2).

## Discussion

The aim of this descriptive analysis was to comprehensively map the existing RCT evidence on the efficacy of psychosocial interventions for migrant populations, allowing comparisons to be made between the characteristics of these studies and the global epidemiological figures on migrant populations and migration flows.

Our study selection process revealed a moderate volume of research in this domain, with 135 studies involving a total of 24,859 participants included in the living database. Notably, the distribution of studies over time appeared to align with the evolving public health impact of migrant populations, with an increasing number of migrants worldwide in recent years (UNDESA, 2020a; IOM, 2021). This correlation highlights the growing importance of understanding and addressing the psychosocial needs of migrants (Uphoff et al., 2020).

The majority of studies primarily focused on adult participants, with limited representation of children and adolescents. Additionally, a substantial portion of studies included a higher proportion of women than men. These study findings appear to be in good agreement with epidemiological data on migrant populations (UNDESA, 2020a, 2020b; IOM, 2021, UNHCR, 2021). However, it is worth noting that we identified a limited number of studies examining older adults, even though United Nations (UN) data from 2021 (UNDESA, 2020a) indicate that older individuals constitute a relevant portion of migrants.

In terms of migrant populations, the majority consisted of refugees and asylum seekers, with refugees alone accounting for half of the studies. Economic migrants and internally displaced persons were less commonly studied, reflecting the predominant focus on forced migration. This would suggest an over-representation of studies on refugees as compared with epidemiological figures on different types of migrants, which showed a predominance of economic migrants (UNDESA, 2020a, 2020b; IOM, 2021, IDMC, 2022, UNHCR, 2021). A possible explanation for this discrepancy may be that refugees and asylum seekers are a particularly hig-risk population for psychological distress and mental disorders compared to other migrant groups (Mesa-Vieira et al., 2022).

Participants in the studies primarily originated from the Middle East and East Asia, reflecting the regions with a high number of forced migrants. However, there was limited representation of migrants from other regions, such as South Asia, Latin America and Eastern Europe, which is inconsistent with UN data (UNDESA, 2020a, 2020b). Similarly, while Europe and North America were common destinations in the included studies, the UN data (UNDESA, 2020a, 2020b) indicated that migrants often resettled in other regions, such as South Asia and Latin America. This is particularly the case for refugees and asylum seekers, who have tended to be resettled in low- or middle-income countries, while studies on refugees have been conducted mainly in high-income countries (Nosè et al., 2017; Sijbrandij, 2018; Turrini et al., 2019; Soltan et al., 2022).

Notably, very few studies selected participants based on the presence of a diagnosis of mental disorders, while the majority enrolled populations with high levels of psychological symptoms with a focus on indicated prevention (Tol et al., 2015). Although this is in line with epidemiological data on psychological distress levels in migrant populations, we note that PTSD, depression and anxiety were quite prevalent among migrants, with PTSD being the most common diagnosis (Blackmore et al., 2020; Patanè et al., 2022; Carroll et al., 2023). This highlights the need for further research to address the mental health challenges faced by migrants with established psychiatric diagnoses and other serious mental disorders such as psychosis or bipolar disorder (Brandt et al., 2019). On the other hand, as highlighted also by Uphoff et al. (2020), there is a lack of studies on promotion and selective prevention of common mental disorders.

Different types of interventions were explored in the randomized studies, with counseling and psychosocial support interventions being the most common. Psychotherapy and family/parenting support interventions were also widely studied. Interventions were delivered through both group and individual approaches, primarily in face-to-face settings, which may not always be feasible and economically sustainable (WHO, 2018, 2020). Studies investigating the effectiveness of digital interventions or interventions delivered using synchronous online approaches are still lacking and represent an important gap in the current literature (Uphoff et al., 2020). The providers of interventions in included studies were both nonspecialists and mental health professionals. The heterogeneous delivery settings, along with the mix of providers, underscore the adaptability of psychosocial interventions to different contexts.

Despite its findings, this review has some limitations. First, for many important variables in the database, data were often missing, such as for time since resettlement, reason for displacement and intervention setting, which makes the synthesis of evidence less accurate. Second, according to the conceptual framework of the MARD, different migrant populations were considered. In particular, the inclusion of studies on economic and other types of migrants in the same database as studies on refugees, asylum seekers and displaced persons can be problematic, as economic migrants can include both highly vulnerable populations fleeing structural violence and crime, high levels of poverty and economic instability, as well as educated students or workers who choose to live and work abroad. We note, however, that stressors associated with the migration process, regardless of the reasons behind it, may have a significant role in the elevated occurrence of psychological distress in migrant populations (Bhugra et al., 2014; Miller and Rasmussen, 2017; Sijbrandij, 2018; Carroll et al., 2020; Mesa-Vieira et al., 2022; Carroll et al., 2023). All the studies included in this MARD therefore share migration as a social determinant of mental health. Clearly, any additional risk factors that may be present in the included studies will need to be carefully considered by the systematic reviews that are conducted and, depending on their aims, some populations may be included or excluded or subgroup or sensitivity analyses may be performed to investigate heterogeneity related to the type of populations included.

This comprehensive analysis of randomized studies on psychosocial interventions for migrant populations provides valuable insights into the existing research landscape. It underscores the importance of addressing the mental health needs of migrants, particularly in light of the disparities between the characteristics of study participants and real-world migrant populations. These findings can inform future research efforts, ensuring that interventions are better aligned with the mental health challenges faced by migrants in diverse contexts and that studies are more representative of the global migrant population. Research should first focus on psychosocial interventions already available and developed for this context, both in terms of their implementation, scalability, feasibility, cultural and contextual appropriateness, as well as clinical and cost-effectiveness. This would help to improve the field of research by focusing on the most relevant interventions that have led to substantial mental health gains for specific migrant populations. More specifically, the following recommendations can be made to guide future research:

1. Addressing underrepresented age groups: future studies should investigate the psychosocial needs of children, adolescents and older adults among migrant populations to ensure a comprehensive understanding and tailored interventions (Uphoff et al., 2020; Soltan et al., 2022).
2. Diversifying study populations: research should be expanded beyond refugees and asylum seekers to include economic and other types of migrants and internally displaced people, recognizing the different exposure to risk factors and the varying needs of different migrant groups (Uphoff et al., 2020; Hasan et al., 2021).
3. Global representation: more diverse migrant populations should be included in research, reflecting the regions of origin and resettlement beyond the Middle East and East Asia. More attention should be paid to the mental health needs and interventions of migrants from and in regions such as South Asia, Eastern Europe and Latin America, where they often start and end their migratory process.
4. Diagnostic focus: in addition to research targeted to migrant populations with elevated psychological distress, studies should be conducted that specifically target migrants with diagnosed mental disorders, such as PTSD, depression and anxiety disorders, as well as other mental health conditions (i.e., bipolar disorder, psychosis). Particular consideration should be given to the use of diagnostic categories in culturally diverse groups, where the use of diagnostic categories may be problematic due to different symptom constellations that do not necessarily correspond to the diagnostic categories specified in Western diagnostic manuals.
5. Promotion and prevention studies: future research should also explore the effectiveness of interventions aiming at enhancing the awareness on mental health issues and promoting positive mental health, by strengthening psychological well-being, resilience, coping and prosocial behavior, among others. Also, there is a need to test and develop selective and indicated prevention interventions, focused on preventing the onset of disorders in populations not screened for diagnoses, or without any symptoms.
6. Exploring digital interventions: future studies should explore the effectiveness of digital interventions and online approaches for psychosocial support, especially for migrants who may face barriers to accessing face-to-face services.
7. Provider and delivery diversity: studies should evaluate the roles of nonprofessionals and mental health professionals in delivering psychosocial interventions to different migrant populations and adapting intervention settings to suit various contexts.
8. Data quality and real-time updates: data quality and completeness in research reports should be improved to enhance the accuracy of evidence synthesis (Uphoff et al., 2020). More accurate situational analyses should be developed to incorporate more up-to-date data on migration flows into the design of clinical trials to ensure research aligns with real-world trends.

These recommendations can guide future research efforts to better understand and address the psychosocial needs of migrant populations. Ultimately, bridging these gaps can contribute to more effective and targeted psychosocial interventions for this vulnerable and diverse population.

## Supporting information

Cadorin et al. supplementary material Cadorin et al. supplementary material

## Data Availability

Data availability is not applicable to this article as no new data were created or analyzed in this study.

## Supplementary material

The supplementary material for this article can be found at http://doi.org/10.1017/gmh.2024.33.
