# Supplementary material for: Mapping the evidence on psychosocial interventions for migrant populations: Descriptive analysis of a living database of randomized studies
Source: Glob Ment Health (Camb). 2024 Mar 8;11:e35. doi: 10.1017/gmh.2024.33 (PMC10988138; doi:10.1017/gmh.2024.33)
Supplement: Cadorin et al. supplementary material [file S2054425124000335sup001.docx]

**Mapping the evidence on psychosocial interventions for migrant populations:**

**descriptive analysis of a living database of randomized studies**

**SUPPLEMENTAL MATERIAL**

**Summary page**

Supplement 1: PICO for eligibility criteria 3

Supplement 2: Search strategy 4

Table S1: References of the included studies identified by the search strategy 10

Table S2: List of excluded studies with reasons 18

Table S3: Graphs publication year – international migrants 38

Table S4: Classification of Interventions 39

Table S5: RoB-2 assessment for anxiety, depression, PTSD, and psychological distress 41

**Supplement 1: PICO for eligibility criteria**

| **Population** | Children and adult asylum seekers, refugees, internally displaced persons, unaccompanied minors, economic migrants, other populations on the move, and any other type of forced or unforced migrants (IOM, 2019) |
| --- | --- |
| **Intervention** | Promotion, prevention, and treatment psychosocial interventions, defined as any type of local or external support that aims to protect or promote psychosocial well-being and/or prevent or treat mental disorders (IASC, 2017) |
| **Comparison** | Treatment as usual (TAU), no treatment, waiting list (WL), any other psychosocial interventions |
| **Outcome** | (1) Anxiety  (2) Depression  (3) PTSD  (4) Psychological distress |
| **Setting** | Any |

**Supplement 2: Search strategy**

1. **PUBMED:**

|  |  |
| --- | --- |
| #1 | ((trial[Title/Abstract]) OR (random*[Title/Abstract])) OR (control*[Title/Abstract]) |
| #2 | ((((migrant*[Title/Abstract]) OR (immigrant*[Title/Abstract])) OR (refugee*[Title/Abstract])) OR (asylum seeker*[Title/Abstract])) OR (displaced[Title/Abstract]) |
| #3 | (((((psychotherapy[Title/Abstract]) OR (psychological[Title/Abstract])) OR (psychosocial[Title/Abstract])) OR (intervention[Title/Abstract])) OR (support[Title/Abstract])) OR (program*[Title/Abstract]) |
| #4 | ((((((((((((((mental[Title/Abstract]) OR (disorder*[Title/Abstract])) OR (distress[Title/Abstract])) OR (PTSD[Title/Abstract])) OR (trauma*[Title/Abstract])) OR (depress*[Title/Abstract])) OR (anxiety[Title/Abstract])) OR (anxious[Title/Abstract])) OR (post traumatic stress[Title/Abstract])) OR (somatic[Title/Abstract])) OR (psychotic[Title/Abstract])) OR (quality of life[Title/Abstract])) OR (well being[Title/Abstract])) OR (functioning[Title/Abstract]) OR (mental health[Title/Abstract]) OR (dropouts[Title/Abstract])) |
| **#5** | **#1 AND #2 AND #3 AND #4**  Or  **(((((trial[Title/Abstract]) OR (random*[Title/Abstract])) OR (control*[Title/Abstract])) AND (((((migrant*[Title/Abstract]) OR (immigrant*[Title/Abstract])) OR (refugee*[Title/Abstract])) OR (asylum seeker*[Title/Abstract])) OR (displaced[Title/Abstract]))) AND ((((((psychotherapy[Title/Abstract]) OR (psychological[Title/Abstract])) OR (psychosocial[Title/Abstract])) OR (intervention[Title/Abstract])) OR (support[Title/Abstract])) OR (program*[Title/Abstract]))) AND (((((((((((((((mental[Title/Abstract]) OR (disorder*[Title/Abstract])) OR (distress[Title/Abstract])) OR (PTSD[Title/Abstract])) OR (trauma*[Title/Abstract])) OR (depress*[Title/Abstract])) OR (anxiety[Title/Abstract])) OR (anxious[Title/Abstract])) OR (post traumatic stress[Title/Abstract])) OR (somatic[Title/Abstract])) OR (psychotic[Title/Abstract])) OR (quality of life[Title/Abstract])) OR (well being[Title/Abstract])) OR (functioning[Title/Abstract]) OR (mental health[Title/Abstract]) OR (dropouts[Title/Abstract])))** |

Sort by: **Most Recent**

1. **PSYCINFO**

| S1 | trial OR random* OR control* |
| --- | --- |
| S2 | psychotherapy OR psychological OR psychosocial OR intervention OR program* OR support |
| S3 | mental OR disorder* OR distress OR PTSD OR trauma* OR depress* OR anxiety OR anxious OR somatic OR psychotic OR post traumatic OR quality of life OR well–being OR functioning OR mental health OR dropouts |
| S4 | migrant* OR immigrant* OR refugee* OR asylum seeker* OR displaced |

| **S5** | **((migrant* or immigrant* or refugee* or asylum seeker* or displaced) and**  **(mental or disorder* or distress or PTSD or trauma* or depress* or anxiety**  **or anxious or somatic or psychotic or post traumatic or quality of life or**  **well–being or functioning or mental health or dropout) and**  **(psychotherapy or psychological or psychosocial or intervention or**  **program* or support) and (trial or random* or control*))mp. [mp=title,**  **abstract, heading word, table of contents, key concepts, original title,**  **tests and measures, mesh word]** |
| --- | --- |

1. **MEDLINE (piattaforma OVID):**

Migrant*

Immigrant*

Refugee*

Asylum seeker*

displaced

**1 OR 2 OR 3 OR 4 OR 5**

Psychotherapy

Psychological intervention

Psychosocial

Intervention

Program*

Support

**7 OR 8 OR 9 OR 10 OR 11 OR 12**

Mental

Disorder*

Distress

PTSD

Trauma*

Depress*

Anxiety

Anxious

Post traumatic stress

Somatic

Psychotic

Quality of life

Well being

Functioning

Mental health

Dropouts

**14 OR 15 OR 16 OR 17 OR 18 OR 19 OR 20 OR 21 OR 22 OR 23 OR 24 OR 25 OR 26 OR 27 OR 28 OR 29**

Random*

Control*

Trial

**31 OR 32 OR 33**

**6 AND 13 AND 30 AND 34**

1. **WEB OF SCIENCE (all databases)**

Databases= WOS, BCI, CCC, DRCI, DIIDW, KJD, MEDLINE, RSCI, SCIELO, ZOOREC

Timespan=All years; Search language=Auto

| # 1 | AB=(migrant* OR immigrant* OR refugee* OR asylum seeker* OR displaced*) |
| --- | --- |
| # 2 | AB=(Psychotherapy OR Psychological intervention OR Psychosocial OR Intervention OR Program* OR support) |
| # 3 | AB=(Mental OR Disorder* OR Distress OR PTSD OR Trauma* OR Depress* OR Anxiety OR Anxious OR Post traumatic stress OR Somatic OR Psychotic OR Quality of life OR Well being OR functioning OR Mental health OR Dropouts) |
| # 4 | AB=(Random* OR Control* OR Trial) |

| **# 5** | **#4 AND #3 AND #2 AND #1** |
| --- | --- |

1. **COCHRANE CENTRAL REGISTER OF CONTROLLED TRIALS (CENTRAL)**

#1

(immigrant*):ab OR (migrant*):ti,ab,kw OR (refugee*):ti,ab,kw OR (asylum seeker*):ti,ab,kw AND (displaced):ti,ab,kw

#2

(psychotherapy):ab OR (psychological):ti,ab,kw OR (psychosocial):ti,ab,kw OR (intervention):ti,ab,kw OR (program*):ti,ab,kw

#3

(random*):ab OR (control*):ti,ab,kw OR (trial):ti,ab,kw

#4

(mental):ab OR (disorder*):ti,ab,kw OR (distress):ti,ab,kw OR (trauma*):ti,ab,kw OR (PTSD):ti,ab,kw OR (depress*):ab OR (anxiety):ti,ab,kw OR (anxious):ti,ab,kw OR (somatic):ti,ab,kw OR (psychotic):ti,ab,kw OR (quality of life):ab OR (well–being):ti,ab,kw OR (functioning):ti,ab,kw OR (post traumatic stress):ti,ab,kw OR (post–traumatic stress):ti,ab,kw OR (mental health):ti,ab,kw OR (dropouts):ti,ab,kw

**#5:**

**#1 AND #2 AND #3 AND #4**

OR

#1

(mental OR disorder* OR distress OR PTSD OR trauma* OR depress* OR anxiety OR anxious OR somatic OR psychotic OR quality of life OR well–being OR functioning OR post traumatic stress OR post–traumatic stress OR mental health OR dropouts)

#2

(migrant* OR immigrant* OR refugee* OR asylum seeker* OR displaced)

#3

(psychotherapy OR psychological OR psychosocial OR intervention OR program* OR support)

#4

(trial OR random* OR control*)

**#5**

**#1 AND #2 AND #3 AND #4**

1. **PTSDpubs (ex PILOTS) in ProQuest**

**S1**

AB,TI(refugee* OR asylum seeker* OR migrant* OR immigrant* OR displace*)

**S2**

AB,TI(Psychotherapy OR Psychological intervention OR Psychosocial OR Intervention OR Program* OR Support)

**S3**

AB,TI(random* OR control* OR trial)

**S4**

AB,TI(Mental OR Disorder* OR Distress OR PTSD OR Trauma* OR Depress* OR Anxiety OR Anxious OR Post traumatic stress OR Somatic OR Psychotic OR Quality of life OR Well being OR Functioning [OR Mental health OR Dropouts](https://www.proquest.com/recentsearches.recentsearchtabview.recentsearchesgridview.scrolledrecentsearchlist.checkdbssearchlink:rerunsearch/E4041DCFE5A440DCPQ/None?t:ac=RecentSearches))

**S5**

**1 AND 2 AND 3 AND 4**

OR

**AB,TI(refugee* OR asylum seeker* OR migrant* OR immigrant* OR displace*) AND AB,TI(Psychotherapy OR Psychological intervention OR Psychosocial OR Intervention OR Programme OR Support) AND AB,TI(random* OR control* OR trial) AND AB,TI(Mental OR Disorder* OR Distress OR PTSD OR Trauma* OR Depress* OR Anxiety OR Anxious OR Post traumatic stress OR Somatic OR Psychotic OR Quality of life OR Well being OR Functioning** [**OR Mental health OR Dropouts)**](https://www.proquest.com/recentsearches.recentsearchtabview.recentsearchesgridview.scrolledrecentsearchlist.checkdbssearchlink:rerunsearch/E4041DCFE5A440DCPQ/None?t:ac=RecentSearches)

1. **CINAHL**

S1:

AB refugee* OR AB asylum seeker* OR AB migrant* OR AB immigrant* OR AB displaced

S2:

AB psychotherapy OR AB psychological intervention OR AB psychosocial OR AB intervention OR AB program* OR AB support

S3:

AB random* OR AB control* OR AB trial

S4:

mental OR disorder* OR distress OR trauma* OR depress* OR anxiety OR anxious OR post traumatic stress OR somatic OR psychotic OR functioning OR well being OR mental health OR dropouts

**S5:**

**S1 AND S2 AND S3 AND S4**

1. **SCOPUS**

#1

refugee* OR asylum seeker* OR migrant* OR immigrant* OR displaced

#2

psychotherapy OR psychological intervention OR psychosocial OR intervention OR program* OR support

#3

random* OR control* OR trial

#4

mental OR disorder* OR distress OR trauma* OR depress* OR anxiety OR anxious OR post traumatic stress OR somatic OR psychotic OR functioning OR well being OR quality of life OR mental health OR dropouts

**#5**

**1 AND 2 AND 3 AND 4**

1. **EMBASE**

#1 refugee* OR asylum seeker* OR migrant* OR immigrant* OR displaced

#2 psychotherapy OR psychological intervention OR psychosocial OR intervention OR programme OR support

#3 random* OR control* OR trial

#4 mental OR disorder* OR distress OR trauma* OR depress* OR anxiety OR anxious OR post traumatic stress OR somatic OR psychotic OR functioning OR well being OR quality of life OR mental health OR dropouts

#5 1 AND 2 AND 3 AND 4

**Table S1: References of the included studies identified by the search strategy**

| **REFERENCE** | | |
| --- | --- | --- |
| **Author** | **Year** | **Study full reference (Cambridge A style)** |
| **Acarturk 2015** | 2015 | **Acarturk C, Konuk E, Cetinkaya M, Senay I, Sijbrandij M, Cuijpers P and Aker T** (2015) EMDR for Syrian refugees with posttraumatic stress disorder symptoms: Results of a pilot randomized controlled trial. *European Journal of Psychotraumatology* **6**(1), 27414. |
| **Acarturk 2016** | 2016 | **Acarturk C, Konuk E, Cetinkaya M, Senay I, Sijbrandij M, Gulen B and Cuijpers P** (2016) The efficacy of eye movement desensitization and reprocessing for post–traumatic stress disorder and depression among Syrian refugees: Results of a randomized controlled trial. *Psychological medicine* **46**(12), 2583–2593. |
| **Acarturk 2022** | 2022 | **Acarturk C, Uygun E, Ilkkursun Z, Carswell K, Tedeschi F, Batu M, Eskici S, Kurt G, Anttila M, Au T, Baumgartner J, Churchill R, Cuijpers P, Becker T, Koesters M, Lantta T, Nosè M, Ostuzzi G, Popa M, Purgato M, Sijbrandij M, Turrini G, Välimäki M, Walker L, Wancata J, Zanini E, White RG, van Ommeren M and Barbui C** (2022) Effectiveness of a WHO self‐help psychological intervention for preventing mental disorders among Syrian refugees in Turkey: a randomized controlled trial. *World Psychiatry* **21**(1), 88–95. |
| **Acarturk 2022a** | 2022 | **Acarturk C, Uygun E, Ilkkursun Z, Yurtbakan T, Kurt G, Adam–Troian J, Senay I, Bryant R, Cuijpers P, Kiselev N, McDaid D, Morina N, Nisanci Z, Park AL, Sijbrandij M, Ventevogel P and Fuhr DC** (2022) Group problem management plus (PM+) to decrease psychological distress among Syrian refugees in Turkey: a pilot randomised controlled trial. *BMC psychiatry* **22**(1), 1–11. |
| **Acarturk 2022b** | 2022 | **Acarturk C, Kurt G, Ilkkursun Z, Uygun E and Karaoglan–Kahilogullari A** (2022) “Doing What Matters in Times of Stress” to Decrease Psychological Distress During COVID–19: A Randomised Controlled Pilot Trial. *Intervention* **20**(2), 170. |
| **Adenauer 2011** | 2011 | **Adenauer H, Catani C, Gola H, Keil J, Ruf M, Schauer M and Neuner F** (2011) Narrative exposure therapy for PTSD increases top-down processing of aversive stimuli - evidence from a randomized controlled treatment trial. BMC Neuroscience 12, 127. https://doi.org/10.1186/1471-2202-12-127. |
| **Aizik–Reebs 2021** | 2021 | **Aizik–Reebs A, Yuval K, Hadash Y, Gebreyohans Gebremariam S and Bernstein A** (2021) Mindfulness–based trauma recovery for refugees (MBTR–R): Randomized waitlist–control evidence of efficacy and safety. *Clinical Psychological Science* **9**(6), 1164–1184. |
| **Akhtar 2021** | 2021 | **Akhtar A, Malik A, Ghatasheh M, Aqel IS, Habashneh R, Dawson KS, Sarah Watts S, Jordans MJD, Brown F, Sijbrandij M, Cuijpers P and Bryant R** (2021) Feasibility trial of a brief scalable psychological intervention for Syrian refugee adolescents in Jordan. *European Journal of Psychotraumatology* **12**(1), 1901408. |
| **Akhtar 2021a** | 2021 | **Akhtar A, Giardinelli L, Bawaneh A, Awwad M, Al–Hayek H, Whitney C, Jordans MJD, Sijbrandij M, Cuijpers P, Dawson K and Bryant R** (2021) Feasibility trial of a scalable transdiagnostic group psychological intervention for Syrians residing in a refugee cAMP. *European Journal of Psychotraumatology* **12**(1), 1932295. |
| **Alegria 2019** | 2019 | **Alegría M, Falgas–Bague I, Collazos F, Camacho RC, Markle SL, Wang Y, Baca-García E, Lê Cook B, Chavez LM, Fortuna L, Herrera L, Qureshi A, Ramos Z, González C, Aroca P, García LA, Cellerino L, Villar A, Ali N, Mueser KT and Shrout PE** (2019) Evaluation of the integrated intervention for dual problems and early action among latino immigrants with co–occurring mental health and substance misuse symptoms: a randomized clinical trial. *JAMA network open* **2**(1), e186927–e186927. |
| **Ali 2020** | 2020 | **Ali ASASA** (2020) Efficiency of intervention counseling program on the enhanced psychological well–being and reduced post–traumatic stress disorder symptoms among Syrian women refugee survivors. *Clinical Practice and Epidemiology in Mental Health* **16**(Suppl–1), 134. |
| **Alsmadi 2018** | 2018 | **Alsmadi AM, Tawalbeh LI, Gammoh OS, Shawagfeh MQ, Zalloum W, Ashour and Attarian H** (2018) The effect of Ginkgo biloba and psycho–education on stress, anxiety and fatigue among refugees. *Proceedings of Singapore Healthcare* **27**(1), 26–32. |
| **Annan 2017** | 2017 | **Annan J, Sim A, Puffer ES, Salhi, C and Betancourt TS** (2017) Improving mental health outcomes of Burmese migrant and displaced children in Thailand: A community–based randomized controlled trial of a parenting and family skills intervention. *Prevention Science* **18**(7), 793–803. |
| **Baker 2006** | 2006 | **Baker F and Jones C** (2006) The effect of music therapy services on classroom behaviours of newly arrived refugee students in Australia—a pilot study. *Emotional and Behavioural Difficulties* **11**(4), 249–260. |
| **Banoglu 2022** | 2022 | **Banoğlu K and Korkmazlar** Ü (2022) Efficacy of the eye movement desensitization and reprocessing group protocol with children in reducing posttraumatic stress disorder in refugee children. *European Journal of Trauma and Dissociation* **6**(1), 100241. |
| **Beck 2021** | 2021 | **Beck BD, Meyer SL, Simonsen E, Søgaard U, Petersen I, Arnfred SMH, Tellier T and Moe T** (2021) Music therapy was noninferior to verbal standard treatment of traumatized refugees in mental health care: Results from a randomized clinical trial. *European Journal of Psychotraumatology* **12**(1), 1930960. |
| **Begotaraj 2022** | 2022 | **Begotaraj E, Sambucini D, Ciacchella C, Pellicano GR, Pierro L, Wamser–Nanney R, Aceto P, De Paola C, Caroppo E and Lai C** (2022) Effectiveness of the expressive writing on the psychological distress and traumatic symptoms of the migrants: A prospective study multiarm randomized controlled trial. *Psychological Trauma: Theory, Research, Practice and Policy* **15**(5), 738–747. |
| **Betancourt 2020** | 2020 | **Betancourt TS, Berent JM, Freeman J, Frounfelker RL, Brennan RT, Abdi S, Maalim A, Abdi A, Mishra T, Gautam B, Creswell JW and Beardslee WR** (2020) Family–based mental health promotion for Somali Bantu and Bhutanese refugees: Feasibility and acceptability trial. *Journal of Adolescent Health* **66**(3), 336–344. |
| **Bjorknes 2015** | 2015 | **Bjørknes R, Larsen M, Gwanzura–Ottemöller F and Kjøbli J** (2015) Exploring mental distress among immigrant mothers participating in parent training. *Children and Youth Services Review* **51**, 10–17. |
| **Blignault 2021** | 2021 | **Blignault I, Saab H, Woodland L and O’Callaghan C** (2021) Cultivating mindfulness: Evaluation of a community–based mindfulness program for Arabic–speaking women in Australia. *Current Psychology* **42**, 8232–8243. |
| **Böge 2022** | 2022 | **Böge K, Karnouk C, Hoell A, Tschorn M, Kamp–Becker I, Padberg F, Ubleis A, Hasan A, Falkai P, Salize H-J, Meyer-Lindenberg A, Banaschewski T, Schneider F, Habel U, Plener P, Hahn E, Wiechers M, Strupf M, Jobst A, Millenet S, Hoehne E, Sukale T, Dinauer R, Schuster M, Mehran N, Kaiser F, Bröcheler S, Lieb K, Heinz A, Rapp M and Bajbouj M** (2022) Effectiveness and cost–effectiveness for the treatment of depressive symptoms in refugees and asylum seekers: A multi–centred randomized controlled trial. *The Lancet Regional Health–Europe* **19**, 100413. |
| **Bolton 2007** | 2007 | **Bolton P, Bass J, Betancourt T, Speelman L, Onyango G, Clougherty KF, Neugebauer R, Murray L and Verdeli,H** (2007) Interventions for depression symptoms among adolescent survivors of war and displacement in northern Uganda: a randomized controlled trial. *Jama* **298**(5), 519–527. |
| **Bolton 2014** | 2014 | **Bolton P, Lee C, Haroz EE, Murray L, Dorsey S, Robinson C, Ugueto AM and Bass J** (2014) A transdiagnostic community–based mental health treatment for comorbid disorders: development and outcomes of a randomized controlled trial among Burmese refugees in Thailand. *PLoS medicine* **11**(11), e1001757. |
| **Brady 2021** | 2021 | **Brady F, Chisholm A, Walsh E, Ottisova L, Bevilacqua L, Mason C, von Werthern M, Cannon T, Curry C, Komolafe K, Robert RE, Robjant K and Katona C** (2021) Narrative exposure therapy for survivors of human trafficking: feasibility randomised controlled trial. *BJPsych Open* **7**(6), e196. |
| **Bryant 2022** | 2022 | **Bryant RA, Bawaneh A, Awwad M, Al–Hayek H, Giardinelli L, Whitney C, Jordans MJD, Cuijpers P, Sijbrandij M, Ventevogel P, Dawson K, Akhtar A and on behalf of the STRENGTHS Consortium** (2022) Effectiveness of a brief group behavioral intervention for common mental disorders in Syrian refugees in Jordan: A randomized controlled trial. *PLoS medicine* **19**(3), e1003949. |
| **Buhmann 2016** | 2016 | **Buhmann CB, Nordentoft M, Ekstroem M, Carlsson J, Mortensen EL** (2016) The effect of flexible cognitive–behavioural therapy and medical treatment, including antidepressants on post–traumatic stress disorder and depression in traumatised refugees: pragmatic randomised controlled clinical trial. *British Journal of Psychiatry* **208**(3):252–9. doi: 10.1192/bjp.bp.114.150961. |
| **Carlsson 2018** | 2018 | **Carlsson J, Sonne C, Vindbjerg E and Mortensen EL** (2018) Stress management versus cognitive restructuring in trauma–affected refugees—A pragmatic randomised study. *Psychiatry research* **266**, 116–123. |
| **Catani 2009** | 2009 | **Catani C, Kohiladevy M, Ruf M, Schauer E, Elbert T and Neuner F** (2009) Treating children traumatized by war and Tsunami: a comparison between exposure therapy and meditation–relaxation in North–East Sri Lanka. *BMC psychiatry* **9**(1), 1–11. |
| **Choi 2012** | 2012 | **Choi I, Zou J, Titov N, Dear BF, Li S, Johnston L, Andrews G and Hunt C** (2012) Culturally attuned Internet treatment for depression amongst Chinese Australians: a randomised controlled trial. *Journal of affective disorders* **136**(3), 459–468. |
| **Chung 2018** | 2018 | **Chung SK** (2018) The Effects of Using Expressive Intervention in Group Counseling on Acculturative Stress and Depressive Symptoms among Korean Immigrant Women in South and Central Texas: an experimental study. PhD dissertation, St. Mary's University, San Antonio, Texas. |
| **Cowell 2009** | 2009 | **Cowell JM, McNaughton D, Ailey S, Gross D and Fogg L** (2009) Clinical trail outcomes of the mexican american problem solving program (MAPS). *Hispanic health care international: the official journal of the National Association of Hispanic Nurses* **7**(4), 179. |
| **Cuijpers 2022** | 2022 | **Cuijpers P, Heim E, Abi Ramia J, Burchert S, Carswell K, Cornelisz I, Knaevelsrud C, Noun P, van Klaveren C, van’t Hof E, Zoghbi E, van Ommeren M and El Chammay R** (2022) Effects of a WHO–guided digital health intervention for depression in Syrian refugees in Lebanon: A randomized controlled trial. *PLoS Medicine* **19**(6), e1004025. |
| **de Graaf 2020** | 2020 | **De Graaff AM, Cuijpers P, McDaid D, Park A, Woodward A, Bryant RA, Fuhr DC, Kieft B, Minkenberg E, Sijbrandij M and on behalf of the STRENGTHS consortium** (2020) Peer–provided Problem Management Plus (PM+) for adult Syrian refugees: a pilot randomised controlled trial on effectiveness and cost–effectiveness. *Epidemiology and Psychiatric Sciences* **29**, 1–24. |
| **de Graaf 2023** | 2023 | **De Graaff AM, Cuijpers P, Twisk JW, Kieft B, Hunaidy S, Elsawy M, Gorgis N,Bouman TK, Lommen MJJ, Acarturk C, Bryant R, Burchert S, Dawson KS, Fuhr DC, Hansen P, Jordans m, Knaevelsrud C, McDaid D, Morina N, Moergeli H, Park A-l, Roberts B, Ventevogel P, Wiedemann N, Woodward A, Sijbrandij M on behalf of the STRENGTHS Consortium** (2023) Peer–provided psychological intervention for Syrian refugees: results of a randomised controlled trial on the effectiveness of Problem Management Plus. *BMJ Ment Health* **26**(1), 1–10. |
| **Dowrick 2022** | 2022 | **Dowrick C, Rosala–Hallas A, Rawlinson R, Khan N, Winrow E, Chiumento A, Burnside G, Aslam R, Billows L, Eriksson-Lee M, Lawrence D, McCluskey R, Mackinnon A, Moitt T, Orton L, Roberts E, Rahman A, Smith G, Edwards RT, Uwamaliya P and White R** (2022) The Problem Management Plus psychosocial intervention for distressed and functionally impaired asylum seekers and refugees: the PROSPER feasibility RCT. *Public Health Research* **10**(10). |
| **Dybdahl 2001** | 2001 | **Dybdahl R** (2001) Children and mothers in war: an outcome study of a psychosocial intervention program. *Child development* **72**(4), 1214–1230. |
| **El–Khani 2021** | 2021 | **El–Khani A, Cartwright K, Maalouf W, Haar K, Zehra N, Çokamay–Yılmaz G and Calam R** (2021) Enhancing Teaching Recovery Techniques (TRT) with parenting skills: RCT of TRT+ parenting with trauma–affected Syrian Refugees in Lebanon utilising remote training with implications for insecure contexts and COVID–19. *International Journal of Environmental Research and Public Health* **18**(16), 8652. |
| **Ell 2017** | 2017 | **Ell K, Aranda MP, Wu S, Oh H, Lee PJ and Guterman J** (2017) Promotora assisted depression and self–care management among predominantly Latinos with concurrent chronic illness: safety net care system clinical trial results. *Contemporary clinical trials* **61**, 1–9. |
| **Ertl 2011** | 2011 | **Ertl V, Pfeiffer A, Schauer E, Elbert T and Neuner F** (2011) Community–implemented trauma therapy for former child soldiers in Northern Uganda: a randomized controlled trial. *Jama* **306**(5), 503–512. |
| **Eruyar 2018** | 2018 | **Eruyar S** (2018) The role of parental factors in the mental health problems experienced by refugee children. PhD dissertation, Department of Neuroscience, Psychology and Behaviour, University of Leicester, United Kingdom. |
| **Escobar 1996** | 1996 | **Escobar SE** (1996) The effects of bicultural effectiveness training on psychological symptomatology in Central American and Mexican immigrant women. PhD dissertation, California School of Professional Psychology, San Diego, California. |
| **Eskici 2021** | 2021 | **Eskici HS, Hinton DE, Jalal B, Yurtbakan T and Acarturk C** (2021) Culturally adapted cognitive behavioral therapy for Syrian refugee women in Turkey: A randomized controlled trial. *Psychological trauma: theory, research, practice and policy* **15**(2), 189–198. |
| **Fine 2021** | 2021 | **Fine SL, Malik A, Guimond MF, Nemiro A, Temu, G, Likindikoki, S, Annan J and Tol WA** (2021) Improving mental health in low–resource settings: A feasibility randomized controlled trial of a transdiagnostic psychological intervention among Burundian refugee adolescents and their caregivers. *Behaviour Research and Therapy* **145**, 103944. |
| **Getanda 2020** | 2020 | **Getanda EM and Vostanis P** (2020) Feasibility evaluation of psychosocial intervention for internally displaced youth in Kenya. *Journal of Mental Health* 6, 774–782. |
| **Gever 2023** | 2023 | **Gever VC, Iyendo TO, Obiugo–Muoh UO, Okunade JK, Agujiobi–Odoh N, Udengwu N, Talabi FO and Nwokolo PN** (2023) Comparing the effect of social media–based drama, music and art therapies on reduction in post–traumatic symptoms among Nigerian refugees of Russia's invasion of Ukraine. *Journal of Pediatric* Nursing **68**, e96–e102. |
| **Goodkind 2020** | 2020 | **Goodkind JR, Amer S, Christian C, Hess JM, Bybee D, Isakson BL, Baca B and Shantzek C** (2017) Challenges and innovations in a community–based participatory randomized controlled trial. *Health Education and Behavior* **44**(1), 123–130. |
| **Greene 2021** | 2021 | **Greene MC, Likindikoki S, Rees S, Bonz A, Kaysen D, Misinzo L, Njau T, Kiluwa S, Turner R, Ventevogel P, Mbwambo JKK and Tol WA** (2021) Evaluation of an integrated intervention to reduce psychological distress and intimate partner violence in refugees: Results from the Nguvu cluster randomized feasibility trial. *PLoS one* **16**(6), e0252982. |
| **Gul 2018** | 2018 | **Gul R** (2017) Prevalence and Treatment of Post Traumatic Stress Disorder among Internally Displaced and Undisplaced Children. PhD dissertation, University of Peshawar, Peshawar, Pakistan. |
| **Gutierrez 2006** | 2006 | **Gutierrez SR** (2006) A comparison of two parent education programs on the reduction of parent–child stress in mother–child relationships of Hispanic migrant farmworkers. PhD dissertation, The University of Arizona, USA. |
| **Hasha 2022** | 2022 | **Hasha W, Igland J, Fadnes LT, Kumar BN, Heltne UM and Diaz E** (2022) Effect of a self–help group intervention using Teaching Recovery Techniques to improve mental health among Syrian refugees in Norway: a randomized controlled trial. *International Journal of Mental Health Systems* **16**(1), 47. |
| **Hensel–Dittmann 2011** | 2011 | **Hensel–Dittmann D, Schauer M, Ruf M, Catani C, Odenwald M, Elbert T and Neuner F** (2011) Treatment of traumatized victims of war and torture: a randomized controlled comparison of narrative exposure therapy and stress inoculation training. *Psychotherapy and psychosomatics* **80**(6), 345–352. https://doi.org/10.1159/000327253. |
| **Hijazi 2014** | 2014 | **Hijazi AM, Lumley MA, Ziadni MS, Haddad L, Rapport LJ and Arnetz BB** (2014) Brief narrative exposure therapy for posttraumatic stress in Iraqi refugees: a preliminary randomized clinical trial. *Journal of traumatic stress* **27**(3), 314–322. |
| **Hilado 2018** | 2018 | **Hilado A, Leow LC and Yang Y** (2018) *The Baby TALK–RefugeeOne Study: A randomized controlled trial examining home visiting services with refugees and immigrants*. Report submitted to the U.S. Department of Health and Human Services, Home Visiting Evidence of Effectiveness (HomVEE) Review. |
| **Hinton 2004** | 2004 | **Hinton DE, Pham T, Tran M, Safren SA, Otto MW and Pollack MH** (2004) CBT for Vietnamese refugees with treatment–resistant PTSD and panic attacks: a pilot study. *Journal of traumatic stress* **17**(5), 429–433. https://doi.org/10.1023/B:JOTS.0000048956.03529.fa. |
| **Hinton 2005** | 2005 | **Hinton DE, Chhean D, Pich V, Safren SA, Hofmann SG and Pollack MH** (2005) A randomized controlled trial of cognitive–behavior therapy for Cambodian refugees with treatment–resistant PTSD and panic attacks: a cross–over design. *Journal of traumatic stress* **18**(6), 617–629. https://doi.org/10.1002/jts.20070. |
| **Hinton 2009** | 2009 | **Hinton DE, Hofmann SG, Pollack MH and Otto MW** (2009) Mechanisms of efficacy of CBT for Cambodian refugees with PTSD: Improvement in emotion regulation and orthostatic blood pressure response. *CNS neuroscience and Psychological Trauma: Theory, Research, Practice and Policy* **15**(3), 255–263. |
| **Holzel 2016** | 2016 | **Hölzel LP, Ries Z, Kriston L, Dirmaier J, Zill JM, Rummel–Kluge C, Niebling W, Bermejo I and Härter M** (2016) Effects of culture–sensitive adaptation of patient information material on usefulness in migrants: a multicentre, blinded randomised controlled trial. *BMJ open* **6**(11), e012008. |
| **Hu J 2022** | 2022 | **Hu J, Liu IKF, Stewart SM, Lam TH and Yu NX** (2022) The More the Better, Only in the Longer Term: A Cluster Randomized Controlled Trial to Evaluate a Compound Intervention Among Mainland Chinese Immigrants in Hong Kong. *Behavior Therapy* **53**, 944–957. |
| **Ince 2013** | 2013 | **Ince BÜ, Cuijpers P, van't Hof E, van Ballegooijen W, Christensen H and Riper H** (2013) Internet–based, culturally sensitive, problem–solving therapy for Turkish migrants with depression: randomized controlled trial. *Journal of Medical Internet Research* **15**(10), e2853. |
| **Islam 2022** | 2022 | **Islam A, Mozumder TA, Rahman T, Shatil T and Siddique A** (2021) Forced Displacement, Mental Health and Child Development: Evidence from the Rohingya Refugees. SocArXiv preprint. https://doi:10.31235/osf.io/b4fc7. |
| **Kalantari 2012** | 2012 | **Kalantari M, Yule W, Dyregrov A, Neshatdoost H and Ahmadi SJ** (2012) Efficacy of writing for recovery on traumatic grief symptoms of Afghani refugee bereaved adolescents: a randomized control trial. *Omega* **65**(2), 139–150. https://doi.org/10.2190/OM.65.2.d. |
| **Kananian 2020** | 2020 | **Kananian S, Soltani Y, Hinton D and Stangier U** (2020) Culturally adapted cognitive behavioral therapy plus problem management (CA‐CBT+) with Afghan refugees: A randomized controlled pilot study. *Journal of traumatic stress* **33**(6), 928–938. |
| **Karasz 2015** | 2015 | **Karasz A, Raghavan S, Patel V, Zaman M, Akhter L and Kabita M** (2015) ASHA: using participatory methods to develop an Asset–building mental health intervention for Bangladeshi immigrant women. Progress in Community Health Partnerships: *Research, Education and Action* **9**(4), 501–512. |
| **Khedari dePierro 2020** | 2020 | **Khedari VK** (2020) Out of Ivory Towers and into Refugee Camps: Providing Refugees with Accessible Resources About the Biological and Psychological Effects of Forced Migration. PhD dissertation, The New School for Social Research of The New School University, New York. |
| **Kim 2015** | 2015 | **Kim HJ and Kim JM** (2015) Cognitive behavioral therapy for improving parenting efficacy of married immigrant women. *Journal of Families and Better* *Life* **33**(2), 135–148. |
| **Kiropuoulus 2011** | 2011 | **Kiropoulos LA, Griffiths KM and Blashki G** (2011) Effects of a multilingual information website intervention on the levels of depression literacy and depression–related stigma in Greek–born and Italian–born immigrants living in Australia: a randomized controlled trial. *Journal of medical Internet research* **13**(2), e1527. |
| **Knefel 2022** | 2022 | **Knefel M, Kantor V, Weindl D, Schiess–Jokanovic J, Nicholson AA, Verginer L, Schäfer I and Lueger–Schuster B** (2022) A brief transdiagnostic psychological intervention for Afghan asylum seekers and refugees in Austria: a randomized controlled trial. *European Journal of Psychotraumatology* **13**(1), 2068911. |
| **Ko 2022** | 2022 | **Ko HJ and Youn CH** (2011) Effects of laughter therapy on depression, cognition and sleep among the community‐dwelling elderly. *Geriatrics and* *gerontology international* **11**(3), 267–274. |
| **Koch 2020** | 2020 | **Koch T, Ehring T and Liedl A** (2020) Effectiveness of a transdiagnostic group intervention to enhance emotion regulation in young Afghan refugees: A pilot randomized controlled study. *Behaviour Research and Therapy* **132**, 103689. |
| **Kocken 2008** | 2008 | **Kocken PL, Joosten–van Zwanenburg E and de Hoop T** (2008) Effects of health education for migrant females with psychosomatic complaints treated by general practitioners: a randomised controlled evaluation study. *Patient Education and Counseling* **70**(1), 25–30. |
| **Kwong 2013** | 2013 | **Kwong K, Chung H, Cheal K, Chou JC and Chen T** (2013) Depression care management for Chinese Americans in primary care: a feasibility pilot study. *Community Mental Health Journal* **49**(2), 157–165. |
| **Lai 2020** | 2020 | **Lai DW, Li J, Ou X and Li CY** (2020) Effectiveness of a peer–based intervention on loneliness and social isolation of older Chinese immigrants in Canada: a randomized controlled trial. *BMC geriatrics* **20**(1), 1–12. |
| **Lau 2011** | 2011 | **Lau AS, Fung JJ, Ho LY, Liu LL and Gudiño OG** (2011) Parent training with high–risk immigrant Chinese families: A pilot group randomized trial yielding practice–based evidence. *Behavior Therapy* **42**(3), 413–426. |
| **Le 2011** | 2011 | **Le HN, Perry DF and Stuart EA** (2011) Randomized controlled trial of a preventive intervention for perinatal depression in high–risk Latinas. *Journal of consulting and clinical psychology* **79**(2), 135. |
| **Liedl 2011** | 2011 | **Liedl A, Müller J, Morina N, Karl A, Denke C and Knaevelsrud C** (2011) Retracted: physical activity within a CBT intervention improves coping with pain in traumatized refugees: results of a randomized controlled design. *Pain Medicine* **12**(2), 234–245. |
| **Lindegaard 2021** | 2020 | **Lindegaard T, Seaton F, Halaj A, Berg M, Kashoush F, Barchini R, Ludvigsson M, Sarkohi A and Andersson G** (2021) Internet–based cognitive behavioural therapy for depression and anxiety among Arabic–speaking individuals in Sweden: a pilot randomized controlled trial. *Cognitive Behaviour Therapy* **50**(1), 47–66. |
| **Lopez 1998** | 1998 | **Lopez NN** (1998) Multicultural effectiveness training for Hispanic immigrants. PhD dissertation, University of Miami, Florida. |
| **Lopez–Zéron 2019** | 2019 | **López‐Zerón G, Parra‐Cardona JR and Yeh HH** (2020) Addressing immigration‐related stress in a culturally adapted parenting intervention for Mexican‐origin immigrants: initial positive effects and key areas of improvement. *Family process* **59**(3), 1094–1112. |
| **Meffert 2014** | 2014 | **Meffert SM, Abdo AO, Alla OAA, Elmakki YOM, Omer AA, Yousif S, Metzler T and Marmar CR** (2014) A pilot randomized controlled trial of interpersonal psychotherapy for Sudanese refugees in Cairo, Egypt. *Psychological Trauma: Theory, Research, Practice and Policy* **6**(3), 240. |
| **Metzler 2022** | 2022 | **Metzler J, Saw T, Nono D, Kadondi A, Zhang Y, Leu CS, Gabriel A, Savage K and Landers C** (2023) Improving adolescent mental health and protection in humanitarian settings: longitudinal findings from a multi‐arm randomized controlled trial of child‐friendly spaces among South Sudanese refugees in Uganda. *Journal* *of child psychology and psychiatry* **64**(6), 907–917. |
| **Mhaidat 2016** | 2016 | **Mhaidat F and ALharbi BH** (2016) the impact of correcting cognitive distortions in reducing depression and the sense of insecurity among a sample of female refugee adolescents. *Contemporary Issues in Education Research (CIER)* **9**(4), 159–166. |
| **Miller 2020** | 2020 | **Miller KE, Koppenol–Gonzalez GV, Arnous M, Tossyeh F, Chen A, Nahas N and Jordans MJ** (2020) Supporting Syrian families displaced by armed conflict: a pilot randomized controlled trial of the Caregiver Support Intervention. *Child Abuse and Neglect* **106**, 104512. |
| **Miller 2023** | 2023 | **Miller KE, Chen A, Koppenol‐Gonzalez GV, Bakolis I, Arnous M, Tossyeh F, El Hassan A, Saleh A, Saade J, Nahas N, Abboud M, Jawad L and Jordans MJ** (2023) Supporting parenting among Syrian refugees in Lebanon: a randomized controlled trial of the caregiver support intervention. *Journal of Child Psychology and Psychiatry* **64**(1), 71–82. |
| **Morath 2014** | 2014 | **Morath J, Gola H, Sommershof A, Hamuni G, Kolassa S, Catani C, Adenauer H, Ruf-Leuschner M, Schauer M, Elbert T, Groettrup M and Kolassa IT** (2014) The effect of trauma–focused therapy on the altered T cell distribution in individuals with PTSD: evidence from a randomized controlled trial. *Journal of Psychiatric Research* **54**, 1–10. |
| **Motaghed 1990** | 1990 | **Motaghed H** (1990) The efficacy of a group–oriented cognitive treatment program in treating immigrant Persians for depression. PhD dissertation, California School of Professional Psychology, San Diego, California. |
| **Neuner 2004** | 2004 | **Neuner F, Schauer M, Klaschik C, Karunakara U and Elbert T** (2004) A comparison of narrative exposure therapy, supportive counseling and psychoeducation for treating posttraumatic stress disorder in an african refugee settlement. *Journal of consulting and clinical psychology* **72**(4), 579. |
| **Neuner 2008** | 2008 | **Neuner F, Onyut PL, Ertl V, Odenwald M, Schauer E and Elbert T** (2008) Treatment of posttraumatic stress disorder by trained lay counselors in an African refugee settlement: a randomized controlled trial. *Journal of consulting and clinical psychology* **76**(4), 686. |
| **Neuner 2010** | 2010 | **Neuner F, Kurreck S, Ruf M, Odenwald M, Elbert T and Schauer M** (2010) Can asylum–seekers with posttraumatic stress disorder be successfully treated? A randomized controlled pilot study. *Cognitive behaviour therapy* **39**(2), 81–91. |
| **Nickel 2006** | 2006 | **Nickel M, Cangoez B, Bachler E, Muehlbacher M, Lojewski N, Mueller–Rabe N, Mitterlehner FO, Egger C, Leiberich P, Rother N, Buschmann W, Kettler C, Pedrosa Gil F, Lahmann C, Fartacek R, Wolfhardt K. Rother WK, Loew TH and Nickel C** (2006) Bioenergetic exercises in inpatient treatment of Turkish immigrants with chronic somatoform disorders: a randomized, controlled study. *Journal of psychosomatic research* **61**(4), 507–513. |
| **Nickerson 2020** | 2020 | **Nickerson A, Byrow Y, Pajak R, McMahon T, Bryant RA, Christensen H and Liddell BJ** (2020) ‘Tell Your Story’: a randomized controlled trial of an online intervention to reduce mental health stigma and increase help–seeking in refugee men with posttraumatic stress. *Psychological Medicine* **50**(5), 781–792. |
| **Nnanyelugo 2022** | 2022 | **Nnanyelugo CE, Iyendo TO, Emmanuel NO, Okwuowulu C, Izuchukwu John E, Apuke OD and Gever VC** (2022) Effect of Internet–mediated music therapy intervention on reduction in generalized anxiety disorder symptoms among displaced Nigerians of the Russia–Ukraine war. *Psychology of Music* 51(4), 1149–1159. |
| **Nordbrandt 2020** | 2020 | **Nordbrandt MS, Sonne C, Mortensen EL and Carlsson J** (2020) Trauma–affected refugees treated with basic body awareness therapy or mixed physical activity as augmentation to treatment as usual—A pragmatic randomised controlled trial. *PloS one* **15**(3), e0230300. |
| **Northwood 2020** | 2020 | **Northwood AK, Vukovich MM, Beckman A, Walter JP, Josiah N, Hudak L, O’Donnell Burrows C, Letts JP and Danner CC** (2020) Intensive psychotherapy and case management for Karen refugees with major depression in primary care: a pragmatic randomized control trial. *BMC Family Practice* **21**(1), 1–13. |
| **Nygren 2019** | 2019 | **Nygren T, Brohede D, Koshnaw K, Osman SS, Johansson R and Andersson G** (2019) Internet‐ based treatment of depressive symptoms in a Kurdish population: A randomized controlled trial. *Journal of Clinical Psychology* **75**(6), 985–998. https://doi.org/10.1002/jclp.22753. |
| **Ooi 2016** | 2016 | **Ooi CS, Rooney RM, Roberts C, Kane RT, Wright B and Chatzisarantis N** (2016) The efficacy of a group cognitive behavioral therapy for war–affected young migrants living in Australia: a cluster randomized controlled trial. *Frontiers in Psychology* **7**, 1641. |
| **Orang 2022** | 2022 | **Orang TM, Missmahl I, Thoele AM, Valensise L, Brenner A, Gardisi M, Peter H and Kluge U** (2022) New directions in the mental health care of migrants, including refugees—A randomized controlled trial investigating the efficacy of value‐based counselling. *Clinical Psychology and Psychotherapy* 29(4), 1433–1446. |
| **Osman 2017** | 2017 | **Osman F, Salari R, Klingberg–Allvin M, Schön UK and Flacking R** (2017) Effects of a culturally tailored parenting support programme in Somali–born parents’ mental health and sense of competence in parenting: a randomised controlled trial. BMJ open, **7**(12), e017600. |
| **Otto 2003** | 2003 | **Otto MW, Hinton D, Korbly NB, Chea A, Ba P, Gershuny BS and Pollack MH** (2003) Treatment of pharmacotherapy–refractory posttraumatic stress disorder among Cambodian refugees: a pilot study of combination treatment with cognitive–behavior therapy vs sertraline alone. *Behaviour research* *and therapy* **41**(11), 1271–1276. |
| **Paunovic 2001** | 2001 | **Paunovic N and Öst LG** (2001) Cognitive–behavior therapy vs exposure therapy in the treatment of PTSD in refugees. *Behaviour research and therapy* **39**(10), 1183–1197. |
| **Pfeiffer 2018** | 2018 | **Pfeiffer E, Sachser C, Rohlmann F and Goldbeck L** (2018) Effectiveness of a trauma‐focused group intervention for young refugees: A randomized controlled trial. *Journal of Child Psychology and Psychiatry* **59**(11), 1171–1179. |
| **Purgato 2021** | 2021 | **Purgato M, Carswell K, Tedeschi F, Acarturk C, Anttila M, Au T, Bajbouj M, Baumgartner J, Biondi M, Churchill R, Cuijpers P, Koesters M, Gastaldon C, Ilkkursun Z, Lantta T, Nosè M, Ostuzzi G, Papola D, Popa M, Roselli V, Sijbrandij m, Tarsitani L, Turrini G, Välimäki M, Walker L, Wancata J, Zanini E, White R, van Ommeren M and Barbui C** (2021) Effectiveness of self–help plus in preventing mental disorders in refugees and asylum seekers in Western Europe: a multinational randomized controlled trial. *Psychotherapy and Psychosomatics* **90**(6), 403–414. |
| **Renner 2011** | 2011 | **Renner W, Bänninger–Huber E and Peltzer K** (2011) Culture–sensitive and Resource Oriented Peer (CROP) groups as a community based intervention for trauma survivors: A randomized controlled study with refugees and asylum seekers from Chechnya. *Australasian Journal of Disaster and Trauma Studies* **1**, 1–14. |
| **Renner 2011a** | 2011 | **Renner W and Berry JW** (2011) The ineffectiveness of group interventions for female Turkish migrants with recurrent depression. *Social Behavior and* *Personality: an international journal* **39**(9), 1217–1234. |
| **Renner 2012** | 2012 | **Renner W, Laireiter AR and Maier MJ** (2012) Social support as a moderator of acculturative stress among refugees and asylum seekers. *Social Behavior and Personality: an international journal* **40**(1), 129–145. |
| **Röhr 2021** | 2021 | **Röhr S, Jung FU, Pabst A, Grochtdrei T, Dams J, Nagl M, Renner A, Hoffmann R, König H-H, Kersting A and Riedel–Heller SG** (2021) A self–help app for Syrian refugees with posttraumatic stress (Sanadak): randomized controlled trial. *JMIR mHealth and uHealth* **9**(1), e24807. |
| **Rondung 2022** | 2022 | **Rondung E, Leiler A, Sarkadi A, Bjärtå A, Lampa E, Löfving SG, Calam R, Oppedal B, Keeshin B and Warner G** (2022) Feasibility of a randomised trial of Teaching Recovery Techniques (TRT) with refugee youth: results from a pilot of the Swedish UnaccomPanied yOuth Refugee Trial (SUPpORT). *Pilot and Feasibility Studies* **8**(1), 1–15. |
| **Ruf 2010** | 2010 | **Ruf M, Schauer M, Neuner F, Catani C, Schauer E and Elbert T** (2010) Narrative exposure therapy for 7‐to 16‐year‐olds: A randomized controlled trial with traumatized refugee children. *Journal of traumatic stress* **23**(4), 437–445. |
| **Saito 2012** | 2012 | **Saito T, Kai I and Takizawa A** (2012) Effects of a program to prevent social isolation on loneliness, depression and subjective well–being of older adults: a randomized trial among older migrants in Japan. *Archives of gerontology and geriatrics* **55**(3), 539–547. |
| **Sandahl 2021** | 2021 | **Sandahl H, Jennum P, Baandrup L, Lykke Mortensen E and Carlsson J** (2021) Imagery rehearsal therapy and/or mianserin in treatment of refugees diagnosed with PTSD: Results from a randomized controlled trial. *Journal of sleep research* **30**(4), e13276. |
| **Schauer 2006** | 2006 | **Schauer M, Elbert T, Gotthardt S, Rockstroh B, Odenwald M and Neuner F** (2006) Wiedererfahrung durch Psychotherapie modifiziert Geist und Gehirn. *Verhaltenstherapie* **16**(2), 96–103. |
| **Schottelkorb 2012** | 2012 | **Schottelkorb AA, Doumas DM and Garcia R** (2012) Treatment for childhood refugee trauma: A randomized, controlled trial. *International Journal of Play Therapy* **21**(2), 57. |
| **Shaw 2019** | 2019 | **Shaw SA, Ward KP, Pillai V and Hinton DE** (2019) A group mental health randomized controlled trial for female refugees in Malaysia. *American Journal of Orthopsychiatry* **89**(6), 665–674. |
| **Shaw 2020** | 2020 | **Shaw SA, Ward KP, Pillai V, Ali LM and Karim H** (2021) A randomized clinical trial testing a parenting intervention among Afghan and Rohingya refugees in Malaysia*. Family process* **60**(3), 788–805. |
| **She 2022** | 2022 | **She X, Tong L, Wang H, Lan L, Wang M, Abbey C, Singh M and Rozelle S** (2022) Planting Seeds for Resilience—a Pilot Mindfulness Program in Migrant Chinese Children. *Pediatrics* **149**(1 Meeting Abstracts February 2022), 504. |
| **Shovaz 2022** | 2022 | **Shovaz FA, Zareei Mahmoodabadi H and Salehzadeh M** (2022) Effectiveness of life skills training based on self–care on mental health and quality of life of married Afghan women in Iran. *BMC Women's Health* **22**(1), 296. |
| **Sirin 2018** | 2018 | **Sirin S, Plass JL, Homer BD, Vatanartiran S and Tsai T** (2018) Digital game–based education for Syrian refugee children: Project Hope. *Vulnerable* *Children and Youth Studies* **13**(1), 7–18. |
| **Sleptsova 2013** | 2013 | **Sleptsova M, Wössmer B, Grossman P and Langewitz WA** (2013) Culturally sensitive group therapy for Turkish patients suffering from chronic pain: a randomised controlled intervention trial. *Swiss medical weekly* **143**, w13875. |
| **Smokowski 2009** | 2009 | **Smokowski PR and Bacallao M** (2009) Entre dos mundos/between two worlds youth violence prevention: Comparing psychodramatic and support group delivery formats. *Small Group Research* **40**(1), 3–27. |
| **Spaaij 2022** | 2022 | **Spaaij J, Kiselev N, Berger C, Bryant RA, Cuijpers P, De Graaff AM, Fuhr DC, Hemmo M, McDaid D, Moergeli H, Park A-L, Pfaltz MC, Schick M, Schnyder U, Wenger A, Sijbrandij M and Morina N on behalf of the STRENGTHS Consortium** (2022) Feasibility and acceptability of Problem Management Plus (PM+) among Syrian refugees and asylum seekers in Switzerland: a mixed–method pilot randomized controlled trial. *European journal of psychotraumatology* **13**(1), 2002027. |
| **Spanhel 2022** | 2022 | **Spanhel K, Hovestadt E, Lehr D, Spiegelhalder K, Baumeister H, Bengel J and Sander LB** (2022) Engaging Refugees With a Culturally Adapted Digital Intervention to Improve Sleep: A Randomized Controlled Pilot Trial. *Frontiers in psychiatry* **13**, 832196. |
| **Stenmark 2013** | 2013 | **Stenmark H, Catani C, Neuner F, Elbert T and Holen A** (2013) Treating PTSD in refugees and asylum seekers within the general health care system. A randomized controlled multicenter study. *Behaviour research and therapy* 51(10), 641–647. https://doi.org/10.1016/j.brat.2013.07.002. |
| **Tam 2019** | 2019 | **Tam CC, Li X, Benotsch EG and Lin D** (2020) A resilience‐based intervention programme to enhance psychological well‐being and protective factors for rural‐to‐urban migrant children in China. *Applied Psychology: Health and Well‐Being* **12**(1), 53–76. |
| **Tay 2020** | 2020 | **Tay AK, Mung HK, Miah MAA, Balasundaram S, Ventevogel P, Badrudduza M, Khan S, Morgan K, Rees S, Mohsin M and Silove D** (2020) An Integrative Adapt Therapy for common mental health symptoms and adaptive stress amongst Rohingya, Chin and Kachin refugees living in Malaysia: A randomized controlled trial. *PLoS medicine* **17**(3), e1003073. |
| **ter Heide 2011** | 2011 | **Ter Heide FJJ, Mooren T, Kleijn W, de Jongh A and Kleber R** (2011) EMDR versus stabilisation in traumatised asylum seekers and refugees: Results of a pilot study. *European Journal of Psychotraumatology* **2**(1), 5881. |
| **ter Heide 2016** | 2016 | **Ter Heide FJJ, Mooren TM, Van de Schoot R, De Jongh A and Kleber RJ** (2016) Eye movement desensitisation and reprocessing therapy v. stabilisation as usual for refugees: randomised controlled trial. *The British Journal of Psychiatry* **209**(4), 311–318. |
| **Tiwari 2020** | 2020 | **Tiwari A, Fong DYT, Yuen FKH, Fung HY, Pang POY and Wong JYH** (2020) Purpose–built intervention for mental health of Mainland Chinese immigrant women survivors of intimate partner violence: a randomised controlled trial (abridged secondary publication). *Medical Journal* **26**(6), 7. |
| **Tol 2020** | 2020 | **Tol WA, Leku MR, Lakin DP, Carswell K, Augustinavicius J, Adaku A, Au TM, Brown FL, Bryant RA, Garcia-Moreno C, Musci RJ, Ventevogel P, White RG and van Ommeren M** (2020) Guided self–help to reduce psychological distress in South Sudanese female refugees in Uganda: a cluster randomised trial. *The Lancet Global Health* **8**(2), e254–e263. |
| **Vahabi 2022** | 2022 | **Vahabi M, Wong JPH, Moosapoor M, Akbarian A and Fung K** (2022) Effects of Acceptance and Commitment Therapy (ACT) on Mental Health and Resiliency of Migrant Live–in Caregivers in Canada: Pilot Randomized Wait List Controlled Trial. *JMIR formative research* **6**(1), e32136. |
| **Vijayakumar 2017** | 2017 | **Vijayakumar L, Mohanraj R, Kumar S, Jeyaseelan V, Sriram S and Shanmugam M** (2017) CASP–An intervention by community volunteers to reduce suicidal behaviour among refugees. *International Journal of Social Psychiatry* **63**(7), 589–597. |
| **Walg 2020** | 2020 | **Walg M, Angern JS, Michalak J and Hapfelmeier G** (2020) Effectiveness of stabilization training for adolescent refugees with trauma–induced disorders: A randomized controlled trial. *Zeitschrift fur Kinder–und Jugendpsychiatrie und Psychotherapie* **48**(5), 369–379. |
| **Weine 2008** | 2008 | **Weine S, Kulauzovic Y, Klebic A, Besic S, Mujagic A, Muzurovic J, Spahovic D, Stanley Sclove S and Rolland J** (2008) Evaluating a multiple‐family group access intervention for refugees with PTSD. *Journal of marital and family therapy* **34**(2), 149–164. |
| **Weinstein 2016** | 2016 | **Weinstein N, Farah K and Nicole L** (2016) Enhancing need satisfaction to reduce psychological distress in Syrian refugees. *Journal of Consulting and Clinical Psychology* **84**(7), 645–650. |
| **White–Baugan 1990** | 1990 | **White–Baughan JL** (1990) The effects of a problem–solving intervention with educational videos on symptoms of posttraumatic stress in a sample of Cambodian refugees. PhD dissertation, California School of Professional Psychology, San Diego, California. |
| **Yeung 2016** | 2016 | **Yeung A, Martinson MA, Baer L, Chen J, Clain A, Williams A, Chang TE, Trinh N-HT, Alpert JE and Fava M** (2016) The effectiveness of telepsychiatry–based culturally sensitive collaborative treatment for depressed Chinese American immigrants: a randomized controlled trial. *The Journal of Clinical Psychiatry* **77**(8), 20755. |
| **Yurtsever 2018** | 2018 | **Yurtsever A, Konuk E, Akyüz T, Zat Z, Tükel F, Çetinkaya M, Savran C and Shapiro E** (2018) An eye movement desensitization and reprocessing (EMDR) group intervention for Syrian refugees with post–traumatic stress symptoms: Results of a randomized controlled trial. *Frontiers in psychology* **9**(493), 1–8. |

**Table S2: List of excluded studies with reasons**

| **Study ID** | **Full Reference** | **Reason for exclusion** |
| --- | --- | --- |
| (Acarturk et al. 2021) | **Acarturk C, Ostuzzi G, Purgato M, Tedeschi F and Barbui, C**. (2021) Self-Help Plus (SH+) for preventing mental disorders in refugees and asylum seekers: The RE-DEFINE Turkish randomized trial. | duplicate |
| (ACTRN12610000426088, 2010) | **ACTRN12610000426088** (2010) Randomised Controlled Trial of Cognitive Behavior Therapy and Supportive Counselling for Reduction in Posttraumatic Stress Disorder (PTSD) Symptoms in Refugees. *Https://Trialsearch.Who.Int/Trial2.Aspx?TrialID=ACTRN12610000426088*. | ongoing |
| (ACTRN12611000948998, 2011) | **ACTRN12611000948998** (2011) The efficacy and social validity of a group cognitive behavioural therapy for young migrants from war–affected countries. Https://Trialsearch.Who.Int/Trial2.Aspx?TrialID=ACTRN12611000948998. | duplicate |
| (ACTRN12616000860460, 2016) | **ACTRN12616000860460** (2016) Efficacy of the Triple P Parenting Intervention for Migrant and Former–Refugee Parents: Randomized Controlled Trial. Https://Trialsearch.Who.Int/Trial2.Aspx?TrialID=ACTRN12616000860460. | ongoing |
| (ACTRN12617001452381, 2017) | **ACTRN12617001452381** (2017) Randomised Controlled Trial of Integrated Adapt Therapy vs Problem Management Plus to strengthen resilience and adaptive capacity and reduce posttraumatic comorbidities in refugees exposed to war–related traumatic events and adversity. Https://Trialsearch.Who.Int/Trial2.Aspx?TrialID=ACTRN12617001452381. | duplicate |
| (ACTRN12618001664235, 2018) | **ACTRN12618001664235** (2018) Evaluating an EEG neurofeedback intervention for posttraumatic stress disorder in adult refugees. Https://Trialsearch.Who.Int/Trial2.Aspx?TrialID=ACTRN12618001664235. | wrong study design |
| (ACTRN12618001845224, 2018) | **ACTRN12618001845224** (2018) HARMONY – A randomised controlled trial of a GP systems intervention for family violence experienced by South Asian migrant and refugee women and children in Melbourne, Australia. https://Trialsearch.Who.Int/Trial2.Aspx?TrialID=ACTRN12618001845224. | wrong outcome |
| (ACTRN12618001917224, 2018) | **ACTRN12618001917224** (2018) Pilot Study of Testing Group Psychological Help for Young Adolescent Syrian Refugees in Jordan. Https://Trialsearch.Who.Int/Trial2.Aspx?TrialID=ACTRN12618001917224. | duplicate |
| (ACTRN12619000168156, 2019) | **ACTRN12619000168156** (2019) Pilot Study of Testing Group Psychological Help for Syrian Refugees in Jordan. Https://Trialsearch.Who.Int/Trial2.Aspx?TrialID=ACTRN12619000168156. | duplicate |
| (ACTRN12619000340134, 2019) | **ACTRN12619000340134** (2019) Pilot Study of Testing Group Psychological Help for Adult Syrian Refugees in Jordan. Https://Trialsearch.Who.Int/Trial2.Aspx?TrialID=ACTRN12619000340134. | duplicate |
| (ACTRN12619000341123, 2019) | **ACTRN12619000341123** (2019) Testing Group Psychological Help for Young Adolescent Syrian Refugees in Jordan. Https://Trialsearch.Who.Int/Trial2.Aspx?TrialID=ACTRN12619000341123. | duplicate |
| (ACTRN12619000381189, 2019) | **ACTRN12619000381189** (2019) Psychological Treatment of Posttraumatic Stress Disorder in Refugees. *Https://Trialsearch.Who.Int/Trial2.Aspx?TrialID=ACTRN12619000381189*. | ongoing |
| (ACTRN12621000189820, 2021) | **ACTRN12621000189820** (2021) Effect of a Stepped Care Intervention on Anxiety and Depression In Distressed People in Jordan. *Https://Trialsearch.Who.Int/Trial2.Aspx?TrialID=ACTRN12621000189820*. | ongoing |
| (ACTRN12621001160820, 2021) | **ACTRN12621001160820** (2021) MEmory Training for Recovery– Adolescent (METRA): A brief intervention targeting psychological distress in adolescent refugees. *Https://Trialsearch.Who.Int/Trial2.Aspx?TrialID=ACTRN12621001160820*. | ongoing |
| (ACTRN12621001731886, 2021) | **ACTRN12621001731886** (2021) Tell Your Story: Can an Online Intervention Reduce Stigma and Increase Help–Seeking in Refugee Men and Women? Https://Trialsearch.Who.Int/Trial2.Aspx?TrialID=ACTRN12621001731886. | wrong outcome |
| (ACTRN12622001048774, 2022) | **ACTRN12622001048774** (2022) Psychological Treatment of Posttraumatic Stress Disorder and Moral Injury in Refugees. Https://Trialsearch.Who.Int/Trial2.Aspx?TrialID=ACTRN12622001048774. | ongoing |
| (Ager et al. 2011) | **Ager A, Akesson B, Stark L, Flouri E, Okot B, McCollister F and Boothby N** (2011) The Impact of the School–Based Psychosocial Structured Activities (PSSA) Program on Conflict–Affected Children in Northern Uganda. *Journal of Child Psychology and Psychiatry* **52**(11), 1124–1133. | wrong study design |
| (Ahn and Ji–Hyun, 2022) | **Ahn J-Y and Ji–Hyun S** (2022) The Effects of an Online Group Art Therapy Program on the Meaning of Life and Psychological Well–Being of Japanese Middle–Aged Married Migrant Women. *Korean Journal of Art Therapy* **29**(1), 21–43. | no full text |
| (Aizik–Reebs et al. 2022) | **Aizik–Reebs A, Amir I, Yuval K, Hadash Y and Bernstein A** (2022) Candidate mechanisms of action of mindfulness–based trauma recovery for refugees (MBTR–R): Self–compassion and self–criticism. *Journal of Consulting and Clinical Psychology* **90**(2), 107–122. https://doi.org/10.1037/ccp0000716. | duplicate |
| (Akhtar et al. 2020) | **Akhtar A, Giardinelli L, Bawaneh A, Awwad M, Naser H, Whitney C, Jordans MJD, Sijbrandij M and Bryant RA** (2020) Group problem management plus (gPM+) in the treatment of common mental disorders in Syrian refugees in a Jordanian camp: Study protocol for a randomized controlled trial. *BMC Public Health* **20**(1), 390. https://doi.org/10.1186/s12889–020–08463–5. | duplicate |
| (Al Azdi et al. 2021) | **Al Azdi Z, Islam K, Muhammad AK, Khan N, Ejaz A, Muhammad AK, Warraitch A, Jahan I and Huque R** (2021) Effectiveness of an Integrated Care Package for Refugee Mothers and Children: Protocol for a Cluster Randomized Controlled Trial. *JMIR Research Protocols* **10**(5). | ongoing |
| (Alegria et al. 2019) | **Alegria M, Frontera W, Cruz–Gonzalez M, Markle SL, Trinh–Shevrin C, Wang Y, Herrera L, Ishikawa RZ, Velazquez E, Fuentes L, Guo Y, Pan J, Cheung M, Wong J, Genatios U, Jimenez A, Ramos Z, Perez G, Wong JY,Chieng C-K and Shrout PE** (2019) Effectiveness of a Disability Preventive Intervention for Minority and Immigrant Elders: The Positive Minds–Strong Bodies Randomized Clinical Trial. *The American Journal of Geriatric Psychiatry: Official Journal of the American Association for Geriatric Psychiatry* **27**(12), 1299–1313. | wrong population |
| (Alozkan Sever et al. 2021) | **Alozkan Sever C, Cuijpers P, Mittendorfer–Rutz E, Bryant RA, Dawson KS, Holmes EA, Mooren T, Norredam ML and Sijbrandij M** (2021) Feasibility and acceptability of Problem Management Plus with Emotional Processing (PM+EP) for refugee youth living in the Netherlands: Study protocol. *European Journal of Psychotraumatology* **12**(1), 1947003. | ongoing |
| (Andersen et al. 2012) | **Andersen E, Burton NW and Anderssen SA** (2012) Physical activity levels six months after a randomised controlled physical activity intervention for Pakistani immigrant men living in Norway. *International Journal of Behavioral Nutrition and Physical Activity* **9**(1), 1–10. | wrong intervention |
| (Arntz et al. 2013) | **Arntz A, Sofi D and van Breukelen G** (2013) Imagery Rescripting as treatment for complicated PTSD in refugees: A multiple baseline case series study. *Behaviour Research and Therapy* **51**(6), 274–283. https://doi.org/10.1016/j.brat.2013.02.009. | wrong study design |
| (Arola et al. 2020) | **Arola A, Dahlin–Ivanoff S and Häggblom–Kronlöf G** (2020) Impact of a person–centred group intervention on life satisfaction and engagement in activities among persons aging in the context of migration. *Scandinavian Journal of Occupational Therapy* **27**(4), 269–279. | wrong population |
| (Barhoma et al. 2021) | **Barhoma M, Sonne C, Lommen MJJ, Mortensen EL and Carlsson J** (2021) Stress management versus cognitive restructuring in trauma–affected refugees—A follow–up study on a pragmatic randomised trial. *Journal of Affective Disorders* **294**, 628–637. | wrong study design |
| (Barrett et al. 2001) | **Barrett P, Sonderegger R and Sonderegger N** (2001) Evaluation of an anxiety–prevention and positive–coping program (FRIENDS) for children and adolescents of non–English–speaking background. *Behaviour Change* **18**(2), 78–91. | wrong study design |
| (Barrett et al. 2003) | **Barrett PM, Sonderegger R, Xenos S and Barrett B** (2003) Using FRIENDS to combat anxiety and adjustment problems among young migrants to Australia: A national trial. *Clinical Child Psychology and Psychiatry* **8**(2), 241–260. | wrong study design |
| (Becerra and Ulibarri, 2019) | **Becerra RA and Ulibarri M** (2019) Intimate Partner Violence Risk Recognition, Danger Assessment, Mental Health Symptoms and Acculturation Among U.S. and Foreign–born Mexican Survivors of Intimate Partner Violence. PhD dissertation, Alliant International University, Alhambra, California. https://www.proquest.com/dissertations–theses/intimate–partner–violence–risk–recognition–danger/docview/2239956370/se–2?accountid=14836. | wrong intervention |
| (Beck et al. 2018) | **Beck BD, Lund ST, Sogaard U, Simonsen E, Tellier TC, Cordtz TO, Laier GH and Moe T** (2018) Music therapy versus treatment as usual for refugees diagnosed with posttraumatic stress disorder (PTSD): Study protocol for a randomized controlled trial. *Trials* **19**(1), 301. | duplicate |
| (Benyakar et al. 1994) | **Benyakar M, Kretsch R and Baruch E** (1994) Mental health work with Gulf War evacuees: The use of a transitional therapeutic space. *Israel Journal of Psychiatry and Related Sciences* **31**(2), 78–85. | wrong study design |
| (Betancourt et al. 2012) | **Betancourt TS, Newnham EA, Brennan RT, Verdeli H, Borisova I, Neugebauer R, Bass J and Bolton P** (2012) Moderators of treatment effectiveness for war–affected youth with depression in Northern Uganda. *Journal of Adolescent Health* **51**(6), 544–550. https://doi.org/10.1016/j.jadohealth.2012.02.010. | duplicate |
| (Bjørknes and Manger, 2013) | **Bjørknes R and Manger T** (2013) Can parent training alter parent practice and reduce conduct problems in ethnic minority children? A randomized controlled trial. *Prevention Science* **14**(1), 52–63. | duplicate |
| (Boettcher J. et al. 2021) | **Boettcher J, Wirz C, Paskuy S, Bottche M, Renneberg B and Wagner B** (2021) Experiences with tailoring treatment modules in online versus face–to–face CBT. *European Psychiatry* **64**, S19. https://doi.org/10.1192/j.eurpsy.2021.74. | ongoing |
| (Bogdanov et al. 2021) | **Bogdanov S, Augustinavicius J, Bass JK, Metz K, Skavenski S, Singh NS, Moore Q, Haroz EE, Kane J, Doty B, Murray L and Bolton P** (2021) A randomized–controlled trial of community–based transdiagnostic psychotherapy for veterans and internally displaced persons in Ukraine. *Global Mental Health* **8**. | wrong population |
| (Boge et al. 2020) | **Boge K, Karnouk C, Hahn E, Schneider F, Habel U, Banaschewski T, Meyer–Lindenberg A, Salize HJ, Kamp–Becker I, Padberg F, Hasan A, Falkai P, Rapp MA, Plener PL, Stamm T, Elnahrawy N, Lieb K, Heinz A and Bajbouj M** (2020) Mental health in refugees and asylum seekers (MEHIRA): Study design and methodology of a prospective multicentre randomized controlled trail investigating the effects of a stepped and collaborative care model. *European Archives of Psychiatry and Clinical Neuroscience* **270**(1), 95–106. | duplicate |
| (Bradley et al. 2006) | **Bradley GM, Couchman GM, Perlesz A, Nguyen AT, Singh B and Riess C** (2006) Multiple–family group treatment for English– and Vietnamese–speaking families living with schizophrenia. *Psychiatric Services* **57**(4), 521–530. | wrong population |
| (Brown et al. 2018) | **Brown FL, Carswell K, Augustinavicius J, Adaku A, Leku MR, White RG, Ventevogel P, Kogan CS, García–Moreno C, Bryant RA, Musci RJ, van Ommeren M and Tol WA** (2018) Self Help Plus: Study protocol for a cluster–randomised controlled trial of guided self–help with South Sudanese refugee women in Uganda. *Global Mental Health* **5**, e27. | duplicate |
| (Brown et al. 2022) | **Brown FL, Bosqui T, Elias J, Farah S, Mayya A, Abo Nakkoul D, Walsh B, Chreif S, Einein A, Meksassi B, Abi Saad R, Naal H, Ghossainy ME, Donnelly M, Betancourt TS, Carr A, Puffer E, El Chammay R and Jordans MJD** (2022) Family systemic psychosocial support for at–risk adolescents in Lebanon: Study protocol for a multi–site randomised controlled trial. *Trials* **23**(1), 327. https://doi.org/10.1186/s13063–022–06284–y. | ongoing |
| (Bruhn et al. 2022) | **Bruhn M, Laugesen H, Kromann–Larsen M, Trevino CS, Eplov L, Hjorthoj C and Carlsson J** (2022) The effect of an integrated care intervention of multidisciplinary mental health treatment and employment services for trauma–affected refugees: Study protocol for a randomised controlled trial. *Trials* **23**(1) https://doi.org/10.1186/s13063–022–06774–z. | duplicate |
| (Buhmann C.B. et al. 2018) | **Buhmann CB, Nordentoft M, Ekstroem M, Carlsson J and Mortensen EL** (2018) Long–term treatment effect of trauma–affected refugees with flexible cognitive behavioural therapy and antidepressants. *Psychiatry Research* **264**, 217–223. https://doi.org/10.1016/j.psychres.2018.03.069. | duplicate |
| (Buhmann, 2014) | **Buhmann CB** (2014) Traumatized refugees: Morbidity, treatment and predictors of outcome. *Danish Medical Journal* **61**(8), B4871. | wrong study design |
| (Byrskog et al. 2019) | **Byrskog U, Ahrne M, Small R, Andersson E, Essen B, Adan A, Ahmed FH, Tesser K, Liden Y, Israelsson M, Ahman–Berndtsson A and Schytt E** (2019) Rationale, development and feasibility of group antenatal care for immigrant women in Sweden: A study protocol for the Hooyo Project. *BMJ Open* **9**(7), e030314. | wrong study design |
| (Callister and Brown, 2020) | **Callister LC and Brown T** (2020) Self–help–plus: Making a difference for vulnerable women. *MCN: The American Journal of Maternal/Child Nursing* **45**(5), 311. | wrong study design |
| (Cavic and Pejovic, 2005) | **Cavic T and Pejovic M** (2005) Evaluation of group cognitive psychotherapy of post–traumatic stress disorder. *Psihijatrija Danas* **37**(2), 315–322. | wrong study design |
| (Çelik et al. 2022) | **Çelik SB, Özkan E and Bumin G** (2022) Effects of Occupational Therapy via Telerehabilitation on Occupational Balance, Well–Being, Intrinsic Motivation and Quality of Life in Syrian Refugee Children in COVID–19 Lockdown: A Randomized Controlled Trial. *Children* **9**(4), 485. | wrong intervention |
| (Changrani et al. 2008) | **Changrani J, Lieberman M, Golant M, Rios P, Damman J and Gany F** (2008) Online cancer support groups: Experiences with underserved immigrant latinas. *Primary Psychiatry* **15**(10), 55–62. | wrong outcome |
| (Chu Q. et al. 2019) | **Chu Q, Wong CCY and Lu Q** (2019) Acculturation Moderates the Effects of Expressive Writing on Post–Traumatic Stress Symptoms Among Chinese American Breast Cancer Survivors. *International Journal of Behavioral Medicine* **26**(2), 185–194. https://doi.org/10.1007/s12529–019–09769–4. | wrong intervention |
| (Clukay et al. 2018) | **Clukay CJ, Dajani R, Hamadmad D, Abudayyeh G, Panter–Brick C and Mulligan CJ** (2018) Genetics of risk and trauma exposure in Syrian refugee youth. *American Journal of Physical Anthropology* **165**(66), 51. | wrong study design |
| (Cohen et al. 2021) | **Cohen F, Hermosilla S, Knox J, Agaba GS, Obalim G, Kajungu R, Mangen PO and Stark L** (2021) Protocol for a caregiver psychosocial support intervention for populations affected by displacement in Uganda. *BMC Public Health* **21**(1), 932. | wrong study design |
| (Corbit et al. 2022) | **Corbit J, Didkowsky N, Gora V, Reddy H, Muhammad S and Callaghan T** (2022) Facilitating the prosocial development of Rohingya refugee children. Journal of Experimental Child Psychology, 220(Aime, H, Broesch, T, Aknin, L.B, Warneken, F (2017) Evidence for proactive and reactive helping in two–to five–year–olds from a small–scale society. *PLoS One* **12**(11), e187787, 1–18. https://doi.org/10.1016/j.jecp.2022.105414. | wrong study design |
| (Damra, 2022) | **Damra JK** (2022) The Effects of Psychodrama Intervention on Intimate Partner Violence and Quality of Life: Trial of Syrian Refugee Abused Women. *Journal of International Women’s Studies* **23**(1), 1–14. | wrong outcome |
| (de Graaff et al. 2020) | **de Graaff AM, Cuijpers P, Acarturk C, Bryant R, Burchert S, Fuhr DC, Huizink AC, de Jong J, Kieft B, Knaevelsrud C, McDaid D, Morina N, A–La P, Uppendahl J, Ventevogel P, Whitney C, Wiedemann N, Woodward A and Sijbrandij M** (2020) Effectiveness of a peer–refugee delivered psychological intervention to reduce psychological distress among adult Syrian refugees in the Netherlands: Study protocol. *European Journal of Psychotraumatology* **11**(1). | duplicate |
| (Denkinger et al. 2022) | **Denkinger JK, Rometsch C, Murray K, Schneck U, Brisslinger LK, Azad ZR, Windthorst P, Graf J, Hautzinger M, Zipfel S and Junne** **F** (2022) Addressing barriers to mental health services: Evaluation of a psychoeducational short film for forcibly displaced people. *European journal of psychotraumatology* **13**(1) https://doi.org/10.1080/20008198.2022.2066458. | wrong study design |
| (Devries et al. 2019) | **Devries KM, Fabbri C, Allen E, Barongo V, Shayo E, Greco G, Kaemingk M, Qiu M, Steinacher R, Tol W and Rodrigues** **K** (2019) Preventing violence against children in schools (PVACS): Protocol for a cluster randomised controlled trial of the EmpaTeach behavioural intervention in Nyarugusu refugee camp. *BMC Public Health* **19**(1), 1295. | ongoing |
| (Dozio and Bizouerne, 2021) | **Dozio E and Bizouerne C** (2021) Psychological rapid response to population movements in Democratic Republic of Congo (DRC). *European Psychiatry* **64**(Supplement 1), S321–S322. https://doi.org/10.1192/j.eurpsy.2021.863. | wrong study design |
| (DRKS00000090, 2009) | **DRKS00000090** (2009) Primary prevention of alcohol–related disorders in older migrants—Development and evaluation of a transcultural prevention concept. Https://Trialsearch.Who.Int/Trial2.Aspx?TrialID=DRKS00000090. | wrong outcome |
| (DRKS00016154, 2018) | **DRKS00016154** (2018) Culturally adapted CBT plus Problem–solving therapy with Afghan Refugees: A Randomized Controlled Trial. Https://Trialsearch.Who.Int/Trial2.Aspx?TrialID=DRKS00016154. | duplicate |
| (DRKS00016538, 2019) | **DRKS00016538** (2019) A brief psychological intervention on mental health among Treatment seeking for Afghan asylum seekers and refugees in Austria. Https://Trialsearch.Who.Int/Trial2.Aspx?TrialID=DRKS00016538. | duplicate |
| (DRKS00016867, 2019) | **DRKS00016867** (2019) Promoting the resilience of refugees and migrants—Efficacy study on the use of mother tongue counselor. Https://Trialsearch.Who.Int/Trial2.Aspx?TrialID=DRKS00016867. | duplicate |
| (DRKS00017668, 2021) | **DRKS00017668** (2021) A Randomized Controlled Trial of STARC („Skills Training in Affect Regulation—A Culture–sensitive approach”) in refugees with substance use problems. Https://Trialsearch.Who.Int/Trial2.Aspx?TrialID=DRKS00017668. | ongoing |
| (DRKS00017754, 2019) | **DRKS00017754** (2019) Metacognitive Training in Depression (D–MKT) for the Target Group of Asylum Seekers (Refugees): Feasibility Study (Proof of Concept) Https://Trialsearch.Who.Int/Trial2.Aspx?TrialID=DRKS00017754. | ongoing |
| (DRKS00017838, 2019) | **DRKS00017838** (2019) Feasibility, acceptance and effectiveness of Step–by–Step, a smartphone–based self–help program for Syrian refugees: A pilot study. Https://Trialsearch.Who.Int/Trial2.Aspx?TrialID=DRKS00017838. | ongoing |
| (DRKS00018949, 2020) | **DRKS00018949** (2020) Feasibility and efficacy of an online–training for an improved sleep of refugees: A randomised–controlled pilot study. Https://Trialsearch.Who.Int/Trial2.Aspx?TrialID=DRKS00018949. | duplicate |
| (DRKS00019072, 2020) | **DRKS00019072** (2020) Improve–MH: Improving mental health in refugee families with young children. Https://Trialsearch.Who.Int/Trial2.Aspx?TrialID=DRKS00019072. | ongoing |
| (DRKS00019876, 2020) | **DRKS00019876** (2020) Brief Imagery Rescripting vs. Treatment as Usual in Refugees with Posttraumatic Stress Disorder â€“ A Multi–Center Randomized Controlled Trial. Https://Trialsearch.Who.Int/Trial2.Aspx?TrialID=DRKS00019876. | duplicate |
| (DRKS00020564, 2020) | **DRKS00020564** (2020) Efficacy of a Low–threshold, Cultural Sensitive Group Psychoeducation in Asylum Seekers (LoPe): A multicenter–randomized controlled trial. Https://Trialsearch.Who.Int/Trial2.Aspx?TrialID=DRKS00020564. | duplicate |
| (DRKS00020771, 2020) | **DRKS00020771** (2020) A randomized, waiting list controlled, multi–center study to evaluate START (Stress–Trauma Symptoms–Arousal–Regulation–Treatment) training for young refugees with traumatic stress related disorders. Https://Trialsearch.Who.Int/Trial2.Aspx?TrialID=DRKS00020771. | duplicate |
| (DRKS00021536, 2020)9/6/23 2:05:00 PM | **DRKS00021536** (2020) Culturally Adapted Cognitive–Behavioral Group Therapy for Mental Disorders in Refugees plus Problem Management Training (CA–CBT+): A randomized controlled trial (ReTreat) Https://Trialsearch.Who.Int/Trial2.Aspx?TrialID=DRKS00021536. | ongoing |
| (DRKS00022143, 2020) | **DRKS00022143** (2020) Randomized controlled trial to test the (cost–)effectiveness of Step–by–Step, a smartphone–based self–help program for Syrian refugees in Germany. Https://Trialsearch.Who.Int/Trial2.Aspx?TrialID=DRKS00022143. | ongoing |
| (DRKS00022144, 2020) | **DRKS00022144** (2020) Randomized controlled trial to test the (cost–)effectiveness of Step–by–Step, a smartphone–based self–help program for Syrian refugees in Sweden. Https://Trialsearch.Who.Int/Trial2.Aspx?TrialID=DRKS00022144. | ongoing |
| (DRKS00022862, 2020) | **DRKS00022862** (2020) Effects of Stabilizing and Guided Imagery Techniques among traumatized refugees in collective or municipal accommodations: A randomized controlled trial. Https://Trialsearch.Who.Int/Trial2.Aspx?TrialID=DRKS00022862. | ongoing |
| (DRKS00023505, 2020) | **DRKS00023505** (2020) Randomized controlled trial to test the (cost–)effectiveness of Step–by–Step, a smartphone–based self–help program for Syrian refugees in Egypt. Https://Trialsearch.Who.Int/Trial2.Aspx?TrialID=DRKS00023505. | ongoing |
| (DRKS00024154, 2021) | **DRKS00024154** (2021) Effectiveness of a transdiagnostic intervention (CETA) in an internet–based versus face–to–face setting for Arabic–speaking refugees: A randomized control group study. Https://Trialsearch.Who.Int/Trial2.Aspx?TrialID=DRKS00024154. | ongoing |
| (DRKS00024419, 2021) | **DRKS00024419** (2021) Acceptance and efficacy of a transdiagnostic group–intervention for sleep disorders in male Afghan refugees: A pilot RCT. Https://Trialsearch.Who.Int/Trial2.Aspx?TrialID=DRKS00024419. | ongoing |
| (DRKS00024737, 2021) | **DRKS00024737** (2021) A real–time fMRI neurofeedback investigation on resilience in Arab refugees and migrants. Https://Trialsearch.Who.Int/Trial2.Aspx?TrialID=DRKS00024737. | ongoing |
| (DRKS00028061, 2022) | **DRKS00028061** (2022) Wisdom training to reduce stress and improve adjustment and psychological wellbeing in refugees. Https://Trialsearch.Who.Int/Trial2.Aspx?TrialID=DRKS00028061. | ongoing |
| (Drozdek and Bolwerk, 2010) | **Drozdek B and Bolwerk N** (2010) Evaluation of group therapy with traumatized asylum seekers and refugees—The Den Bosch model. *Traumatology* **16**(4), 117–127. | wrong study design |
| (Drozek et al. 2012) | **Drozek B, Kamperman AM, Bolwerk N, Tol WA and Kleber RJ** (2012) Group therapy with male asylum seekers and refugees with posttraumatic stress disorder: A controlled comparison cohort study of three day–treatment programs. *Journal of Nervous and Mental Disease* **200**(9), 758–765. https://doi.org/10.1097/NMD.0b013e318266f860. | wrong study design |
| (Durbeej et al. 2021) | **Durbeej N, McDiarmid S, Sarkadi A, Feldman I, Punamaki R–L, Kankaanpaa R, Andersen A, Hilden P, Verelst A, Derluyn I and Osman F** (2021) Evaluation of a school–based intervention to promote mental health of refugee youth in Sweden (The RefugeesWellSchool Trial): Study protocol for a cluster randomized controlled trial. *Trials* **22**(1), 98. https://doi.org/10.1186/s13063–020–04995–8. | duplicate |
| (Durbeej et al. 2021) | **Durbeej N, McDiarmid S, Sarkadi A, Feldman I, Punamaki R–L, Kankaanpaa R, Andersen A, Hilden PK, Verelst A, Derluyn I and Osman** **F** (2021) Evaluation of a school–based intervention to promote mental health of refugee youth in Sweden (The RefugeesWellSchool Trial): Study protocol for a cluster randomized controlled trial. *Trials* **22**(1), 98. | ongoing |
| (Ehntholt et al. 2005) | **Ehntholt KA, Smith PA and Yule W** (2005) School–based cognitive–behavioural therapy group intervention for refugee children who have experienced war–related trauma. *Clinical Child Psychology and Psychiatry* **10**(2), 235–250. | wrong study design |
| (Ekstrom et al. 2016) | **Ekstrom M, Carlsson J, Sonne C and Mortensen EL** (2016) Stress management versus cognitive restructuring: A randomized clinical study on traumatized refugees. *European Psychiatry* **33**, S399–S400. https://doi.org/10.1016/j.eurpsy.2016.01.1437. | duplicate |
| (EUCTR2008–006714–15–DK, 2009) | **EUCTR2008–006714–15–DK** (2009) The effect of treatment of traumatised refugees with Traume–Focused Cognitive Behavioural Therapy and antidepressants—A randomised controlled clinical trial. Https://Trialsearch.Who.Int/Trial2.Aspx?TrialID=EUCTR2008–006714–15–DK. | duplicate |
| (EUCTR2015–004153–40–DK, 2015) | **EUCTR2015–004153–40–DK** (2015) Treatment of sleep disturbances in trauma–affected refugees – a randomised controlled trial. Https://Trialsearch.Who.Int/Trial2.Aspx?TrialID=EUCTR2015–004153–40–DK. | duplicate |
| (Eylem et al. 2015) | **Eylem O, van Straten A, Bhui K and Kerkhof AJFM** (2015) Protocol: Reducing suicidal ideation among Turkish migrants in the Netherlands and in the UK: effectiveness of an online intervention. *International Review of Psychiatry* **27**(1), 72–81. | duplicate |
| (Eylem et al. 2021) | **Eylem O, van Straten A, de Wit L, Rathod S, Bhui K and Kerkhof AJFM** (2021) Reducing suicidal ideation among Turkish migrants in the Netherlands and in the UK: the feasibility of a randomised controlled trial of a guided online intervention. *Pilot and Feasibility Studies* **7**(1), 30. | awaiting assessment |
| (Falb et al. 2016) | **Falb KL, Tanner S, Ward L, Erksine D, Noble E, Assazenew A, Bakomere T, Graybill E, Lowry C, Mallinga P, Neiman A, Poulton C, Robinette K, Sommer M and Stark L** (2016) Creating opportunities through mentorship, parental involvement and safe spaces (COMPASS) program: Multi–country study protocol to protect girls from violence in humanitarian settings. *BMC Public Health* **16**, 231. https://doi.org/10.1186/s12889–016–2894–3. | wrong outcome |
| (Falgas–Bague et al. 2019) | **Falgas–Bague I, Wang Y, Banerjee S, Ali N, DiMarzio K, Palao Vidal D and Alegría M** (2019) Predictors of Adherence to Treatment in Behavioral Health Therapy for Latino Immigrants: The Importance of Trust. *Frontiers in Psychiatry* **10**, 817. | duplicate |
| (Feen–Calligan et al. 2020) | **Feen–Calligan H, Grasser LR, Debryn J, Nasser S, Jackson C, Seguin D and Javanbakht A** (2020) Art therapy with Syrian refugee youth in the United States: An intervention study. *Arts in psychotherapy* **69**. | wrong study design |
| (Foka et al. 2021) | **Foka S, Hadfield K, Pluess M and Mareschal I** (2021) Promoting well–being in refugee children: An exploratory controlled trial of a positive psychology intervention delivered in Greek refugee camps. *Development and Psychopathology* **33**(1), 87–95. | wrong study design |
| (Fortuna et al. 2020) | **Fortuna LR, Falgas–Bague I, Ramos Z, Porche MV and Alegria M** (2020) Development of a cognitive behavioral therapy with integrated mindfulness for Latinx immigrants with co–occurring disorders: Analysis of intermediary outcomes. *Psychological Trauma: Theory, Research, Practice and Policy* **12**(8), 825–835. https://doi.org/10.1037/tra0000949. | duplicate |
| (Fox et al. 1998) | **Fox P, Cowell J, Montgomery A and Willgerodt M** (1998) Southeast Asian refugee women and depression: A nursing intervention. *International Journal of Psychiatric Nursing Research* **4**(1), 423–432. | duplicate |
| (Fox et al. 1998) | **Fox P, Cowell J, Montgomery A and Willgerodt M** (1998) Southeast Asian refugee women and depression: A nursing intervention. *International Journal of Psychiatric Nursing Research* **4**(1), 423–432. | no full text |
| (Franco et al. 2020) | **Franco C, Soriano E, Amutio A and Manas I** (2020) Improving motivation in Latin American immigrants through a mindfulness-based program: A randomized study. *Terapia Psicologica* **38**(1), 5–16. https://doi.org/10.4067/S0718–48082020000100005. | no full text |
| (Friedman et al. 2021) | **Friedman S, Calderon B, Gonzalez A, Suruki C, Blanchard A, Cahill E, Kester K, Muna M, Elbel E, Purushothaman P, Krause MC and Meyer D** (2021) Pediatric Practice Redesign with Group Well Child Care Visits: A Multi–Site Study. *Maternal and Child Health Journal* **25**(8), 1265–1273. https://doi.org/10.1007/s10995–021–03146–y. | wrong study design |
| (Fung et al. 2021) | **Fung KP–L, Vahabi M, Moosapoor M, Akbarian A, Liu JJ-W and Wong JP–H** (2021) Implementation of an Internet–Based Acceptance and Commitment Therapy for Promoting Mental Health Among Migrant Live–in Caregivers in Canada: Protocol. *JMIR Research Protocols* **10**(9) https://www.proquest.com/scholarly–journals/implementation–internet–based–acceptance/docview/2577904065/se–2. | duplicate |
| (Galano et al. 2017) | **Galano MM, Grogan–Kaylor AC, Stein SF, Clark HM and Graham–Bermann SA** (2017) Posttraumatic stress disorder in Latina women: Examining the efficacy of the Moms’ Empowerment Program. *Psychological Trauma: Theory, Research, Practice and Policy* **9**(3), 344–351. | wrong study design |
| (Garcia et al. 2019) | **Garcia Y, Ferras C, Rocha A and Aguilera A** (2019) Exploratory Study of Psychosocial Therapies with Text Messages to Mobile Phones in Groups of Vulnerable Immigrant Women. *Journal of Medical Systems* **43**(8), 277. https://doi.org/10.1007/s10916–019–1393–3. | wrong study design |
| (Gerber et al. 2021) | **Gerber M, Colledge F, de Quervain D, Filippou K, Havas E, Knappe F, Ludyga S, Meier M, Morres ID, Panagos A, Puhse U, Ramadan K, Seelig H, Theodorakis Y, von Kanel R and Hatzigeorgiadis A** (2021) Effects of an exercise and sport intervention among refugees living in a Greek refugee camp on mental health, physical fitness and cardiovascular risk markers: Study protocol for the SALEEM pragmatic randomized controlled trial. *Trials* **22**(1), 827. | wrong intervention |
| (Golchert et al. 2019) | **Golchert J, Roehr S, Berg F, Grochtdreis T, Hoffmann R, Jung F, Nagl M, Plexnies A, Renner A, Konig H–H, Kersting A and Riedel–Heller SG** (2019) HELP@APP: development and evaluation of a self–help app for traumatized Syrian refugees in Germany—A study protocol of a randomized controlled trial. *BMC Psychiatry* **19**(1), 131. | duplicate |
| (Goodkind et al. 2017) | **Goodkind JR, Amer S, Christian C, Hess JM, Bybee D, Isakson BL, Baca B, Ndayisenga M, Greene RN and Shantzek C** (2017) Challenges and Innovations in a Community–Based Participatory Randomized Controlled Trial. *Health Education and Behavior: The Official Publication of the Society for Public Health Education* **44**(1), 123–130. | duplicate |
| (Gormez et al. 2017) | **Gormez V, Kilic H, Orengul A, Demir M, Mert E, Makhlouta B, Kinik K and Semerci B** (2017) Evaluation of a school–based, teacher–delivered psychological intervention group program for trauma–affected Syrian refugee children in Istanbul, Turkey. *Psychiatry and Clinical Psychopharmacology* **27**(2), 125–131. | wrong study design |
| (Green et al. 2016) | **Green EP, Blattman C, Jamison J and Annan J** (2016) Does poverty alleviation decrease depression symptoms in post–conflict settings? A cluster–randomized trial of microenterprise assistance in Northern Uganda. *Global Mental Health* **3**, 9. | wrong outcome |
| (Greene et al. 2022) | **Greene MC, Bonz A, Cristobal M, Vega C, Andersen LS, Angulo A, Armijos A, Guevara ME, Benavides L, de la Cruz A, Lopez MJ, Moyano A, Murcia A, Noboa MJ, Rodriguez A, Solis J, Vergara D, Scharf J, Dutt P, Wainberg M and Tol WA** (2022) Evaluating the feasibility of a group psychosocial intervention for migrant and host community women in Ecuador and Panama: Protocol for a multi–site feasibility cluster trial. *Pilot and feasibility studies* **8**(1) https://doi.org/10.1186/s40814–022–01085–1. | ongoing |
| (Gupta and Zimmer, 2008) | **Gupta L and Zimmer C** (2008) Psychosocial intervention for war–affected children in Sierra Leone. *The British Journal of Psychiatry: The Journal of Mental Science* **192**(3), 212–216. | wrong study design |
| (Gustafsson et al. 2017) | **Gustafsson S, Berglund H, Faronbi J, Barenfeld E and Ottenvall Hammar I** (2017) Minor positive effects of health–promoting senior meetings for older community–dwelling persons on loneliness, social network and social support. *Clinical Interventions in Aging* **12**, 1867–1877. | wrong study design |
| (Hasha et al. 2019) | **Hasha W, Fadnes LT, Igland J, Vardal R, Giusti LM, Stromme EM, Haj–Younes J, Heltne U, Kumar BN and Diaz E** (2019) Two interventions to treat pain disorders and post–traumatic symptoms among Syrian refugees: Protocol for a randomized controlled trial. *Trials* **20**(1), 784. https://doi.org/10.1186/s13063–019–3919–x. | duplicate |
| (Hasha et al. 2020) | **Hasha W, Igland J, Fadnes LT, Kumar B, Haj–Younes J, Strømme EM, Norstein EZ, Vårdal R and Diaz E** (2020) The Effect of Physiotherapy Group Intervention in Reducing Pain Disorders and Mental Health Symptoms among Syrian Refugees: A Randomized Controlled Trial. *International Journal of Environmental Research and Public Health* **17**(24). | wrong intervention |
| (Heim et al. 2021a) | **Heim E, Ramia JA, Hana RA, Burchert S, Carswell K, Cornelisz I, Cuijpers P, El Chammay R, Noun P, van Klaveren C, van Ommeren M, Zoghbi E and van’t Hof E** (2021a) Step–by–step: Feasibility randomised controlled trial of a mobile–based intervention for depression among populations affected by adversity in Lebanon. *Internet Interventions* **24**, 100380. https://doi.org/10.1016/j.invent.2021.100380. | wrong population |
| (Hernandez and Organista, 2013) | **Hernandez MY and Organista KC** (2013) Entertainment–Education? A Fotonovela? A New Strategy to Improve Depression Literacy and Help–Seeking Behaviors in At–Risk Immigrant Latinas. *American Journal of Community Psychology* **52**(3–4), 224–235. https://doi.org/10.1007/s10464–013–9587–1. | awaiting assessment |
| (Hijazi, 2012) | **Hijazi AM** (2012) Narrative Exposure Therapy to treat traumatic stress in Middle Eastern refugees: A clinical trial. PhD dissertation. Wayne State University, Detrit, Michigan. https://www.proquest.com/dissertations–theses/narrative–exposure–therapy–treat–traumatic–stress/docview/1222228003/se–2?accountid=14836. | wrong study design |
| (Hjellset et al. 2011) | **Hjellset VT, Ihlebaek CM, Bjorge B, Eriksen HR and Hostmark AT** (2011) Health–Related Quality of Life, Subjective Health Complaints, Psychological Distress and Coping in Pakistani Immigrant Women With and Without the Metabolic Syndrome: The InnvaDiab–DEPLAN Study on Pakistani Immigrant Women Living in Oslo, Norway. *Journal of Immigrant and Minority Health* **13**(4), 732–741. | wrong intervention |
| (Holzel et al. 2014) | **Holzel LP, Ries Z, Zill JM, Kriston L, Dirmaier J, Harter M and Bermejo I** (2014) Development and testing of culturally sensitive patient information material for Turkish, Polish, Russian and Italian migrants with depression or chronic low back pain (KULTINFO): Study protocol for a double–blind randomized controlled trial. *Trials* **15**(101263253), 265. | duplicate |
| (Hoover, 2019) | **Hoover SH** (2019) 43.1 strengthening transition resilience of newcomer groups (strong): pilot findings from a cognitive–behavioral intervention for refugees and immigrants in schools. *Journal of the American Academy of Child and Adolescent Psychiatry* **58**(10), S364–S365. https://doi.org/10.1016/j.jaac.2019.07.891. | wrong study design |
| (Hovey et al. 2014) | **Hovey JD, Hurtado G and Seligman LD** (2014) Findings for a CBT Support Group for Latina Migrant Farmworkers in Western Colorado. *Current psychology* **33**(3), 271–281. | wrong study design |
| (Hu, 2007) | **Hu X** (2007) Effects of Tai Chi on functional fitness and subjective health status in older Japanese returnees from China: A randomized controlled trial. *Japanese Journal Of Physical Fitness And Sports Medicine* **56**(4), 409–417. | wrong outcome |
| (In, 2020) | **In H** (2020) Changes in Self–Esteem and Subjective Wellbeing of Marriage Immigrant Women in Group Art Therapy. *Journal of Arts Psychothreapy* **16**(1), 161–188. | no full text |
| (ISRCTN10892553, 2019) | **ISRCTN10892553** (2019) Delivering a contextualized package of care for child development (0–12 months) and maternal mental health in the camps for forcibly displaced Myanmar nationals in Bangladesh. Https://Trialsearch.Who.Int/Trial2.Aspx?TrialID=ISRCTN10892553. | duplicate |
| (ISRCTN12942763, 2016) | **ISRCTN12942763** (2016) Assessing the impact of the I–Deal Life Skills Intervention on the psychosocial wellbeing of Syrian refugee children in Lebanon. Https://Trialsearch.Who.Int/Trial2.Aspx?TrialID=ISRCTN12942763. | duplicate |
| (ISRCTN14572069, 2019) | **ISRCTN14572069** (2019) A peer–based intervention to reduce social isolation of older Chinese immigrants in Canada. Https://Trialsearch.Who.Int/Trial2.Aspx?TrialID=ISRCTN14572069. | duplicate |
| (ISRCTN15214107, 2019) | **ISRCTN15214107** (2019) Pilot trial of an evidence–based low intensity psychosocial intervention delivered by lay therapists for asylum seekers and refugees. Https://Trialsearch.Who.Int/Trial2.Aspx?TrialID=ISRCTN15214107. | duplicate |
| (ISRCTN17754931, 2019) | **ISRCTN17754931** (2019) Accompanied refugeeS In Sweden Trial (ASsIST). Https://Trialsearch.Who.Int/Trial2.Aspx?TrialID=ISRCTN17754931. | duplicate |
| (ISRCTN20474555, 2019) | **ISRCTN20474555** (2019) Community–based Socio–therapy Adapted for Refugees: The COSTAR study. *Https://Trialsearch.Who.Int/Trial2.Aspx?TrialID=ISRCTN20474555*. | ongoing |
| (ISRCTN22321773, 2019) | **ISRCTN22321773** (2019) Helping refugee parents thrive: An evaluation of the caregiver support intervention with Syrian refugees in Lebanon. Https://Trialsearch.Who.Int/Trial2.Aspx?TrialID=ISRCTN22321773. | duplicate |
| (ISRCTN33665023, 2019) | **ISRCTN33665023** (2019) Helping refugee parents thrive: Assessing the readiness of the caregiver support intervention for a fully powered evaluation with Syrian refugees in Lebanon. Https://Trialsearch.Who.Int/Trial2.Aspx?TrialID=ISRCTN33665023. | duplicate |
| (ISRCTN47401844, 2018) | **ISRCTN47401844** (2018) Energy in Balance: An intervention aimed at migrant women with medically unexplained symptoms. Https://Trialsearch.Who.Int/Trial2.Aspx?TrialID=ISRCTN47401844. | ongoing |
| (ISRCTN47820795, 2018) | **ISRCTN47820795** (2018) Swedish unaccompanied youth refugee trial. Https://Trialsearch.Who.Int/Trial2.Aspx?TrialID=ISRCTN47820795. | duplicate |
| (ISRCTN48004304, 2007) | **ISRCTN48004304** (2007) Outcome study on the classroom/camp/community–based intervention for children exposed to armed conflict in Nepal. Https://Trialsearch.Who.Int/Trial2.Aspx?TrialID=ISRCTN48004304. | wrong population |
| (ISRCTN48178969, 2019) | **ISRCTN48178969** (2019) RefugeesWellSchool (Sweden): Teaching recovery techniques and in–service teacher training program. Https://Trialsearch.Who.Int/Trial2.Aspx?TrialID=ISRCTN48178969. | duplicate |
| (ISRCTN50148022, 2017) | **ISRCTN50148022** (2017) Self–help plus (SH+) for South Sudanese refugees in Uganda. Https://Trialsearch.Who.Int/Trial2.Aspx?TrialID=ISRCTN50148022. | duplicate |
| (ISRCTN64245549, 2020) | **ISRCTN64245549** (2020) Refugees Well School (Finland): Effectiveness of psychosocial school interventions for refugee and immigrant children. Https://Trialsearch.Who.Int/Trial2.Aspx?TrialID=ISRCTN64245549. | duplicate |
| (ISRCTN76460837, 2010) | **ISRCTN76460837** (2010) Multicultural Information on Depression: Effects of a multilingual information website intervention on the levels of depression literacy and depression related stigma in Greek–born and Italian–born immigrants living in Australia. Https://Trialsearch.Who.Int/Trial2.Aspx?TrialID=ISRCTN76460837. | duplicate |
| (ISRCTN89522188, 2014) | **ISRCTN89522188** (2014) North London Mother–Child–Education–Program (MOCEP) for Turkish–speaking families. Https://Trialsearch.Who.Int/Trial2.Aspx?TrialID=ISRCTN89522188. | study stopped |
| (ISRCTN96282810, 2022) | **ISRCTN96282810** (2022) A trial of the Self–Help Online program for Ukrainian refugees. Https://Trialsearch.Who.Int/Trial2.Aspx?TrialID=ISRCTN96282810. | wrong study design |
| (James et al. 2021) | **James LE, Welton–Mitchell C, Michael S, Santoadi F, Shakirah S, Hussin H, Anwar M, Kilzar L and James A** (2021) Development and Testing of a Community–Based Intervention to Address Intimate Partner Violence among Rohingya and Syrian Refugees: A Social Norms–Based Mental Health–Integrated Approach. *International Journal of Environmental Research and Public Health* **18**(21), 11674. https://doi.org/10.3390/ijerph182111674. | wrong outcome |
| (Jeon et al. 2020) | **Jeon S, Lee J, Jun JY, Park YS, Cho J, Choi J, Jeon Y and Kim SJ** (2020) The effectiveness of cognitive behavioral therapy on depressive symptoms in north korean refugees. *Psychiatry Investigation* **17**(7), 681–687. https://doi.org/10.30773/pi.2019.0134. | wrong study design |
| (Jespersen and Vuust, 2012) | **Jespersen KV and Vuust P** (2012) The Effect of Relaxation Music Listening on Sleep Quality in Traumatized Refugees: A Pilot Study. *Journal of Music Therapy* **49**(2), 205–229. | wrong study design |
| (Johnson et al. 2020) | **Johnson D, Zlotnick C, Hoffman L, Palmieri P, Johnson N, Holmes S and Ceroni T** (2020) A Randomized Controlled Trial Comparing HOPE Treatment and Present–Centered Therapy in Women Residing in Shelter With PTSD From Intimate Partner Violence. *Psychology of Women Quarterly* **44**(4), 539–553. | wrong population |
| (Jun et al. 2014) | **Jun WH, Hong SS and Yang S** (2014) Effects of a Psychological Adaptation Improvement Program for International Marriage Migrant Women in South Korea. *Asian Nursing Research* **8**(3), 232–238. | wrong study design |
| (Jung et al. 2019) | **Jung F, Rohr S, Konig H, Kersting A and Riedel–Heller S** (2019) HELP@APP: study design for the development and evaluation of a self–help app for traumatized Syrian refugees in Germany. *Gesundheitswesen* **81**(8), 672. | duplicate |
| (Kaltman et al. 2016) | **Kaltman S, de Mendoza AH, Serrano A and Gonzales FA** (2016) A Mental Health Intervention Strategy for Low–Income, Trauma–Exposed Latina Immigrants in Primary Care: A Preliminary Study. *American Journal of Orthopsychiatry* **86**(3), 345–354. https://doi.org/10.1037/ort0000157. | wrong study design |
| (Kangaslampi et al. 2015) | **Kangaslampi S, Garoff F and Peltonen K** (2015) Narrative exposure therapy for immigrant children traumatized by war: Study protocol for a randomized controlled trial of effectiveness and mechanisms of change. *BMC Psychiatry* **15**, 127. | duplicate |
| (Kankaanpaa et al. 2022) | **Kankaanpaa R, Aalto S, Vanska M, Lepisto R, Punamaki R–L, Soye E, Watters C, Andersen A, Hilden PK, Derluyn I, Verelst A and Peltonen** **K** (2022) Effectiveness of psychosocial school interventions in Finnish schools for refugee and immigrant children, ‘Refugees Well School’ in Finland (RWS–FI): A protocol for a cluster randomized controlled trial. *Trials* **23**(1), 79. https://doi.org/10.1186/s13063–021–05715–6. | duplicate |
| (Kataoka et al. 2003) | **Kataoka SH, Stein BD, Jaycox LH, Wong M, Escudero P, Tu W, Zaragoza C and Fink A** (2003) A school–based mental health program for traumatized latino immigrant children. *Journal of the American Academy of Child and Adolescent Psychiatry* **42**(3), 311–318. https://doi.org/10.1097/00004583–200303000–00011. | wrong study design |
| (Kayrouz et al. 2016) | **Kayrouz R, Dear BF, Karin E, Gandy M, Fogliati VJ, Terides MD and Titov N** (2016) A pilot study of self–guided internet–delivered cognitive behavioural therapy for anxiety and depression among Arabs. *Internet Interventions* **3**, 18–24. | wrong study design |
| (Kevers et al. 2022) | **Kevers R, Spaas C, Derluyn I, de Smet S, Van den Noortgate W, Colpin H and De Haene L** (2022) The Effect of a School–Based Creative Expression Program on Immigrant and Refugee Children’s Mental Health and Classroom Social Relationships: A Cluster Randomized Trial in Elementary School. *American journal of orthopsychiatry* **92**(5), 599–615. https://doi.org/10.1037/ort0000628. | wrong population |
| (Kha et al. 2022) | **Kha J, Rapee RM and Bayer JK** (2022) Acceptability and outcomes of the cool little kids parenting group program for culturally and linguistically diverse families within an australian population–based study. Child Psychiatry and Human Development, Achenbach, T.M and Rescorla, L.A (2000) Manual for the ASEBA Preschool Forms and Profiles. PhD dissertation, Department of Psychiatry, University of Vermont, Burlington. https://doi.org/10.1007/s10578–021–01293–5. | wrong population |
| (Kiang and Baden, 2020) | **Kiang M and Baden AL** (2020) A School–Based Group Intervention for Immigrant Adolescents: Addressing Acculturative Stress and Facilitating Ethnic Identity Development, School Connectedness and Related Psychosocial Outcomes. PhD dissertation, Montclair State University, New Jersey. https://www.proquest.com/dissertations–theses/school–based–group–intervention–immigrant/docview/2413008178/se–2?accountid=14836. | wrong study design |
| (Kitchener and Jorm, 2008) | **Kitchener BA and Jorm AF** (2008) Mental health first aid: An international programme for early intervention. *Early Intervention in Psychiatry* **2**(1), 55–61. https://doi.org/10.1111/j.1751–7893.2007.00056.x. | wrong population |
| (Kuehn, 2020) | **Kuehn BM** (2020) Guided Self–help Program May Help Refugees in Distress. *JAMA: Journal of the American Medical Association* **323**(11), 1033–1033. | wrong study design |
| (Lachal et al. 2020) | **Lachal J, Moro MR, Carretier E, Simon A, Barry C, Falissard B and Rouquette A** (2020) Assessment of transcultural psychotherapy to treat resistant major depressive disorder in children and adolescents from migrant families: Protocol for a randomized controlled trial using mixed method and Bayesian approaches. *International Journal of Methods in Psychiatric Research* **29**(4), 1–10. | ongoing |
| (Lamonica et al. 2020) | **Lamonica V, Ragazzi E and Sella L** (2020) Including adolescent migrants in school through VET approach: Evidence from a pilot action in Italy. *Empirical Research in Vocational Education and Training* **12**(1). | wrong outcome |
| (Laugesen and Bruhn, 2022) | **Laugesen H and Bruhn M** (2022) Developing and evaluating an integrated care model for unemployed trauma–affected refugees with PTSD: An important step towards cross–sectoral collaborative treatment. *International Journal of Integrated Care (IJIC)* **22**, 1–2. https://doi.org/10.5334/ijic.ICIC22081. | duplicate |
| (Lawrence and Falaye, 2020) | **Lawrence KC and Falaye AO** (2020) Trauma‐focused counselling and social effectiveness skills training interventions on impaired psychological functioning of internally displaced adolescents in Nigeria. *Journal of Community and Applied Social Psychology* **30**(6), 616–627. | wrong study design |
| (Le et al. 2008) | **Le H–N, Lara MA and Perry DF** (2008) Recruiting Latino women in the U.S. and women in Mexico in postpartum depression prevention research. *Archives of Women’s Mental Health* **11**(2), 159–169. https://doi.org/10.1007/s00737–008–0009–6. | duplicate |
| (Le et al. 2021) | **Le H–N, Perry DF, Villamil Grest C, Genovez M, Lieberman K, Ortiz–Hernandez S and Serafini C** (2021) A mixed methods evaluation of an intervention to prevent perinatal depression among Latina immigrants. *Journal of Reproductive and Infant Psychology* **39**(4), 382–394. | wrong study design |
| (Lebiger–Vogel et al. 2015) | **Lebiger–Vogel J, Rickmeyer C, Busse A, Fritzemeyer K, Ruger B and Leuzinger–Bohleber** **M** (2015) FIRST STEPS – a randomized controlled trial on the evaluation of the implementation and effectiveness of two early prevention programs for promoting the social integration and a healthy development of children with an immigrant background from 0–3. *BMC Psychology* **3**(1), 21–21. | duplicate |
| (Lebiger–Vogel et al. 2022) | **Lebiger–Vogel J, Rickmeyer C, Leuzinger–Bohleber M and Meurs P** (2022) Fostering Emotional Availability in Mother–Child–Dyads With an Immigrant Background: A Randomized–Controlled–Trial on the Effects of the Early Prevention Program First Steps. *Frontiers in Psychology* **13**, 790244. | awaiting assessment |
| (Lee C.S. et al. 2010) | **Lee CS, Caetano R, Hernandez L, Colby SM, Rohsenow DR and Lopez SR** (2010) Drinking related to acculturation stress, not acculturation, among immigrant Latinos. Alcoholism: Clinical and Experimental Research, **34**(6), 229A. https://doi.org/10.1111/j.1530–0277.2010.01210.x. | wrong population |
| (Lee, 2015) | **Lee H** (2015) The Psychological Support for Adaptation of North Korean Refugees Adolescents to Their South Korean Communities by Using Group Play Therapy. *North korean studies review* **19**(2), 211–237. | no full text |
| (Lempertz et al. 2020) | **Lempertz D, Wichmann M, Enderle E, Stellermann–Strehlow K, Pawils S and Metzner F** (2020) Pre–post study to assess EMDR–based group therapy for traumatized refugee preschoolers. *Journal of EMDR Practice and Research* **14**(1), 31–45. | wrong study design |
| (Liem et al. 2020) | **Liem A, Garabiles MR, Pakingan KA, Chen W, Lam AIF, Burchert S and Hall BJ** (2020) A digital mental health intervention to reduce depressive symptoms among overseas Filipino workers: Protocol for a pilot hybrid type 1 effectiveness–implementation randomized controlled trial. *Implementation Science Communications* **1**, 96. | ongoing |
| (Litrownik et al. 2000) | **Litrownik AJ, Elder JP, Campbell NR, Ayala GX, Slymen DJ, Parra–Medina D, Zavala FB and Lovato CY** (2000) Evaluation of a tobacco and alcohol use prevention program for hispanic migrant adolescents: Promoting the protective factor of parent–child communication. *Preventive Medicine* **31**(2 I), 124–133. https://doi.org/10.1006/pmed.2000.0698. | wrong outcome |
| (Lofvander et al. 1997) | **Lofvander M, Engstrom A, Theander H and Furhoff A–K** (1997) Rehabilitation of young immigrants in primary care: A comparison between two treatment models. *Scandinavian Journal of Primary Health Care* **15**(3), 123–128. https://doi.org/10.3109/02813439709018501. | wrong outcome |
| (Logie et al. 2022) | **Logie CH, Okumu M, Kortenaar J–L, Gittings L, Khan N, Hakiza R, Kibuuka Musoke D, Nakitende A, Katisi B, Kyambadde P, Khan T, Lester R and Mbuagbaw L** (2022) Mobile Health–Supported Virtual Reality and Group Problem Management Plus: Protocol for a Cluster Randomized Trial Among Urban Refugee and Displaced Youth in Kampala, Uganda (Tushirikiane4MH, Supporting Each Other for Mental Health) *JMIR Research Protocols* **11**(12), e42342–e42342. https://doi.org/10.2196/42342. | ongoing |
| (Lohmann, 2019) | **Lohmann JC** (2019) E.03.02 Treatment of trauma–affected refugees. *European Neuropsychopharmacology* **29**(1), S32–S33. https://doi.org/10.1016/j.euroneuro.2018.11.1097. | already included |
| (Lopez–Maya et al. 2019) | **Lopez–Maya E, Olmstead R and Irwin MR** (2019) Mindfulness meditation and improvement in depressive symptoms among Spanish- and English- speaking adults: A randomized, controlled, comparative efficacy trial. *PLoS ONE* **14**(7), e0219425. https://doi.org/10.1371/journal.pone.0219425. | wrong population |
| (Lu et al. 2022) | **Lu S, Lyu R, Hu H, Ho KKM, Barry TJ, Black D and Wong DFK** (2022) Parallel Parent–Child Mindfulness Intervention Among Chinese Migrant Families: A Mixed–Methods Feasibility Study. *Research on Social Work Practice* **32**(8), 925–939. https://doi.org/10.1177/10497315221089684. | wrong study design |
| (Lund et al. 2020) | **Lund HN, Pedersen IN, Johnsen SP, Heymann–Szlachcinska AM, Tuszewska M, Bizik G, Larsen JI, Kulhay E, Larsen A, Grønbech B, Østermark H, Borup H, Valentin JB and Mainz J** (2020) Music to improve sleep quality in adults with depression–related insomnia (MUSTAFI): Study protocol for a randomized controlled trial. *Trials* **21**(1), 305. | wrong population |
| (Lutenbacher et al. 2018) | **Lutenbacher M, Elkins T, Dietrich MS and Riggs A** (2018) The efficacy of using peer mentors to improve maternal and infant health outcomes in Hispanic families: Findings from a randomized clinical trial. *Maternal and Child Health Journal* 22, S124. | wrong intervention |
| (Lutenbacher et al. 2022) | **Lutenbacher M, Elkins T and Dietrich MS** (2022) Using Community Health Workers to Improve Health Outcomes in a Sample of Hispanic Women and Their Infants: Findings from a Randomized Controlled Trial. *Hispanic Health Care International: The Official Journal of the National Association of Hispanic* Nurses. **21**(3). https://doi.org/10.1177/15404153221107680. | wrong intervention |
| (Magana et al. 2021) | **Magana S, Tejero Hughes M, Salkas K, Gonzales W, Nunez G, Morales M, Garcia Torres M and Moreno–Angarita M** (2021) Implementing a Parent Education Intervention in Colombia: Assessing Parent Outcomes and Perceptions Across Delivery Modes. *Focus on Autism and Other Developmental Disabilities* **36**(3), 165–175. https://doi.org/10.1177/1088357620986947. | wrong population |
| (Marsiglia et al. 2010) | **Marsiglia FF, Bermudez–Parsai M and Coonrod D** (2010) Familias Sanas: An intervention designed to increase rates of postpartum visits among Latinas. *Journal of Health Care for the Poor and Underserved* **21**(3), 119–131. | wrong outcome |
| (Mehrabi et al. 2011) | **Mehrabi T, Musavi T, Ghazavi Z, Zandieh Z and Zamani A** (2011) The impact of group therapy training on social communications of Afghan immigrants. *Iranian Journal of Nursing and Midwifery Research* **16**(2), 148–152. | wrong outcome |
| (Meir et al. 2012) | **Meir Y, Slone M, Levis M, Reina L and Livni YBD** (2012) Crisis intervention with children of illegal migrant workers threatened with deportation. *Professional Psychology: Research and Practice* **43**(4), 298–305. | wrong study design |
| (Meir et al. 2014) | **Meir Y, Slone M and Levis M** (2014) A Randomized Controlled Study of a Group Intervention Program to Enhance Mental Health of Children of Illegal Migrant Workers. *Child and Youth Care Forum* **43**(2), 165–180. | wrong population |
| (Meyer DeMott et al. 2017) | **Meyer DeMott MA, Jakobsen M, Wentzel–Larsen T and Heir T** (2017) A controlled early group intervention study for unaccompanied minors: Can Expressive Arts alleviate symptoms of trauma and enhance life satisfaction?. *Scandinavian Journal of Psychology* **58**(6), 510–518. | wrong study design |
| (Mihlbauer, 1984) | **Mihlbauer TC** (1984) *The impact of support groups on displaced homemakers’ self–esteem, locus of control and level of personal responsibility (unemployed women)*. PhD dissertation, Southern Illinois University, Carbondale, Illinois. https://www.proquest.com/dissertations–theses/impact–support–groups–on–displaced–homemakers/docview/303327127/se–2?accountid=14836. | no full text |
| (Miller et al. 2020) | **Miller KE, Arnous M, Tossyeh F, Chen A, Bakolis I, Koppenol–Gonzalez GV, Nahas N and Jordans MJD** (2020) Protocol for a randomized control trial of the caregiver support intervention with Syrian refugees in Lebanon. *Trials* **21**(1), 277. | duplicate |
| (Miller et al. 2020) | **Miller K, Koppenol–Gonzalez G, Ali J, Steen F, Sassine M and Jordans M** (2020) A Randomised Controlled Trial of the I–Deal Life Skills Intervention with Syrian Refugee Adolescents in Northern Lebanon. *Intervention* **18**(2), 119–128. | wrong population |
| (Mitschke et al. 2013) | **Mitschke DB, Aguirre RTP and Sharma B** (2013) Common Threads: Improving the Mental Health of Bhutanese Refugee Women Through Shared Learning. *Social Work in Mental Health* **11**(3), 249–266. | wrong intervention |
| (Moadel, 2011) | **Moadel A** (2011) Psycho–educational and spiritual interventions for low income cancer patients: Results of a randomized versus patient preference trial. *Psycho-Oncology* **20**(1), 30–31. https://doi.org/10.1111/j.1755–148X.2011.01915.x. | wrong population |
| (Mufson, 2008) | **Mufson L** (2008) Group interpersonal therapy reduces depression in adolescent survivors of war. *Evidence–Based Mental Health* **11**(1), 17–17. | duplicate |
| (Mulligan et al. 2017) | **Mulligan CJ, Clukay C, Quinlan J, Dajani R, Hamadmad D, Abudayyeh G and Panter–Brick C** (2017) Genetics of risk and resilience in Syrian refugee youth. *American Journal of Physical Anthropology* **162**(64), 294–295. https://doi.org/10.1002/ajpa.23210. | wrong population |
| (Murray et al. 2018) | **Murray L, Hall B, Dorsey S, Ugueto A, Puffer E, Sim A, Ismael A, Bass J, Akiba C, Lucid L Harrison J, Erikson A and Bolton PA** (2018) An evaluation of a common elements treatment approach for youth in Somali refugee camps. *Global Mental Health* **5**. | wrong study design |
| (Naeem et al. 2021) | **Naeem F, Tuck A, Mutta B, Dhillon P, Thandi G, Kassam A, Farah N, Ashraf A, Husain M, Husain M, Vasiliadis H-M, Sanches M, Munshi T, Abbott M, Watters N, Kidd SA, Ayub M and Kwame McKenzie K** (2021) Protocol for a multi–phase, mixed methods study to develop and evaluate culturally adapted CBT to improve community mental health services for Canadians of south Asian origin. *Trials* **22**(1). | ongoing |
| (NCT00280319, 2006) | **NCT00280319** (2006) Study of Treatments for Depression Among Displaced Adolescents in Northern Uganda. Https://Clinicaltrials.Gov/Show/NCT00280319. | duplicate |
| (NCT00879853, 2009) | **NCT00879853** (2009) Trial of Mental Health Treatment for Darfur Refugees in Cairo. Https://Clinicaltrials.Gov/Show/NCT00879853. | ongoing |
| (NCT00917397, 2009) | **NCT00917397** (2009) The Outcome of Treatment of Traumatised Refugees With Psychotherapy and/or Antidepressants. Https://Clinicaltrials.Gov/Show/NCT00917397. | duplicate |
| (NCT01288690, 2011) | **NCT01288690** (2011) Stress Reduction in Middle Eastern Refugees. Https://Clinicaltrials.Gov/Show/NCT01288690. | duplicate |
| (NCT01668992, 2012) | **NCT01668992** (2012) Impact Evaluation of a Family–based Intervention With Burmese Migrant and Displaced Children and Families in Tak Province, Thailand. Https://Clinicaltrials.Gov/Show/NCT01668992. | duplicate |
| (NCT01847742, 2013) | **NCT01847742** (2013) EMDR Intervention for Psychological Trauma Among Syrian Refugees. Https://Clinicaltrials.Gov/Show/NCT01847742. | duplicate |
| (NCT01856673, 2013) | **NCT01856673** (2013) Evaluation of Two Community–based Mental Health Interventions for Violence–Displaced Afro–Descendants in Colombia. Https://Clinicaltrials.Gov/Show/NCT01856673. | wrong outcome |
| (NCT01955538, 2013) | **NCT01955538** (2013) The Effect of BAT Versus Mixed Physical Activity as add–on Treatment for Traumatised Refugees. Https://Clinicaltrials.Gov/Show/NCT01955538. | duplicate |
| (NCT02114593, 2014) | **NCT02114593** (2014) Ladnaan—An Evaluation of a Parent Support Program for Somali Parents. Https://Clinicaltrials.Gov/Show/NCT02114593. | duplicate |
| (NCT02334566, 2014) | **NCT02334566** (2014) Lending a Hand to Our Future: PTSD in Refugee Children and Youth. Https://Clinicaltrials.Gov/Show/NCT02334566. | study stopped |
| (NCT02493439, 2015) | **NCT02493439** (2015) A Positive Activity Intervention to Promote Wellbeing in Romanian Immigrants. *Https://Clinicaltrials.Gov/Show/NCT02493439*. | ongoing |
| (NCT02874235, 2016) | **NCT02874235** (2016) Music Therapy and Treatment as Usual. Https://Clinicaltrials.Gov/Show/NCT02874235. | duplicate |
| (NCT03075475, 2017) | **NCT03075475** (2017) Effectiveness Study of a Treatment to Improve the Mental Health of Children and Adolescents. *Https://Clinicaltrials.Gov/Show/NCT03075475*. | ongoing |
| (NCT03162679, 2017) | **NCT03162679** (2017) Culture–sensitive Emotion Regulation Group Therapy for Traumatized Refugees. Https://Clinicaltrials.Gov/Show/NCT03162679. | duplicate |
| (NCT03265847, 2017) | **NCT03265847** (2017) The weWomen and ourCircle Intervention for Immigrant, Refugee and Indigenous Women. Https://Clinicaltrials.Gov/Show/NCT03265847. | duplicate |
| (NCT03461640, 2018) | **NCT03461640** (2018) Community Based Doulas for Migrant Women in Labour and Birth in Sweden—A Randomised Controlled Trial. Https://Clinicaltrials.Gov/Show/NCT03461640. | duplicate |
| (NCT03545282, 2018) | **NCT03545282** (2018) Randomized Trial to Reduce Mental Health Disparities in Latina Immigrant Women. Https://Clinicaltrials.Gov/Show/NCT03545282. | wrong study design |
| (NCT03567083, 2018) | **NCT03567083** (2018) Implementation of Problem Management Plus (PM+) in Adult Syrian Refugees in Turkey: Pilot. Https://Clinicaltrials.Gov/Show/NCT03567083. | duplicate |
| (NCT03571347, 2018) | **NCT03571347** (2018) Self Help Plus for Asylum Seekers and Refugees in Europe. Https://Clinicaltrials.Gov/Show/NCT03571347. | duplicate |
| (NCT03587896, 2018) | **NCT03587896** (2018) Implementation of Self Help Plus in Adult Syrian Refugees in Turkey (RE–DEFINE) Https://Clinicaltrials.Gov/Show/NCT03587896. | duplicate |
| (NCT03749278, 2018) | **NCT03749278** (2018) Latina Friends Motivating the Soul (ALMA) Https://Clinicaltrials.Gov/Show/NCT03749278. | duplicate |
| (NCT03760627, 2018) | **NCT03760627** (2018) Evaluation of A Mindfulness Resiliency Training Program for Refugees Living in Jordan. *Https://Clinicaltrials.Gov/Show/NCT03760627*. | ongoing |
| (NCT03796065, 2019) | **NCT03796065** (2019) Addressing Mental Health Disparities in Refugee Children. *Https://Clinicaltrials.Gov/Show/NCT03796065*. | ongoing |
| (NCT03830008, 2019) | **NCT03830008** (2019) Scaling–up Psychological Interventions With Syrian Refugees in Switzerland. Https://Clinicaltrials.Gov/Show/NCT03830008. | duplicate |
| (NCT03887312, 2019) | **NCT03887312** (2019) Phone–Delivered Psychological Intervention (t–CETA) for Mental Health Problems in 8–16 Year–Old Syrian Refugee Children. *Https://Clinicaltrials.Gov/Show/NCT0388731.* | ongoing |
| (NCT03912077, 2019) | **NCT03912077** (2019) Implementing Psychosocial Interventions to Syrian Refugee Women Who Are Exposed to Psychological Trauma. Https://Clinicaltrials.Gov/Show/NCT03912077. | duplicate |
| (NCT03960892, 2019) | **NCT03960892** (2019) Implementation of Group Problem Management Plus (PM+) in Adult Syrian Refugees in Turkey: RCT (STRENGTHS) Https://Clinicaltrials.Gov/Show/NCT03960892. | duplicate |
| (NCT04031170, 2019) | **NCT04031170** (2019) Filipino Family Health Initiative. *Https://Clinicaltrials.Gov/Show/NCT04031170*. | ongoing |
| (NCT04088487, 2019) | **NCT04088487** (2019) New in Town—Mobile–Based Intervention for Migrants. Https://Clinicaltrials.Gov/Show/NCT04088487. | duplicate |
| (NCT04098276, 2019) | **NCT04098276** (2019) It’s WeWomen Plus Intervention for Health, Safety and Empowerment. Https://Clinicaltrials.Gov/Show/NCT04098276. | wrong outcome |
| (NCT04244864, 2020) | **NCT04244864** (2020) Cross–sectoral Collaboration in Psychosocial Treatment of Trauma–affected Refugees. *Https://Clinicaltrials.Gov/Show/NCT04244864*. | ongoing |
| (NCT04254198, 2020) | **NCT04254198** (2020) Tertulias Social Isolation Women’s Groups Study. Https://Clinicaltrials.Gov/Show/NCT04254198. | duplicate |
| (NCT04278157, 2020) | **NCT04278157** (2020) Culturally Centered CBT for Latinx Youth. Https://Clinicaltrials.Gov/Show/NCT04278157. | wrong population |
| (NCT04380259, 2020) | **NCT04380259** (2020) Mindfulness–Based Trauma Recovery for Refugees (MBTR–R) Https://Clinicaltrials.Gov/Show/NCT04380259. | duplicate |
| (NCT04453709, 2020) | **NCT04453709** (2020) Family–centered Mental Health Promotion Intervention. Https://Clinicaltrials.Gov/Show/NCT04453709. | duplicate |
| (NCT04656353, 2020) | **NCT04656353** (2020) An Impact Assessment of a Culturally Tailored Online Psychosocial Program for Chinese Immigrant Women With Breast Cancer. Https://Clinicaltrials.Gov/Show/NCT04656353. | ongoing |
| (NCT04771650, 2021) | **NCT04771650** (2021) A Hybrid Effectiveness Implementation Study of Latino/a Alcohol and Drug Users. Https://Clinicaltrials.Gov/Show/NCT04771650. | ongoing |
| (NCT04816032, 2021) | **NCT04816032** (2021) "Expressive Writing Psychological Health Migrants (EWPHM) Https://Clinicaltrials.Gov/Show/NCT04816032. | duplicate |
| (NCT04831463, 2021) | **NCT04831463** (2021) The Effect of the Program on the Health Perceptions and Responsibilities of Immigrant Men on Utilizing Healthcare Services. Https://Clinicaltrials.Gov/Show/NCT04831463. | wrong intervention |
| (NCT04832035, 2021) | **NCT04832035** (2021) Integration of Refugees Into Public Mental Health Care. Https://Clinicaltrials.Gov/Show/NCT04832035. | ongoing |
| (NCT04917822, 2021) | **NCT04917822** (2021) Project KING: To Strengthen Parent–child Relationship and Adaptation. Https://Clinicaltrials.Gov/Show/NCT04917822. | ongoing |
| (NCT04993534, 2021) | **NCT04993534** (2021) RESPOND: Improving the Preparedness of Health Systems to Reduce Mental Health and Psychosocial Concerns Resulting From the COVID–19 Pandemic. Https://Clinicaltrials.Gov/Show/NCT04993534. | ongoing |
| (NCT05092542, 2021) | **NCT05092542** (2021) Multilevel Community–Based Mental Health Intervention to Address Structural Inequities and Adverse Disparate Consequences of COVID–19 Pandemic on Latinx Immigrant and African Refugees. Https://Clinicaltrials.Gov/Show/NCT05092542. | ongoing |
| (NCT05171192, 2021) | **NCT05171192** (2021) Culturally Adapted CMAP Plus LTP for Refugee Mothers With History of Self–harm in Pakistan. Https://Clinicaltrials.Gov/Show/NCT05171192. | ongoing |
| (NCT05208359, 2022) | **NCT05208359** (2022) Supporting Refugee and Immigrant Youth’s Mental Health. Https://Clinicaltrials.Gov/Show/NCT05208359. | ongoing |
| (NCT05233345, 2022) | **NCT05233345** (2022) Online Occupational Therapy on Occupational Balance, Well–being and Quality of Life in Syrian Refugee Children. Https://Clinicaltrials.Gov/Show/NCT05233345. | wrong intervention |
| (NCT05265611, 2022) | **NCT05265611** (2022) Syrian Refugee Youth as Community Mental Health Workers–Lebanon. Https://Clinicaltrials.Gov/Show/NCT05265611. | ongoing |
| (NCT05281575, 2022) | **NCT05281575** (2022) Evaluation of Baby Friendly Spaces in Cox’s Bazar, Bangladesh. Https://Clinicaltrials.Gov/Show/NCT05281575. | ongoing |
| (NCT05370443, 2022) | **NCT05370443** (2022) Effectiveness of an Online Life Skills–based Intervention on Mental Health Among Arabic Adolescents in Klang Valley, Malaysia. Https://Clinicaltrials.Gov/Show/NCT05370443. | ongoing |
| (NCT05407337, 2022) | **NCT05407337** (2022) The Effect of Narrative Exposure Therapy Intervention on Post–traumatic Stress Dissorder and Personal Recovery in Refugees and Asylum Seekers of Sindiane Programme. Https://Clinicaltrials.Gov/Show/NCT05407337. | ongoing |
| (NCT05471921, 2022) | **NCT05471921** (2022) Effectiveness of an Evidence–based Stepped Care System for Alcohol and Other Drug Use Problems Among Congolese Refugees in Zambia. Https://Clinicaltrials.Gov/Show/NCT05471921. | ongoing |
| (NCT05476185, 2022) | **NCT05476185** (2022) USCRI READY4Life Program. Https://Clinicaltrials.Gov/Show/NCT05476185. | wrong outcome |
| (NCT05512624, 2022) | **NCT05512624** (2022) Integrating a Mental Health Intervention Into Primary Health Care for Refugees. Https://Clinicaltrials.Gov/Show/NCT05512624. | ongoing |
| (NCT05515094, 2022) | **NCT05515094** (2022) Strengthening Women’s Agency During Pregnancy. Https://Clinicaltrials.Gov/Show/NCT05515094. | ongoing |
| (NCT05616286, 2022) | **NCT05616286** (2022) Mindfulness–SOS for Refugees: Randomized Controlled Trial. Https://Clinicaltrials.Gov/Show/NCT05616286. | ongoing |
| (Neville et al. 2022) | **Neville SE, DiClemente–Bosco K, Chamlagai LK, Bunn M, Freeman J, Berent JM, Gautam B, Abdi A and Betancourt TS** (2022) Investigating Outcomes of a Family Strengthening Intervention for Resettled Somali Bantu and Bhutanese Refugees: An Explanatory Sequential Mixed Methods Study. *International journal of environmental research and public health* **19**(19) https://doi.org/10.3390/ijerph191912415. | duplicate |
| (Nordbrandt et al. 2015) | **Nordbrandt MS, Carlsson J, Lindberg LG, Sandahl H and Mortensen EL** (2015) Treatment of traumatised refugees with basic body awareness therapy versus mixed physical activity as add–on treatment: Study protocol of a randomised controlled trial. *Trials* **16**, 477. | duplicate |
| (NTR2303, 2010) | **NTR2303** (2010) Self–help course with guidance on the Internet for Turkish migrants with depression. Https://Trialsearch.Who.Int/Trial2.Aspx?TrialID=NTR2303. | wrong study design |
| (NTR6842, 2017) | **NTR6842** (2017) STRENGTHS: Fostering responsive mental health systems in the Syrian refugee crisis. Https://Trialsearch.Who.Int/Trial2.Aspx?TrialID=NTR6842. | duplicate |
| (Oh and Moon, 2019) | **Oh SH and Moon J–Y** (2019) The Effects of Song–Focused Music Therapy Program Based on Positive Psychology on Subjective Well–being and Optimism of Migrant Women from Multi–cultural Families. *Korean Journal of Music Therapy* **21**(1), 71–93. | no full text |
| (Onyut et al. 2004) | **Onyut LP, Neuner F, Schauer E, Ertl V, Odenwald MG, Schauer M and Elbert T** (2004) The Nakivale Camp Mental Health Project: Building local competency for psychological assistance to traumatised refugees. *Intervention* **2**(2), 90–107. | wrong study design |
| (Orang et al. 2022) | **Orang TM, Missmahl I, Thoele AM, Valensise L, Brenner A, Gardisi M, ... and Kluge U** (2022) New directions in the mental health care of migrants, including refugees—A randomized controlled trial investigating the efficacy of value‐based counselling. *Clinical Psychology and Psychotherapy* **29**(4), 1433–1446. | duplicate |
| (Oren–Schwartz et al. 2022) | **Oren–Schwartz R, Aizik–Reebs A, Yuval K, Hadash Y and Bernstein A** (2022) Effect of Mindfulness–Based Trauma Recovery for Refugees on Shame and Guilt in Trauma Recovery Among African Asylum–Seekers. *Emotion* **23**(3), 622–632. https://doi.org/10.1037/emo0001126. | duplicate |
| (Ornelas et al. 2019) | **Ornelas IJ, Doyle SR, Torres VN, Serrano SE, Duran B and Donovan DM** (2019) Vida PURA: Results from a pilot randomized trial of a culturally adapted screening and brief intervention to reduce unhealthy alcohol use among Latino day laborers. *Translational Behavioral Medicine* **9**(6), 1233–1243. https://doi.org/10.1093/tbm/ibz071. | wrong outcome |
| (Ornelas et al. 2022) | **Ornelas IJ, Perez G, Maurer S, Gonzalez S, Childs V, Price C, Nelson AK, Perez Solorio SA, Tran A and Rao D** (2022) Amigas Latinas Motivando el Alma: In–Person and Online Delivery of an Intervention to Promote Mental Health Among Latina Immigrant Women. *Journal of integrative and complementary* *medicine* **28**(10). https://doi.org/10.1089/jicm.2022.0491. | wrong study design |
| (Osman et al. 2017) | **Osman F, Klingberg–Allvin M, Flacking R and Schön U–K** (2017) Ladnaan: Evaluation of a Culturally Tailored Parenting Support Program to Somali–Born Parents. PhD dissertation, Karolinska Institutet, Sweden. https://www.proquest.com/dissertations–theses/ladnaan–evaluation–culturally–tailored–parenting/docview/2599084362/se–2?accountid=14836. | duplicate |
| (Osman et al. 2021) | **Osman F, Vixner L, Flacking R, Klingberg–Allvin M, Schon U–K and Salari R** (2021) Impact of a culturally tailored parenting programme on the mental health of Somali parents and children living in Sweden: A longitudinal cohort study. *BMJ Open* 11(8), e045067. https://doi.org/10.1136/bmjopen–2020–045067. | duplicate |
| (Osman, 2017) | **Osman F** (2017) A Support Program for Somali–born Parents on Children’s Behavioral Problems. *Pediatrics* **139**(3), 1–9. | duplicate |
| (Ostuzzi et al. 2021) | **Ostuzzi G, Purgato M, Barbui C and Tedeschi F** (2021) Self–Help Plus (SH+) for preventing mental disorders in refugees and asylum seekers: The RE–DEFINE European randomized trial. | duplicate |
| (Ozcan, 2019) | **Ozcan Z** (2019) An experimental research on the role of social support in reducing the traumatic effects of immigration. *Bilimname* **37**(1), 1001–1028. | wrong study design |
| (Page–Reeves et al. 2021) | **Page–Reeves J, Murray–Krezan C, Regino L, Perez J, Bleecker M, Perez D, Wagner B, Tigert S, Bearer EL and Willging CE** (2021) A randomized control trial to test a peer support group approach for reducing social isolation and depression among female Mexican immigrants. *BMC Public Health* **21**(1), 119. | ongoing |
| (Panter–Brick et al. 2018) | **Panter–Brick C, Dajani R, Eggerman M, Hermosilla S, Sancilio A and Ager A** (2018) Insecurity, distress and mental health: Experimental and randomized controlled trials of a psychosocial intervention for youth affected by the Syrian crisis. *Journal of Child Psychology and Psychiatry and Allied Disciplines* **59**(5), 523–541. https://doi.org/10.1111/jcpp.12832. | wrong population |
| (Pantin et al. 2003) | **Pantin H, Coatsworth J, Feaster D, Newman F, Briones E, Prado G, Schwartz S and Szapocznik J** (2003) Familias Unidas: The efficacy of an intervention to promote parental investment in Hispanic immigrant families. *Prevention Science* **4**(3), 189–201. | wrong outcome |
| (Park and Jung, 2017) | **Park SH and Jung TY** (2017) The Effects of Horticultural Activity Based upon REBT Group Counseling on the Self–Expression and the Degree of Depression of the Female Marriage Immigrants’. *Journal of People, Plants and Environment* **20**(2), 165–174. | wrong study design |
| (Park et al. 2020) | **Park JK, Park J, Elbert T and Kim SJ** (2020) Effects of Narrative Exposure Therapy on Posttraumatic Stress Disorder, Depression and Insomnia in Traumatized North Korean Refugee Youth. *Journal of Traumatic Stress* **33**(3), 353–359. https://doi.org/10.1002/jts.22492. | wrong study design |
| (Patras et al. 2021) | **Patras J, Saus M, Douglas M, Bjørknes R, Gammelsæter S, Rasmussen L–MP, Halvorsen T, Haug IM, Risholm R, Øktedalen T, Jakobsen R and Neumer SP** (2021) Parenting interventions for families with refugee backgrounds: A randomized factorial. mixed–methods design study protocol. *Trials* **22**(1), 790. | ongoing |
| (Peltonen and Kangaslampi, 2019) | **Peltonen K and Kangaslampi S** (2019) Treating children and adolescents with multiple traumas: A randomized clinical trial of narrative exposure therapy. *European Journal of Psychotraumatology* **10**(1), 1558708. | wrong population |
| (Peltonen et al. 2022) | **Peltonen K, Aalto S, Vanska M, Lepisto R, Punamaki R–L, Soye E, Watters C, de Wal Pastoor L, Derluyn I and Kankaanpaa R** (2022) Effectiveness of Promotive and Preventive Psychosocial Interventions on Improving the Mental Health of Finnish–Born and Immigrant Adolescents. *International Journal of* *Environmental Research and Public Health* **19**(6) https://doi.org/10.3390/ijerph19063686. | wrong population |
| (Pfeiffer et al. 2019) | **Pfeiffer E, Sachser C, Tutus D, Fegert JM and Plener PL** (2019) Trauma–focused group intervention for unaccompanied young refugees: ‘mein Weg’—Predictors of treatment outcomes and sustainability of treatment effects. *Child and Adolescent Psychiatry and Mental Health* **13**(1), 18. https://doi.org/10.1186/s13034–019–0277–0. | duplicate |
| (Pokhariyal et al. 2013) | **Pokhariyal GP, Rono RC and Munywoki S** (2013) Analysis of treatment methods for victims of torture in Kenya and east Africa region. *Traumatology* **19**(2), 107–117. | wrong population |
| (Ponguta et al. 2020) | **Ponguta LA, Issa G, Aoudeh L, Maalouf C, Hein SD, Zonderman AL, Katsovich L, Khoshnood K, Bick J, Awar A, Nourallah S, Householder S, Moore CC, Salah R, Britto PR and Leckman JF** (2020) Effects of the mother–child education program on parenting stress and disciplinary practices among refugee and other marginalized communities in Lebanon: a pilot randomized controlled trial. *Journal of the American Academy of Child and Adolescent Psychiatry* **59**(6), 727–738. | wrong population |
| (Potocky and Guskovict, 2019) | **Potocky M and Guskovict KL** (2019) Project MIRACLE: Increasing empathy among psychosocial support staff working with refugees through brief training in motivational interviewing. *Intervention* **17**(1), 59–68. | wrong population |
| (Poudel–Tandukar et al. 2022) | **Poudel–Tandukar K, Jacelon CS, Martell CR, Poudel KC, Rai S, Ramdam R, Laws H, Meyer JS, Bertone–Johnson ER and Hollon SD** (2022) Peer–led family–centred problem management plus for immigrants (PMP–I) for mental health promotion among immigrants in USA: protocol for a pilot, randomised controlled feasibility trial. *BMJ Open* **12**(5), e061353. https://doi.org/10.1136/bmjopen–2022–061353. | ongoing |
| (Puccinelli and Santisteban, 2017) | **Puccinelli M and Santisteban DA** (2017) *Treatment and Moderator Effects in a Randomized Controlled Trial of Culturally Informed and Flexible Family Treatment for Adolescents (CIFFTA): An Investigation of the Relationships between Stress, Acculturation and Parenting Practices*. PhD dissertation, University of Miami, Florida. https://www.proquest.com/dissertations–theses/treatment–moderator–effects–randomized–controlled/docview/1999329571/se–2?accountid=14836. | awaiting assessment |
| (Puffer et al. 2017) | **Puffer ES, Annan J, Sim AL, Salhi C and Betancourt TS** (2017) The impact of a family skills training intervention among Burmese migrant families in Thailand: A randomized controlled trial. *PLoS ONE* **12**(3), e0172611. https://doi.org/10.1371/journal.pone.0172611. | wrong outcome |
| (Purgato et al. 2019) | **Purgato M, Carswell K, Acarturk C, Au T, Akbai S, Anttila M, Baumgartner J, Bailey D, Biondi M, Bird M, Churchill R, Eskici S, Hansen LJ, Heron P, Ilkkursun Z, Kilian R, Koesters M, Lantta T, Nosè M, Ostuzzi G, Papola D, Popa M, Sijbrandij m, Tarsitani L, Tedeschi F, Turrini G, Uygun EVälimäki MA, Wancata J, White Ross, Zanini E, Cuijpers P, Barbui C and Van Ommeren M** (2019) Effectiveness and cost–effectiveness of Self–Help Plus (SH+) for preventing mental disorders in refugees and asylum seekers in Europe and Turkey: Study protocols for two randomised controlled trials. *BMJ Open* **9**(5), e030259. | duplicate |
| (Qiu et al. 2022) | **Qiu X, Li T, Fang Q, Huang L and Zheng X** (2022) Online and Offline Intervention for the Prevention of Postpartum Depression among Rural–to–Urban Floating Women: Study Protocol for a Randomized Control Trial. *International journal of environmental research and public health* **19**(13) https://doi.org/10.3390/ijerph19137951. | ongoing |
| (Quinlan et al. 2016) | **Quinlan R, Schweitzer R, Khawaja N and Griffin J** (2016) Evaluation of a school–based creative arts therapy program for adolescents from refugee backgrounds. *Arts in Psychotherapy* **47**, 72–78. | wrong study design |
| (Radlick et al. 2020) | **Radlick RL, Svedberg P, Nygren JM, Przedpelska S and Gammon D** (2020) Digitally Enhanced Mentoring for Immigrant Youth Social Capital: Protocol for a Mixed Methods Pilot Study and a Randomized Controlled Trial. *JMIR Research Protocols* **9**(3). | wrong outcome |
| (Rawlinson et al. 2020) | **Rawlinson R, Aslam RW, Burnside G, Chiumento A, Eriksson–Lee M, Humphreys A, Khan N, Lawrence D, McCluskey R, Mackinnon A, Orton L, Rahman A, Roberts E, Rosala–Hallas A, Edwards RT, Uwamaliya P, White RG, Winrow E and Dowrick C** (2020) Lay–therapist–delivered, low–intensity, psychosocial intervention for refugees and asylum seekers (PROSPER): Protocol for a pilot randomised controlled trial. *Trials* **21**(1), 367. | duplicate |
| (Regina Hechanova et al. 2018) | **Regina Hechanova M, Docena PS, Pena Alampay L, Acosta A, Porio EE, Melgar IE and Berger R** (2018) Evaluation of a resilience intervention for Filipino displaced survivors of Super Typhoon Haiyan. *Disaster prevention and management* **27**(3), 346–359. | wrong study design |
| (Reijneveld et al. 2003) | **Reijneveld SA, Westhoff MH and Hopman–Rock M** (2003) Promotion of health and physical activity improves the mental health of elderly immigrants: Results of a group randomised controlled trial among Turkish immigrants in the Netherlands aged 45 and over. *Journal of Epidemiology and Community Health* **57**(6), 405–411. | wrong intervention |
| (Robertson et al. 2019) | **Robertson CL, Halcon L, Hoffman SJ, Osman N, Mohamed A, Areba E, Savik K and Mathiason MA** (2019) Health Realization Community Coping Intervention for Somali Refugee Women. *Journal of Immigrant and Minority Health* **21**(5), 1077–1084. https://doi.org/10.1007/s10903–018–0804–8. | wrong study design |
| (Rogala et al. 2020) | **Rogala A, Szczepaniak M, Michalak N and Andersson G** (2020) Internet–based self–help intervention aimed at increasing social self–efficacy among internal migrants in Poland: Study protocol for a randomized controlled trial. *Internet Interventions* **21**, 100322. https://doi.org/10.1016/j.invent.2020.100322. | wrong outcome |
| (Rosner et al. 2017) | **Rosner R, Eberle–Sejari R, Ganser HG, Goldbeck L and Hagl M** (2017) Experiences With a Migration–Adapted Service in Case Management for Children and Adolescents in Need of Treatment After Child Abuse and Neglect. *Kindheit und entwicklung* **26**(4), 240–250. | no full text |
| (Rosner et al. 2020) | **Rosner R, Sachser C, Hornfeck F, Kilian R, Kindler H, Muche R, Muller LRF, Thielemann J, Waldmann T, Ziegenhain U, Unterhitzenberger J and Pfeiffer E** (2020) Improving mental health care for unaccompanied young refugees through a stepped–care approach versus usual care+: Study protocol of a cluster randomized controlled hybrid effectiveness implementation trial. *Trials* **21**(1), 1013. | ongoing |
| (Rousseau et al. 2005) | **Rousseau C, Drapeau A, Lacroix L, Bagilishya D and Heusch N** (2005) Evaluation of a classroom program of creative expression workshops for refugee and immigrant children. *Journal of Child Psychology and Psychiatry and Allied Disciplines* **46**(2), 180–185. https://doi.org/10.1111/j.1469–7610.2004.00344.x. | wrong population |
| (Rousseau et al. 2009) | **Rousseau C, Benoit M, Lacroix L and Gauthier M–F** (2009) Evaluation of a Sandplay Program for Preschoolers in a Multiethnic Neighborhood. *Journal of* *Child Psychology and Psychiatry* **50**(6), 743–750. | wrong population |
| (Rousseau et al. 2014) | **Rousseau C, Beauregard C, Daignault K, Petrakos H, Thombs BD, Steele R, Vasiliadis H–M and Hechtman L** (2014) A cluster randomized–controlled trial of a classroom–based drama workshop program to improve mental health outcomes among immigrant and refugee youth in special classes. *PLoS ONE* **9**(8), e104704. https://doi.org/10.1371/journal.pone.0104704. | wrong population |
| (Russell et al. 2021) | **Russell GM, Long K, Lewis V, Enticott JC, Gunatillaka N, Cheng I–H, Marsh G, Vasi S, Advocat J, Saito S, Song H, Casey S, Smith M and Harris MF** (2021) OPTIMISE: a pragmatic stepped wedge cluster randomised trial of an intervention to improve primary care for refugees in Australia. *The Medical Journal of Australia* **215**(9), 420–426. | wrong intervention |
| (Sabri et al. 2019) | **Sabri B, Njie–Carr VPS, Messing JT, Glass N, Brockie T, Hanson G, Case J and Campbell JC** (2019) The weWomen and ourCircle randomized controlled trial protocol: A web–based intervention for immigrant, refugee and indigenous women with intimate partner violence experiences. *Contemporary Clinical Trials* **76**, 79–84. https://doi.org/10.1016/j.cct.2018.11.013. | ongoing |
| (Sabri et al. 2021) | **Sabri B, Glass N, Murray S, Perrin N, Case JR and Campbell JC** (2021) A technology–based intervention to improve safety, mental health and empowerment outcomes for immigrant women with intimate partner violence experiences: It’s weWomen plus sequential multiple assignment randomized trial (SMART) protocol. *BMC Public Health* **21**(1), 1956. https://doi.org/10.1186/s12889–021–11930–2. | ongoing |
| (Salihu et al. 2021) | **Salihu D, Wong EML and Kwan RYC** (2021) Effects of an african circle dance programme on internally displaced persons with depressive symptoms: A quasi–experimental study. *International Journal of Environmental Research and Public Health* **18**(2), 1–17. https://doi.org/10.3390/ijerph18020843. | wrong study design |
| (Sandahl et al. 2017) | **Sandahl H, Jennum P, Baandrup L, Poschmann IS and Carlsson J** (2017) Treatment of sleep disturbances in trauma–affected refugees: Study protocol for a randomised controlled trial. *Trials* **18**(1), 520. | duplicate |
| (Sarkadi et al. 2018) | **Sarkadi A, Adahl K, Stenvall E, Ssegonja R, Batti H, Gavra P, Fangstrom K and Salari R** (2018) Teaching Recovery Techniques: Evaluation of a group intervention for unaccompanied refugee minors with symptoms of PTSD in Sweden. *European Child and Adolescent Psychiatry* **27**(4), 467–479. https://doi.org/10.1007/s00787–017–1093–9. | wrong study design |
| (Sarkadi et al. 2020) | **Sarkadi A, Warner G, Salari R, Fangstrom K, Durbeej N, Lampa E, Baghdasaryan Z, Osman F, Gupta Lofving S, Perez Aronsson A, Feldman I, Sampaio F, Ssegonja R, Calam R, Bjarta A, Leiler A, Rondung E, Wasteson E, Oppedal B and Keeshin B** (2020) Evaluation of the Teaching Recovery Techniques community–based intervention for unaccompanied refugee youth experiencing post–traumatic stress symptoms (Swedish UnaccomPanied yOuth Refugee Trial; SUPpORT): Study protocol for a randomised controlled trial. *Trials* **21**(1), 63. https://doi.org/10.1186/s13063–019–3814–5. | duplicate |
| (Schaefer et al. 2022) | **Schaefer I, Hiller P, Milin S and Lotzin A** (2022) A multicenter, randomized controlled trial to compare the effectiveness of STARC–SUD (Skills Training in Affect Regulation—A Culture–sensitive approach) versus treatment as usual in trauma–exposed refugees with substance use problems. *Trials* **23**(1) https://doi.org/10.1186/s13063–022–06761–4. | duplicate |
| (Scheiber et al. 2019) | **Scheiber B, Greinz G, Hillebrand JB, Wilhelm FH and Blechert J** (2019) Resilience training for unaccompanied refugee minors: A randomized controlled pilot study. *Resilienztraining fur unbegleitete minderjahrige Fluchtlinge: Eine randomisiert–kontrollierte Pilotstudi,* **28**(3), 173–181. | no full text |
| (Schulz et al. 2006) | **Schulz PM, Resick PA, Huber LC and Griffin MG** (2006) The Effectiveness of Cognitive Processing Therapy for PTSD With Refugees in a Community Setting. *Cognitive and Behavioral Practice* **13**(4), 322–331. https://doi.org/10.1016/j.cbpra.2006.04.011. | wrong study design |
| (Schytt et al. 2020) | **Schytt E, Wahlberg A, Eltayb A, Small R, Tsekhmestruk N and Lindgren H** (2020) Community–based doula support for migrant women during labour and birth: Study protocol for a randomised controlled trial in Stockholm, Sweden (NCT03461640) *BMJ Open* **10**(2), 031290. https://doi.org/10.1136/bmjopen–2019–031290. | ongoing |
| (Scopetta et al. 1978) | **Scopetta MA** (1978) Ecological Structural Family Therapy with Cuban Immigrant Families. PhD dissertation, University of Miami, Florida. https://www.proquest.com/reports/ecological–structural–family–therapy–with–cuban/docview/63607788/se–2?accountid=14836. | no full text |
| (Sherman–Bien et al. 2011) | **Sherman–Bien SA, Askins M, Katz E, Dolgin M, Butler R, Fairclough D, Noll RB and Sahler OJ** (2011) A cross–cultural perspective of mothers of children with newly diagnosed cancer: Results of a multi–institutional randomized trial of maternal problem–solving skills training. *Pediatric Blood and Cancer* **56**(7), 1159. https://doi.org/10.1002/pbc.23141. | wrong population |
| (Shultz et al. 2019) | **Shultz JM, Verdeli H, Gómez Ceballos A, Hernandez LJ, Espinel Z, Helpman L, Neria Y and Araya R** (2019) A pilot study of a stepped–care brief intervention to help psychologically–distressed women displaced by conflict in Bogotá, Colombia. *Global Mental Health* 6. https://www.proquest.com/scholarly–journals/pilot–study–stepped–care–brief–intervention–help/docview/2321664325/se–2. | wrong study design |
| (Siddiqui et al. 2019) | **Siddiqui F, Lindblad U, Nilsson PM and Bennet L** (2019) Effects of a randomized, culturally adapted, lifestyle intervention on mental health among Middle–Eastern immigrants. *European Journal of Public Health* **29**(5), 888–894. | wrong intervention |
| (Sin et al. 2015) | **Sin M, Ibarra B, Tae T and Murphy P** (2015) Effect of a Randomized Controlled Trial Walking Program on Walking, Stress, Depressive Symptoms and Cardiovascular Biomarkers in Elderly Korean Immigrants*. Journal of Korean Biological Nursing Science* 17(2), 89–96. | wrong intervention |
| (Siriwardhana et al. 2013) | **Siriwardhana C, Adikari A, Van Bortel T, McCrone P and Sumathipala A** (2013) An intervention to improve mental health care for conflict–affected forced migrants in low–resource primary care settings: A WHO MhGAP–based pilot study in Sri Lanka (COM–GAP study). *Trials* **14**(1), 423. https://doi.org/10.1186/1745–6215–14–423. | ongoing |
| (Sjostrom et al. 2022) | **Sjostrom R, Kaev G and Soderstrom L** (2022) Evaluation of a Health–Management Course for Recently Settled Immigrants. *Journal of international migration and integration* **23**(1), 365–381. | wrong study design |
| (Slewa–Younan et al. 2020) | **Slewa–Younan S, McKenzie M, Thomson R, Smith M, Mohammad Y and Mond J** (2020) Improving the mental wellbeing of Arabic speaking refugees: An evaluation of a mental health promotion program. *BMC Psychiatry* **20**(1), 314. | wrong study design |
| (Smokowski and Bacallao, 2009) | **Smokowski PR and Bacallao M** (2009) Entre Dos Mundos/Between Two Worlds: Youth Violence Prevention for Acculturating Latino Families. *Research on Social Work Practice* **19**(2), 165–178. | wrong outcome |
| (Smyth et al. 2002) | **Smyth JM, Hockemeyer JR, Anderson C, Strandberg K, Koch M, O’Neill HK and McCammon SL** (2002) Structured writing about a natural disaster buffers the effect of intrusive thoughts on negative affect and physical symptoms. *Australasian Journal of Disaster and Trauma Studies* **2002**(1). https://www.proquest.com/scholarly–journals/structured–writing–about–natural–disaster–buffers/docview/42415838/se–2?accountid=14836. | wrong population |
| (Snodgrass et al. 1993) | **Snodgrass LL, Yamamoto J, Frederick CJ, Ton–That N, Foy DW, Chan L, Wu J, Hahn PH, Shinh DY, Nguyen LH, De Jonge J and Fairbanks LA** (1993) Vietnamese refugees with PTSD symptomatology: Intervention via a coping skills model. *Journal of Traumatic Stress* **6**(4), 569–575. | wrong study design |
| (Sobanski et al. 2021) | **Sobanski E, Hammerle F, Dixius A, Mohler E, Koudela–Hamila S, Ebner–Priemer U, Merz CJ, In–Albon T, Pollitt B, Christiansen H, Kolar D, Ocker S, Fischer N, Burghaus I and Huss M** (2021) START adolescents: Study protocol of a randomised controlled trial to investigate the efficacy of a low–threshold group treatment programme in traumatised adolescent refugees. *BMJ Open* **11**(12), e057968. https://doi.org/10.1136/bmjopen–2021–057968. | ongoing |
| (Son and 최현옥, 2011) | **Son C and 최현옥** (2011) The Effects of the Korean Mindfulness–Based Stress Reduction(K–MBSR) Program on Posttraumatic Stress Disorder Symptoms, Experiential Avoidance and Shame in North Korean Defectors. *Korean Journal of Health Psychology* **16**(3), 469–482. | no full text |
| (Sonderegger et al. 2011) | **Sonderegger R, Rombouts S, Ocen B and McKeever RS** (2011) Trauma rehabilitation for war–affected persons in northern Uganda: A pilot evaluation of the EMPOWER programme. *The British Journal of Clinical Psychology* **50**(3), 234–249. | wrong study design |
| (Sonne et al. 2013) | **Sonne C, Carlsson J, Elklit A, Mortensen EL and Ekstrom M** (2013) Treatment of traumatized refugees with sertraline versus venlafaxine in combination with psychotherapy—Study protocol for a randomized clinical trial. *Trials* **14**(101263253), 137. | duplicate |
| (Sonne et al. 2016) | **Sonne C, Carlsson J, Bech P, Elklit A and Mortensen EL** (2016) Treatment of trauma–affected refugees with venlafaxine versus sertraline combined with psychotherapy—A randomised study. *BMC Psychiatry* **16**(1), 383. https://doi.org/10.1186/s12888–016–1081–5. | wrong study design |
| (Sonne et al. 2021) | **Sonne C, Mortensen EL, Silove D, Palic S and Carlsson J** (2021) Predictors of treatment outcomes for trauma–affected refugees—Results from two randomised trials. *Journal of Affective Disorders* **282**, 194–202. https://doi.org/10.1016/j.jad.2020.12.095. | duplicate |
| (Stark et al. 2018) | **Stark L, Seff I, Assezenew A, Eoomkham J, Falb K and Ssewamala FM** (2018) Effects of a Social Empowerment Intervention on Economic Vulnerability for Adolescent Refugee Girls in Ethiopia. *The Journal of Adolescent Health: Official Publication of the Society for Adolescent Medicine* **62**(1), S15–S20. | wrong outcome |
| (Steel et al. 2023) | **Steel C, Young K, Akbar S, Chessell Z, Stevens A, Vann M and Arntz A** (2023) The treatment of PTSD in refugees and asylum seekers using imagery rescripting within an NHS setting. *Behavioural and cognitive psychotherapy* **51**(2), 119–132. https://doi.org/10.1017/S1352465822000650. | wrong study design |
| (Steil et al. 2021) | **Steil R, Lechner–Meichsner F, Johow J, Kruger–Gottschalk A, Mewes R, Reese J–P, Schumm H, Weise C, Morina N and Ehring T** (2021) Brief imagery rescripting vs. Usual care and treatment advice in refugees with posttraumatic stress disorder: Study protocol for a multi–center randomized–controlled trial. *European Journal of Psychotraumatology* **12**(1), 1872967. | ongoing |
| (Stiles et al. 2021) | **Stiles DA, Alaraudanjoki E, Wilkinson LR, Ritchie KL and Brown KA** (2021) Researching the Effectiveness of Tree of Life: An Imbeleko Approach to Counseling Refugee Youth. *Journal of Child and Adolescent Traum*a **14**(1), 123–139. https://doi.org/10.1007/s40653–019–00286–w. | ongoing |
| (Strupf et al. 2023) | **Strupf M, Wiechers M, Bajbouj M, Boge K, Karnouk C, Goerigk S, Kamp–Becker I, Banaschewski T, Rapp M, Hasan A, Falkai P, Jobst–Heel A, Habel U, Stamm T, Heinz A, Hoell A, Burger M, Bunse T, Hoehne E, Mehran N, Kaiser F, Hahn E, Plener P, Übleis A and Padberg F** (2023) Predicting treatment outcomes of the Empowerment group intervention for refugees with affective disorders: Findings from the MEHIRA project. *Journal of Affective Disorders* **323**, 241–250. https://doi.org/10.1016/j.jad.2022.11.050. | wrong study design |
| (Taylor–Piliae et al. 2006) | **Taylor–Piliae RE, Haskell WL, Waters CM and Froelicher ES** (2006) Change in perceived psychosocial status following a 12–week Tai Chi exercise programme. *Journal of Advanced Nursing* **54**(3), 313–329. https://doi.org/10.1111/j.1365–2648.2006.03809.x. | wrong study design |
| (Thabet et al. 2005) | **Thabet AA, Vostanis P and Karim K** (2005) Group crisis intervention for children during ongoing war conflict. *European Child and Adolescent Psychiatry* **14**(5), 262–269. https://doi.org/10.1007/s00787–005–0466–7. | wrong study design |
| (Tol et al. 2014) | **Tol WA, Komproe IH, Jordans MJD, Ndayisaba A, Ntamutumba P, Sipsma H, Smallegange ES, Macy RD and De Jong JTVM** (2014) School–based mental health intervention for children in war–affected Burundi: A cluster randomized trial. *BMC Medicine* **12**(1). | wrong population |
| (Tol et al. 2017) | **Tol WA, Greene MC, Likindikoki S, Misinzo L, Ventevogel P, Bonz AG, Bass JK and Mbwambo JKK** (2017) An integrated intervention to reduce intimate partner violence and psychological distress with refugees in low–resource settings: Study protocol for the Nguvu cluster randomized trial. *BMC Psychiatry* 17(1), 186. | duplicate |
| (Tol et al. 2018) | **Tol W, Augustinavicius J, Carswell K, Leku M, Adaku A, Brown F, Garcia–Moreno C, Ventevogel P, White R, Kogan C, Bryant R and van Ommeren M** (2018) Feasibility of a guided self–help intervention to reduce psychological distress in South Sudanese refugee women in Uganda. *World Psychiatry* **17**(2), 234–235. | duplicate |
| (Trilesnik et al. 2019) | **Trilesnik B, Altunoz U, Wesolowski J, Eckhoff L, Ozkan I, Loos K, Penteker G and Graef–Calliess IT** (2019) Implementing a need–adapted stepped–care model for mental health of refugees: Preliminary data of the state–funded project ‘Refukey’. *Frontiers in Psychiatry* **10**, 688. https://doi.org/10.3389/fpsyt.2019.00688. | wrong study design |
| (Tucker et al. 2021) | **Tucker C, Schieffer K, Lenz S and Smith S** (2021) Sunshine Circles: Randomized controlled trial of an attachment–based play group with preschool students who are at–risk. *Journal of Child and Adolescent Counseling* **7**(3), 161–175. | wrong population |
| (Turrini et al. 2022) | **Turrini G, Purgato M, Tedeschi F, Acarturk C, Anttila M, Au T, Carswell K, Churchill R, Cuijpers P, Friedrich F, Gastaldon C, Klein T, Kosters M, Lantta T, Nosè M, Ostuzzi G, Papola D, Popa M, Sijbrandij M, Tarsitani L, Todini L, Uygun E, Välimäki M, Walker L, Wancata J, White RG, Zanini E, van Ommeren M and Barbui C** (2022) Long–term effectiveness of Self–Help Plus in refugees and asylum seekers resettled in Western Europe: 12–month outcomes of a randomised controlled trial. *Epidemiology and psychiatric sciences* **31**. https://doi.org/10.1017/S2045796022000269. | duplicate |
| (Unlu et al. 2010) | **Unlu B, Riper H, van Straten A and Cuijpers P** (2010) Guided self–help on the Internet for Turkish migrants with depression: The design of a randomized controlled trial. *Trials* **11**(101263253), 101. | duplicate |
| (Uygun et al. 2020) | **Uygun E, Ilkkursun Z, Sijbrandij M, Aker AT, Bryant R, Cuijpers P, Fuhr DC, De Graaff AM, De Jong J, McDaid D, Morina N, Park A–L, Roberts B, Ventevogel P, Yurtbakan T and Acarturk C** (2020) Protocol for a randomized controlled trial: Peer–to–peer Group Problem Management plus (PM+) for adult Syrian refugees in Turkey. *Trials* **21**(1), 283. https://doi.org/10.1186/s13063–020–4166–x. | ongoing |
| (van der Heide et al. 2021) | **van der Heide I, van Wezel N, Blom M, Spreeuwenberg P, Deville WLJM and Francke AL** (2021) Effects of an educational intervention on health–related quality of life among family caregivers of people with dementia with a Turkish or Moroccan immigrant background: Insights from a cluster randomised controlled trial. *Patient Education and Counseling* **104**(5), 1168–1175. | wrong intervention |
| (van Loon et al. 2011) | **van Loon A, van Schaik D, Dekker J and Beekman A** (2011) Effectiveness of an intercultural module added to the treatment guidelines for Moroccan and Turkish patients with depressive and anxiety disorders. *BMC Psychiatry* **11**, 13. | ongoing |
| (Vasserman and Burda, 2018) | **Vasserman DS and Burda T** (2018) Thinking, Moving and Feeling: A Proposed Movement and Behavioral Intervention for Refugee Children with Trauma. PhD dissertation, William James College, Newton, Massachussets. https://www.proquest.com/dissertations–theses/thinking–moving–feeling–proposed–movement/docview/2193804375/se–2?accountid=14836. | wrong study design |
| (Velu et al. 2022) | **Velu ME, Martens I, Shahab M, de Roos C, Jongedijk RA, Schok M and Mooren T** (2022) Trauma–focused treatments for refugee children: Study protocol for a randomized controlled trial of the effectiveness of KIDNET versus EMDR therapy versus a waitlist control group (KIEM). *Trials* **23**(1), 347. https://doi.org/10.1186/s13063–022–06178–z. | ongoing |
| (Vibe Jespersen et al. 2012) | **Vibe Jespersen K and Vuust P** (2012) The effects of relaxation music listening on sleep quality in traumatized refugees: A pilot study. *Journal of Music Therapy* **49**(2), 205–229. | wrong study design |
| (Vidales, 1987) | **Vidales R** (1987) An educational intervention for displaced workers. PhD dissertation, California State University, Long Beach, California. https://www.proquest.com/dissertations–theses/educational–intervention–displaced–workers/docview/303536115/se–2?accountid=14836. | no full text |
| (Vindbjerg et al. 2014) | **Vindbjerg E, Klimpke C and Carlsson J** (2014) Psychotherapy with traumatised refugees—The design of a randomised clinical trial*. Torture: Quarterly Journal on Rehabilitation of Torture Victims and Prevention of Torture* **24**(1), 40–48. | duplicate |
| (Vitsukaieva and Horvat, 2018) | **Vitsukaieva K and Horvat M** (2018) Socio–pedagogical support of anxious children from internally displaced families. *Science and education* **2**, 101–106. | wrong study design |
| (Walter et al. 2021) | **Walter B, Indreboe H, Lukasse M, Henriksen L and Garnweidner–Holme L** (2021) Pregnant Women’s Attitudes Toward and Experiences With a Tablet Intervention to Promote Safety Behaviors in a Randomized Controlled Trial: Qualitative Study. *JMIR Formative Research* **5**(7). | wrong outcome |
| (Warner et al. 2020) | **Warner G, Durbeej N, Salari R, Fangstrom K, Lampa E, Baghdasaryan Z, Osman F, Gupta Lofving S, Perez Aronsson A, Feldman I, Sampaio F, Ssegonja R, Bjarta A, Rondung E, Leiler A, Wasteson E, Calam R, Oppedal B, Keeshin B and Sarkadi A** (2020) Evaluation of the teaching recovery techniques community–based intervention for accompanied refugee children experiencing post–traumatic stress symptoms (Accompanied refugeeS In Sweden Trial; ASsIST): Study protocol for a cluster randomised controlled trial. *BMJ Open* **10**(7), e035459. | study stopped |
| (Weise et al. 2021) | **Weise C, Grupp F, Reese J–P, Schade–Brittinger C, Ehring T, Morina N, Stangier U, Steil R, Johow J and Mewes R** (2021) Efficacy of a Low–threshold, Culturally–Sensitive Group Psychoeducation Programme for Asylum Seekers (LoPe): Study protocol for a multicentre randomised controlled trial. *BMJ Open* **11**(10), e047385. https://doi.org/10.1136/bmjopen–2020–047385. | ongoing |
| (Wilker et al. 2020) | **Wilker S, Catani C, Wittmann J, Preusse M, Schmidt T, May T, Ertl V, Doering B, Rosner R, Zindler A and Neuner F** (2020) The efficacy of Narrative Exposure Therapy for Children (KIDNET) as a treatment for traumatized young refugees versus treatment as usual: Study protocol for a multi–center randomized controlled trial (YOURTREAT). *Trials* **21**(1), 185. | duplicate |
| (Wilker et al. 2020) | **Wilker S, Catani C, Wittmann J, Preusse M, Schmidt T, May T, Ertl V, Doering B, Rosner R, Zindler A and Neuner F** (2020) The efficacy of Narrative Exposure Therapy for Children (KIDNET) as a treatment for traumatized young refugees versus treatment as usual: Study protocol for a multi–center randomized controlled trial (YOURTREAT) *Trials* **21**(1), 185. | ongoing |
| (Williamson et al. 2014) | **Williamson AA, Knox L, Guerra NG and Williams KR** (2014) A Pilot Randomized Trial of Community–Based Parent Training for Immigrant Latina Mothers. *American Journal of Community Psychology* **53**(1–2), 47–59. https://doi.org/10.1007/s10464–013–9612–4. | wrong population |
| (Winkler et al. 2015) | **Winkler N, Ruf–Leuschner M, Ertl V, Pfeiffer A, Schalinski I, Ovuga E, Neuner F and Elbert T** (2015) From war to classroom: PTSD and depression in formerly abducted youth in Uganda. *Frontiers in Psychiatry* **6**. | wrong population |
| (Wittmann et al. 2022) | **Wittmann J, Gros M, Catani C, Schmidt T, Neldner S, Wilker S, May T, Ertl V, Rosner R, Zindler A, Odenwald M and Neuner F** (2022) The efficacy of Narrative Exposure Therapy for Children (KIDNET) as a treatment for traumatized young refugees versus treatment as usual: Update to the study protocol for the multi–center randomized controlled trial YOURTREAT. *Trials* **23**(1), 360. https://doi.org/10.1186/s13063–022–06288–8. | wrong study design |
| (Wong et al. 2013) | **Wong JYH, Tiwari A, Fong DYT, Yuen KH, Humphreys J and Bullock L** (2013) Intimate partner violence, depressive symptoms and immigration status: Does existing advocacy intervention work on abused immigrant women in the Chinese community?. *Journal of Interpersonal Violence* **28**(11), 2181–2202. | wrong population |
| (Wong et al. 2020) | **Wong MHM, Keng S–L, Buck PJ, Suthendran S, Wessels A and Ostbye T** (2020) Effects of Mental Health Paraprofessional Training for Filipina Foreign Domestic Workers in Singapore. *Journal of Immigrant and Minority Health* **22**(3), 571–579. https://doi.org/10.1007/s10903–019–00907–4. | wrong outcome |
| (Wu et al. 2020) | **Wu S, Marsiglia FF, Ayers S, Cutrin O and Vega–Lopez S** (2020) Familial Acculturative Stress and Adolescent Internalizing and Externalizing Behaviors in Latinx Immigrant Families of the Southwest. *Journal of Immigrant and Minority Health* **22**(6), 1193–1199. https://doi.org/10.1007/s10903–020–01084–5. | wrong study design |
| (Yankey and Biswas, 2019) | **Yankey T and Biswas UN** (2019) Impact of life skills training on psychosocial well–being of Tibetan refugee adolescents. *International Journal of Migration, Health and Social Care* **15**(4), 272–284. | wrong outcome |
| (Yeomans et al. 2010) | **Yeomans PD, Forman EM, Herbert JD and Yuen E** (2010) A randomized trial of a reconciliation workshop with and without PTSD psychoeducation in Burundian sample. *Journal of Traumatic Stress* **23**(3), 305–312. | wrong population |
| (Yu et al. 2014) | **Yu X, Stewart SM, Chui JPL, Ho JLY, Li ACH and Lam TH** (2014) A Pilot Randomized Controlled Trial to Decrease Adaptation Difficulties in Chinese New Immigrants to Hong Kong. *Behavior Therapy* **45**(1), 137–152. https://doi.org/10.1016/j.beth.2013.10.003. | wrong outcome |
| (김승현 and 최빛내, 2013) | **김승현 and 최빛내** (2013) The Effectiveness of PTSD Program of North Korean Refugees: For North Korean Female Refugees. *The Korean Journal of Woman Psychology* **18**(4), 533–548. | no full text |
| (宋艳丽 et al. 2017) | **宋艳丽, 解颖, 刘伟, 耿庆妍, 吕中媛, 王大遒 and 张丽娟** (2017) 沈阳市城市空巢“候鸟”老人心理健康干预效果及对策研究. *Chinese Nursing Research* **31**(16), 1955–1960. | wrong population |

**Table S3: Graphs publication year – international migrants**

**Table S4: Classification of Interventions**

**Established psychotherapies**

**CBT–based/inspired**

Acceptance and Commitment Therapy (ACT)

Basic Body Awarness Therapy + CBT

CBT (Cognitive Behavior Therapy)

CBT and pharmacological intervention

CBT–BF (CBT–Bio Feedback)

CBT+mindfulness

Cognitive Processing therapy (CPT)

iCBT (internet–based Cognitive Behaviour Therapy

**Trauma–focused psychotherapies**

EMDR (Eye Movement Desensitization and Reprocessing)

Imagery Reharsal Therapy (IRT)

KIDNET (Narrative Exposure Therapy for Children)

NET (Narrative Exposure Therapy)

Trauma focused psychotherapy

**Psychotherapy (other)**

Intensive psychotherapy and case management (IPCM)

Interpersonal therapy (IPT)

Problem–solving therapy

Psychodynamic therapy

Stabilisation therapy

**Counselling and psychosocial support**

**Counselling**

Expressive group counseling

Supportive counselling

Value–based counselling

Trauma counselling

Psychoeducation + counselling

**Psychosocial support**

Care management

CETA (Common Elements Treatment Approach)

CROP groups

General psychosocial intervention

Life skills training

Mindfulness

Multilevel strengths–based intervention

Need–satisfaction intervention

Psychoeducation

Resilience–based intervention

Stepped care and Collaborative Model (SCCM)

Stress management

Teaching Recovery Techniques

Transdiagnostic group intervention

Trauma–focused group intervention

WHO psychosocial interventions (PM+, SH+, DWM, EASE)

**Creative–expressive**

Creative–expressive intervention

Expressive Writing

Game–based learning intervention

Laugher therapy

Music Therapy Program

Play Therapy

Structured sensory intervention

**Family/parenting interventions**

CAFES: Coffee and Family Education and Support

Family–based intervention

Parental training

Parenting and family skills intervention

Table S5: RoB-2 assessment for anxiety, depression, PTSD, and psychological distress
