# Supplementary material for: Mapping the evidence on psychosocial interventions for migrant populations: Descriptive analysis of a living database of randomized studies
Source: Glob Ment Health (Camb). 2024 Mar 8;11:e35. doi: 10.1017/gmh.2024.33 (PMC10988138; doi:10.1017/gmh.2024.33)
Supplement: Peer review history

# Editor report: Recommendation: Mapping the evidence on psychosocial interventions for migrant populations: descriptive analysis of a living database of randomized studies — R0/PR2

**DOI:** 10.1017/gmh.2024.33.pr2

**Affiliations:** Program on Forced Migration and Health, Columbia University Mailman School of Public Health, United States

Thank you for submitting this paper to Global Mental Health. We have received feedback from two reviewers, both of whom were enthusiastic about the contribution and innovation of this manuscript. They have also identified some aspects of the manuscript that could be improved and have made some constructive recommendations, which I encourage the authors to consider. Specifically, both reviewers raised concerns about how the population(s) is defined and have asked from some clarifications in terminology. They have also raised some questions about the study design, the database’s scope (e.g., which conditions are included, which types of studies are included), and have provided several minor comments.

# Editor report: Decision: Mapping the evidence on psychosocial interventions for migrant populations: descriptive analysis of a living database of randomized studies — R0/PR3

**DOI:** 10.1017/gmh.2024.33.pr3

**Affiliations:** London School of Hygiene & Tropical Medicine, United Kingdom of Great Britain and Northern Ireland

No accompanying comment.

# Editor report: Recommendation: Mapping the evidence on psychosocial interventions for migrant populations: descriptive analysis of a living database of randomized studies — R1/PR5

**DOI:** 10.1017/gmh.2024.33.pr5

**Affiliations:** Program on Forced Migration and Health, Columbia University Mailman School of Public Health, United States

Thank you for submitting the revised version of this manuscript. The reviewer has suggested a few additional minor revisions that we hope you will consider.

# Editor report: Decision: Mapping the evidence on psychosocial interventions for migrant populations: descriptive analysis of a living database of randomized studies — R1/PR6

**DOI:** 10.1017/gmh.2024.33.pr6

**Affiliations:** London School of Hygiene & Tropical Medicine, United Kingdom of Great Britain and Northern Ireland

No accompanying comment.

# Editor report: Recommendation: Mapping the evidence on psychosocial interventions for migrant populations: descriptive analysis of a living database of randomized studies — R2/PR8

**DOI:** 10.1017/gmh.2024.33.pr8

**Affiliations:** Program on Forced Migration and Health, Columbia University Mailman School of Public Health, United States

Thank you for revising the manuscript according to the reviewer’s remaining suggestions. We are pleased to accept this important manuscript for publication. Congratulations to the authors.

# Editor report: Decision: Mapping the evidence on psychosocial interventions for migrant populations: descriptive analysis of a living database of randomized studies — R2/PR9

**DOI:** 10.1017/gmh.2024.33.pr9

**Affiliations:** London School of Hygiene & Tropical Medicine, United Kingdom of Great Britain and Northern Ireland

No accompanying comment.
